# Encephalitis lethargica: clinical features and aetiology

**DOI:** 10.1093/braincomms/fcae347

**Published:** 2024-10-04

**Authors:** Jonathan P Rogers, Tomas Mastellari, Alex J Berry, Kieron Kumar, Ella Burchill, Anthony S David, Glyn Lewis, Andrew Lees, Michael S Zandi

**Affiliations:** Division of Psychiatry, University College London, London W1T 7NF, UK; Department of Neuropsychiatry, National Hospital for Neurology and Neurosurgery, London WC1N 3BG, UK; University Lille, Inserm, CHU Lille, U1172—LilNCog—Lille Neuroscience & Cognition, F-59000 Lille, France; Department of Neuropsychiatry, National Hospital for Neurology and Neurosurgery, London WC1N 3BG, UK; South London and Maudsley NHS Foundation Trust, London BR3 3BX, UK; Division of Psychiatry, University College London, London W1T 7NF, UK; Institute of Mental Health, University College London, London W1T 7NF, UK; Division of Psychiatry, University College London, London W1T 7NF, UK; Department of Clinical and Movement Neurosciences, University College London, London WC1N 3BG, UK; Department of Neuroinflammation, National Hospital for Neurology and Neurosurgery, London WC1N 3BG, UK; Department of Neuroinflammation, University College London, London, WC1N 3BG, UK

**Keywords:** encephalitis lethargica, post-encephalitis, epidemic encephalitis, influenza, NMDA receptor encephalitis

## Abstract

Encephalitis lethargica, an epidemic neurological illness, typically involved a severe sleep disorder and progressive parkinsonism. A century later, our understanding relies on seminal descriptions, more recent historical research and the study of small numbers of possible sporadic cases. Theories around infection, environmental toxins, catatonia and autoimmune encephalitis have been proposed. We aimed to describe the presentation of encephalitis lethargica and test these diagnostic and aetiological theories. Subjects with encephalitis lethargica were identified in the archives of the National Hospital for Neurology and Neurosurgery, UK between 1918 and 1946. Case notes were examined to establish illness temporality, clinical features and cerebrospinal fluid results. Controls from the archives were identified for 10% of cases, matching on discharge year, sex and neurologist. Clinical presentation was compared to modern diagnostic criteria for encephalitis lethargica, catatonia and autoimmune encephalitis. In a case–control design, a multilevel logistic regression was conducted to ascertain whether cases of encephalitis lethargica were associated with febrile illnesses and with environmental exposures. Six hundred and fourteen cases of encephalitis lethargica and 65 controls were identified. Cases had a median age of 29 years (interquartile range 18) and a median time since symptomatic onset of 3.00 years (interquartile range 3.52). Motor features were present in 97.6%, cranial nerve findings in 91.0%, ophthalmological features in 77.4%, sleep disorders in 66.1%, gastrointestinal or nutritional features in 62.1%, speech disorders in 60.8% and psychiatric features in 53.9%. Of the 167 cases who underwent lumbar puncture, 20 (12.0%) had a pleocytosis. The Howard and Lees criteria for encephalitis lethargica had a sensitivity of 28.5% and specificity of 96.9%. Among the cases, 195 (31.8%, 95% confidence interval 28.1–35.6%) had a history of febrile illness within one calendar year prior to illness onset, which was more common than among the controls (odds ratio 2.70, 95% confidence interval 1.02–7.20, *P* = 0.05), but there was substantial reporting bias. There was no evidence that occupational exposure to solvents or heavy metals was associated with encephalitis lethargica. Two hundred and seventy-six (45.0%) of the cases might meet criteria for possible autoimmune encephalitis, but only 3 (0.5%) might meet criteria for probable NMDA receptor encephalitis. Only 11 cases (1.8%) met criteria for catatonia. Encephalitis lethargica has a distinct identity as a neuropsychiatric condition with a wide range of clinical features. Evidence for a relationship with infectious or occupational exposures was weak. Autoimmune encephalitis may be an explanation, but typical cases were inconsistent with NMDA receptor encephalitis.

## Teaser

Rogers *et al*. report 614 historical cases of encephalitis lethargica. Motor and cranial nerve abnormalities were present in over 90%. A total of 31.8% had a preceding febrile illness. There was no evidence for solvent or heavy metal exposure. A total of 45.0% of cases may meet criteria for possible autoimmune encephalitis.

## Graphical Abstract

**Graphical Abstract:**
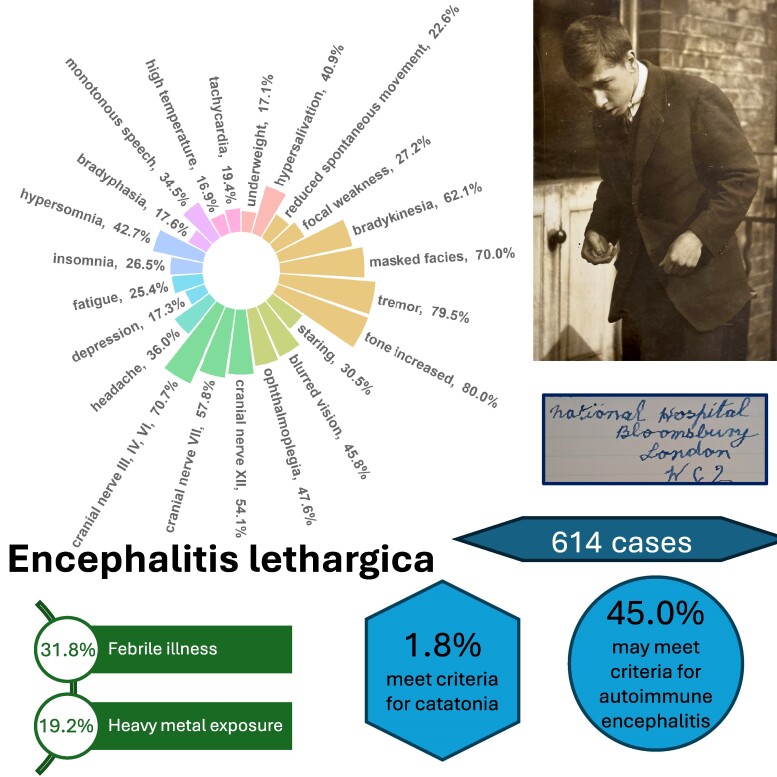

## Introduction

‘Patient lies in bed looking like a pillar of salt. There is a sphinx-like immobility about her countenance. Her face is as expressionless as if it were carved out of granite.’

Extract from the case notes of one of Dr Kinnier Wilson’s patients, 1927

Encephalitis lethargica, an epidemic ‘sleepy sickness’, was first fully described by the physician Constantin von Economo in his 1917 article,^1^ although it may have appeared as early as 1915.^2^ Neurological epidemics were not unknown and contemporaneous comparisons were made to conditions such as ‘the English sweats’ (16th century Europe), ‘Kriebelkrankheit’ (15th and 16th century Germany) and ‘Raphania’ (18th century Sweden),^3^ but the extent of the encephalitis lethargica epidemic was unprecedented. More than a million people suffered from severe neurological disease, and there were at least half a million fatalities.^4^ The last survivor from the epidemic died in the UK in 2002,^5^ but similar sporadic cases have occasionally been reported since.^6,7^

The clinical presentation typically began with non-specific prodromal symptoms, such as headache, fever and malaise.^8^ Severe sleep abnormalities would follow rapidly thereafter, consisting of severe hypersomnia or insomnia, lasting several weeks.^8^ A post-acute illness often developed between one and five years later, consisting of a slowly progressive parkinsonian disorder, sometimes with oculogyric crises and florid neuropsychiatric symptoms.^2^ Robust data on the frequency of clinical features and the time course of the illness have sometimes been lacking, however. In recent years, Howard and Lees^6^ endeavoured to define diagnostic criteria for encephalitis lethargica, but these have not undergone external validation.

Von Economo saw his original cases in Vienna, but there had likely already been some spread across Europe, appearing at the French front at Verdun in 1916.^9^ Cases were subsequently reported in North America, Central America, Japan, Australia, the Middle East, South Africa and West Africa.^8^ The first cases in the UK were reported in London in the spring of 1918^10^ and a number of cases were admitted to the National Hospital for the Cure of the Paralysed and Epileptic (now the National Hospital for Neurology and Neurosurgery), although the epidemic had less impact on the case mix of the wards than had been seen in the preceding four years by the large number of cases of war neuroses. Kinnier Wilson^11^ writing in 1928 described ‘easy gradations from somnolence to sopor, sopor to stupor and stupor to coma’. Edward Farquhar Buzzard, addressing the Medical Society of London on encephalitis lethargica in 1924, noted, ‘Our interest was aroused by the recent outbreak in our midst of a disease which we regarded as a stray-visitor, whose sojourn might not unreasonably be expected to be of short duration, and whose back we should not be sorry to see. Events have not justified this expectation, as our guest is still outstaying his welcome[…]’.

A range of other case series has been published describing the clinical presentation. These have often been comparatively small, have lacked quantitative data on prevalence of clinical features, or have merely classified cases into syndromes without delineating specific clinical features.^7,12-16^ In reports of more recent, sporadic cases of apparent encephalitis lethargica, it is not clear whether the pathophysiology of the disease being described is the same as in the epidemic cases. The real mystery behind encephalitis lethargica lies in its aetiology, which—despite its rapid emergence and decline—after 100 years of research remains unsolved.^2^ Yet understanding why this disease emerged is critical to preventing a recurrence. The macroscopic neuropathological findings included arterial or venous occlusion and petechial bleeding of the basal ganglia, midbrain, pons or cerebellum.^8^ Cases of post-encephalitic parkinsonism show pallor and atrophy of the substantia nigra.^17^ Microscopic findings include neurofibrillary tangles and tau-positive glial inclusions.^17^ Given its epidemic spread, contemporaneous theories included environmental and infective agents. In terms of environmental agents, it has been hard to identify a toxin with the distribution over time and geography that would be required to cause encephalitis lethargica,^18^ not least given all the migration of people, arms and supplies that was taking place in the First World War.

In terms of infectious agents, there was some early speculation of entry of a pathogen to the central nervous system (CNS) via the nasal passages.^19^ One candidate phenomenon of interest is the 1918 Spanish influenza pandemic, which as well as being approximately coincident, shared clinical features with the febrile prodrome of many cases of encephalitis lethargica.^9^ Outbreaks of both diseases tended to occur in the Winter, unlike some other forms of encephalitis with summer peaks.^20,21^ While perhaps the most discussed aetiological theory of the last century, there are several epidemiological problems with it. The first of these is the timing of the emergence of the conditions, as the influenza pandemic is classically conceived as starting in March 1918, although some have postulated that reports of respiratory illnesses in Europe in 1916–17 suggest an earlier origin.^22^ There is also the issue that influenza spread from America to Europe, unlike encephalitis lethargica, which seemed to move in the opposite direction. However, this is not conclusive, given that influenza may have evolved unpredictably and diagnosis relied purely on clinical means.^23^ One interesting comparison has been made between Western Samoa, which imported the influenza pandemic and experienced cases of encephalitis lethargica, and American Samoa (only 70 km away), which instituted tight quarantine to avoid the influenza outbreak and also eluded encephalitis lethargica.^4^ Studies of victims of the 1918 influenza pandemic have investigated whether the virus affected organs other than the lungs, finding that—while multiple organ failure was commonly present at death—the histology indicated infectious disease solely in the lungs.^22^ Subsequent laboratory studies have failed to identify influenza RNA in archival brain tissue from patients with encephalitis lethargica,^24,25^ though criticism of these methods has included the suitability of the PCR technique and the age of the samples.^22,24^ It also does not rule out a ‘hit-and-run mechanism’ for the virus.^25^

One such mechanism could be CNS autoimmunity, which could be initiated by a pathogen that promotes an immune response, for instance via molecular mimicry.^26^ In 2004, Dale *et al*.,^7^ pursuing a hypothesis of postinfectious autoimmunity similar to Sydenham’s chorea, published a series of 20 new cases of supposed encephalitis lethargica, mainly in children, finding that 95% had autoantibodies that reacted to human basal ganglia antigens on western immunoblotting. More recent research on anti-basal ganglia antibodies, however, has questioned their pathological relevance due to the measurement techniques causing artefactual and clinically irrelevant binding.^27^ Dale followed up with a further study in which the serum of 20 contemporary paediatric patients with a phenotype consistent with encephalitis lethargica was tested for antibodies to the NMDA receptor.^28^ Ten of these patients had evidence for the autoantibodies in serum, of whom six had positive cerebrospinal fluid (CSF) samples. Those who had positive antibodies tended to have agitation, hyperkinetic dyskinesias, seizures and insomnia. This has led some to suggest that epidemic encephalitis lethargica may in fact have been NMDA receptor encephalitis.^2,29^ A subsequent study by the same group identified 12 paediatric patients with parkinsonism, dystonia and chorea who had serum antibodies to the dopamine D_2_ receptor.^30^

The final diagnostic theory we consider is that encephalitis lethargica is a form of catatonia. This is not an aetiological theory, as catatonia can appear in the context of a wide range of psychiatric and general medical conditions,^31^ although it is particularly common in NMDA receptor encephalitis.^32^ However, catatonia does have distinctive treatments, notably benzodiazepines and electroconvulsive therapy,^33^ which—if there were a strong relationship with encephalitis lethargica—may be of therapeutic relevance. Catatonia was undoubtedly a feature of ‘some’ cases of the original encephalitis lethargica epidemic,^34^ and there are more recent cases of catatonia that have been thought to be related to sporadic cases of encephalitis lethargica.^35-37^ The more radical contention is that encephalitis lethargica and catatonia are so similar^38^ that they may be regarded as essentially the same condition^39,40^ with different nomenclature being solely a reflection of a ‘conflict of paradigms’ between psychiatric and neurological disorders.^41^ This assertion could be tested by measuring empirical observations of epidemic cases of encephalitis lethargica against modern criteria for catatonia.

Given the uncertainty over the presentation and aetiology of encephalitis lethargica, particularly among epidemic cases, we conducted a study of archival case notes to examine the presentation and various aetiological theories of encephalitis lethargica. Specifically, this study aimed to (i) describe the demographic and clinical characteristics of patients with encephalitis lethargica; (ii) assess the validity of the Howard and Lees criteria for encephalitis lethargica; and (iii) examine the extent to which encephalitis lethargica may be explained by other disorders, specifically influenza, occupational exposures, catatonia and autoimmune encephalitis.

## Materials and methods

### Study design and setting

We conducted a case–control study using the Queen Square Archives. These archives contain all the inpatient case notes from 1863 to 1946 from the hospital that is now known as the National Hospital for Neurology and Neurosurgery, London, UK, which is a specialized centre that accepted referrals from general practitioners and physicians from London and around the country. The case notes contain demographic and hospital admission details, clinical history, physical examination findings with a focus on the neurological system, physical observation charts, final diagnosis and, in some cases, further investigations. Data were collected from the archives between July 2021 and April 2023. Approval for the study was obtained from the Queen Square Archives Committee. Given that the study used historical records from deceased patients, it was considered exempt from formal ethical approval under University College London’s policy. The manuscript was written according to the STROBE guidelines, and the STROBE checklist is included in Supplementary Table 1.

### Participants

A search was conducted in the final clinical diagnoses for ‘encephalitis’. The first author reviewed the diagnoses and excluded those that were unrelated to encephalitis lethargica (e.g. cerebellar encephalitis and post-diphtheritic encephalitis). Inclusion criteria were that subjects must have a diagnosis listed as encephalitis lethargica or a likely synonym and adequate notes detailing the history and physical examination findings. Exclusion criteria were possible or probable diagnoses or neurological comorbidity in the main clinical diagnosis. Where a patient had more than one admission with encephalitis lethargica, only data from the first admission were used, as notes on subsequent admissions tended to be brief and it was unclear which symptoms persisted.

A comparison group was identified by selecting 10% of case note volumes at random and—for every case in each selected volume—taking the next non-encephalitis lethargica patient in the volume. Given that case note volumes were specific to consultant neurologist, year of discharge and the patient’s sex, these were considered to be the matching variables.

### Data extraction

Data were extracted from the case notes by one of four authors (J.P.R., A.J.B., K.K. and T.M.) in the same way for cases and controls. Data were extracted from structured fields and the free text onto a spreadsheet for variables including demographic and admission details, disease timings, certain comorbidities, motor features, cranial nerve findings, ophthalmological features, sleep disorders, gastrointestinal and nutritional features, speech disorders, psychiatric features, cognitive features, altered consciousness, physical observations and CSF results. A full list of variables, their definitions and derivations is shown in Supplementary Table 2. Occupation was classified according to the major groups of the International Standard Classification of Occupations 8 (ISCO-08)^42^ and socioeconomic status was derived from occupation based on the National Statistics Socio-Economic Classification (NS-SEC).^43^ In addition, the authors collected any miscellaneous observations during review of the case notes that they thought to be of particular interest. Signs and symptoms were recorded as being present if they were reported at any time in the disease course; the lack of mention of a clinical feature was assumed to indicate its absence.

### Statistical analysis

To assess interrater reliability, the four authors conducting the data extraction examined 10 of the same patients. Once all non-binary variables and variables without any variability in the interrater reliability dataset were removed, kappa was calculated from the combined dataset.

Basic descriptive statistics were calculated, presenting numbers and percentages for discrete variables. Continuous variables were summarized using mean and standard deviation for normally distributed variables and median and interquartile range (IQR) for non-normally distributed variables. A heat map was generated to show the number of patients in each of the UK counties.

The validity of the Howard and Lees diagnostic criteria for encephalitis lethargica^6^ was assessed with respect to their ability to discriminate between the cases and controls, as ascertained by the contemporaneous diagnoses, by calculating sensitivity, specificity and area under the receiver operator characteristics (ROC) curve with 95% confidence intervals (CIs). The numbers of patients who met the DSM-5-TR diagnostic criteria for catatonia, the Graus criteria for possible autoimmune encephalitis and the Graus criteria for probable NMDA receptor encephalitis were calculated.^44^ The diagnostic criteria applied are listed in Table 1.

**Table 1:** Diagnostic criteria applied for encephalitis lethargica, catatonia and autoimmune encephalitis

Howard and Lees criteria for encephalitis lethargica^6^
‘An acute or subacute encephalitic illness which has as part of its clinical picture at least three of the following major criteria: (1) signs of basal ganglia involvement, (2) oculogyric crises, (3) ophthalmoplegia, (4) obsessive-compulsive behaviour, (5) akinetic mutism, (6) central respiratory irregularities, and (7) somnolence and/or sleep inversion.’
DSM-5-TR criteria for catatonia^45^
‘Catatonia is defined as the presence of three (or more) of the following symptoms: 1.Stupor (i.e., no psychomotor activity; not actively relating to environment). 2.Catalepsy (i.e., passive induction of a posture held against gravity). 3.Waxy flexibility (i.e., slight, even resistance to positioning by examiner). 4.Mutism (i.e., no, or very little, verbal response [exclude if known aphasia]). 5.Negativism (i.e., opposition or no response to instructions or external stimuli). 6.Posturing (i.e., spontaneous and active maintenance of a posture against gravity). 7.Mannerism (i.e., odd, circumstantial caricature of normal actions). 8.Stereotypy (i.e., repetitive, abnormally frequent, non-goal-directed movements). 9.Agitation, not influenced by external stimuli. 10.Grimacing. 11.Echolalia (i.e., mimicking another’s speech). 12.Echopraxia (i.e., mimicking another’s movements).’
Graus criteria for possible autoimmune encephalitis^44^
Diagnosis can be made when all three of the following criteria have been met:
1. Subacute onset (rapid progression of <3 months) of working memory deficits (short-term memory loss), altered mental status or psychiatric symptoms 2. At least one of the following: • New focal CNS findings • Seizures not explained by a previously known seizure disorder • CSF pleocytosis (white blood cell count of more than five cells per mm^3^) • MRI features suggestive of encephalitis 3. Reasonable exclusion of alternative causes
Graus criteria for probable NMDA receptor encephalitis^44^
Diagnosis can be made when all three of the following criteria have been met:
1. Rapid onset (<3 months) of at least four of the six following major groups of symptoms: • Abnormal (psychiatric) behaviour or cognitive dysfunction • Speech dysfunction (pressured speech, verbal reduction, mutism) • Seizures • Movement disorder, dyskinesias or rigidity/abnormal postures • Decreased level of consciousness • Autonomic dysfunction or central hypoventilation 2. At least one of the following laboratory study results: • Abnormal EEG (focal or diffuse slow or disorganized activity, epileptic activity, or extreme delta brush) • CSF with pleocytosis or oligoclonal bands 3. Reasonable exclusion of other disorders (appendix)
Diagnosis can also be made in the presence of three of the above groups of symptoms accompanied by a systemic teratoma

To assess whether febrile illnesses and occupational exposures were associated with encephalitis lethargica, an unconditional mixed-effects logistic regression was performed, adjusting for the matching variables (year of discharge and sex as fixed effects and consultant as a random effect), which we term Model 1. This use of unconditional logistic regression has been advocated as a means to adjust for any bias introduced by matching in a case–control study while maximizing precision of estimates.^46^ In Model 2, age was added as a fixed effect.

The analysis was conducted in *Stata* version 17.0, using the ‘statplot’, ‘spmap’, ‘shp2dta’, ‘vioplot’, ‘heatplot’, ‘palettes’, ‘colrspace’ and ‘melogit’ packages. A circular bar chart of clinical features was created in *R* version 4.3.1 using the ‘ggplot2’ package version 3.4.3. Two-tailed statistical tests were used, and the threshold for statistical significance was set to *P* < 0.05.

## Results

### Identification of cases and controls

**Figure 1:**
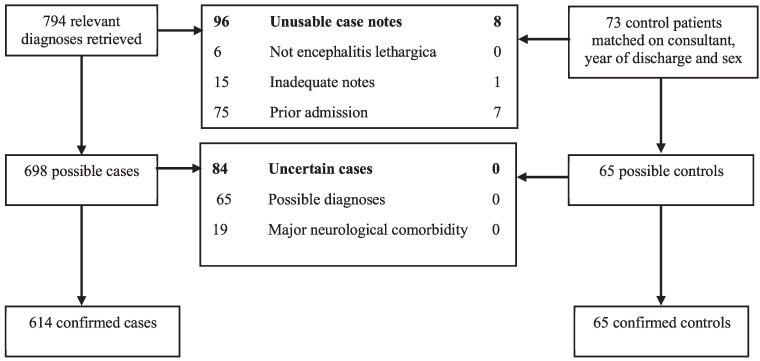
Flowchart illustrating identification of cases and controls.

The authors retrieved 794 case notes of patients with diagnoses relevant to encephalitis lethargica, which after applying the eligibility criteria left 614 confirmed cases, as shown in Fig. 1. This compared to just 294 cases of Parkinson’s disease (paralysis agitans) admitted to the hospital between 1918 and 1946. After applying eligibility criteria to the 73 matched control patients, 65 confirmed non-encephalitis controls were identified. Examples of the case notes from the first and last admitted patients are shown in Supplementary Figs 1 and 2. The kappa statistic for interrater reliability for the combined binary data extraction fields between the four authors who coded the case notes was 0.73, corresponding to substantial agreement.^47^

### Sociodemographic and hospital admission details

**Figure 2:**
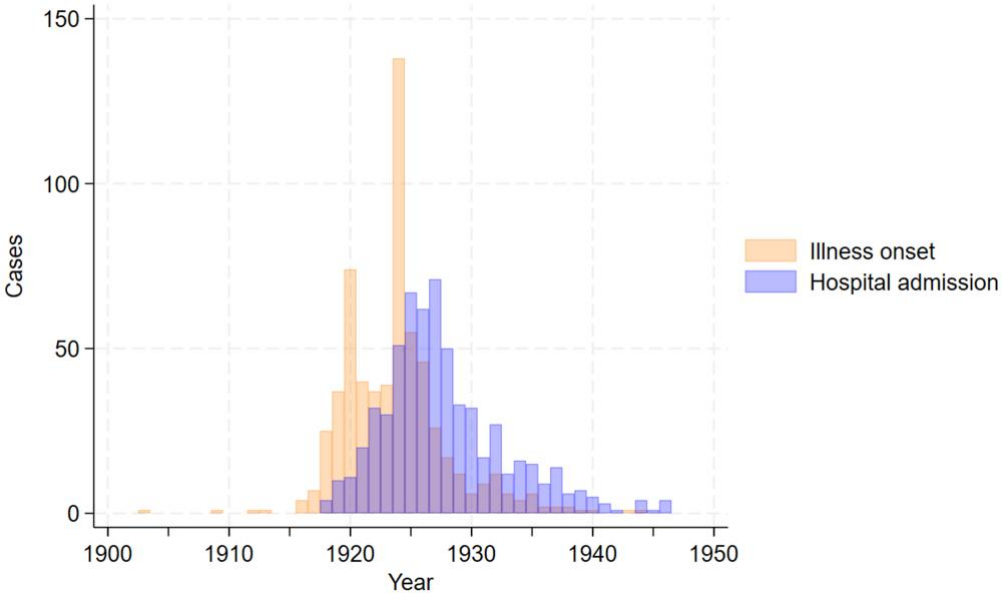
Bar chart of years of illness onset and hospital admission.

**Figure 3:**
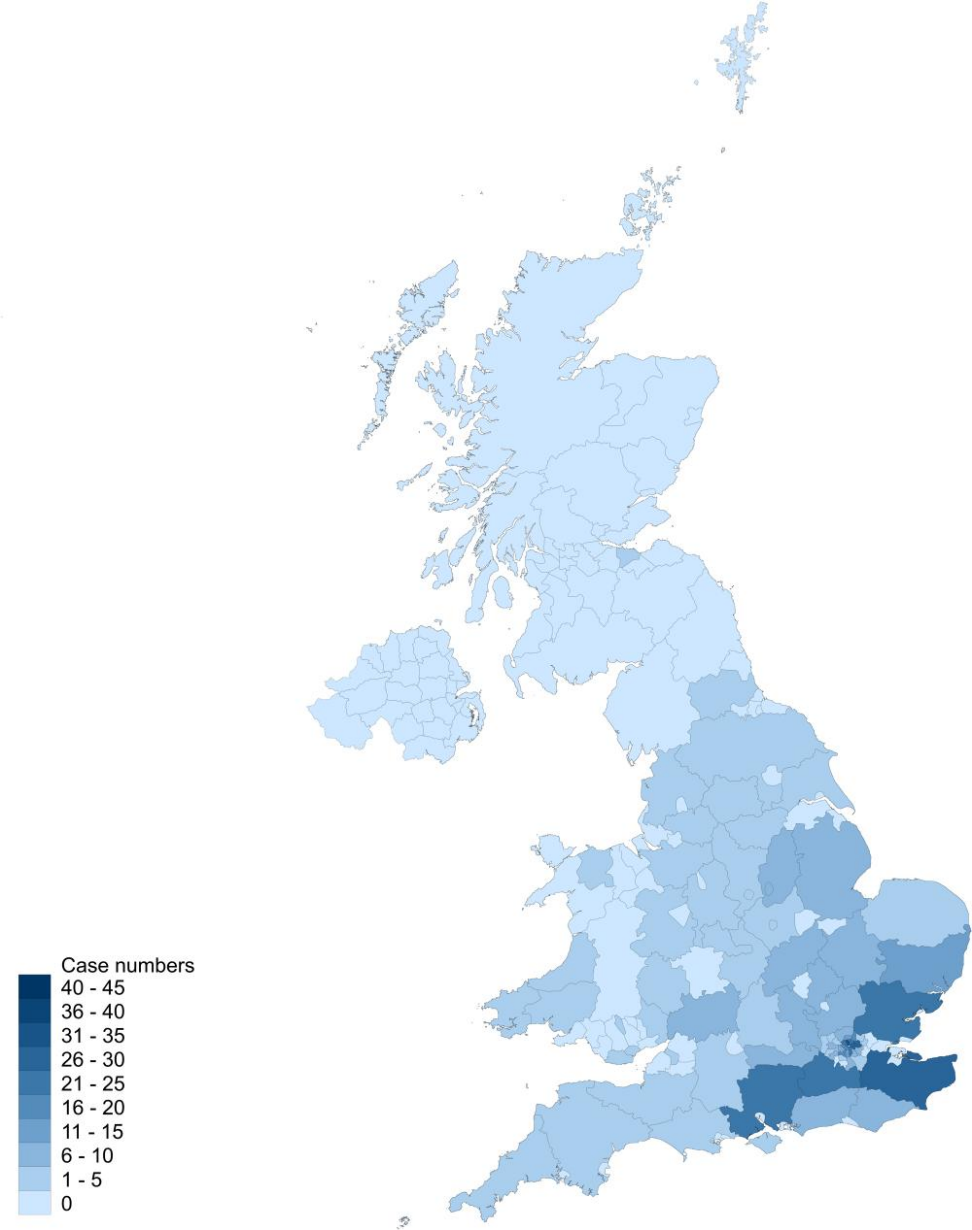
Heat map showing UK geographical distribution of cases.

Sociodemographic variables and details of hospital admissions are summarized in Table 2. Cases were considerably younger than controls but similar in sex ratio, socioeconomic status and admission year. Duration of admission was longer in the cases, but mortality was lower. Dates of illness onset and hospital admission for the cases are shown in Fig. 2. Cases were largely from London and the South-East of England, as shown in Fig. 3. During admission, 15 of the cases died, of whom the cause was attributed to acute encephalitis lethargica in four, pneumonia in two and pulmonary haemorrhage in one, while cause was unclear in eight cases. A post-mortem examination was recorded in eight of the cases.

**Table 2:** Sociodemographic and hospital admission details for included cases

*N* = 679^a^	Cases (*N* = 614)	Controls (*N* = 65)
**Age at admission/years, median (IQR) (*N* = 678)^b^**	29 (18)	41 (29)
**Male sex, *n* (%)**	315 (51.3)	35 (53.9)
**Marital status, *n* (%)**		
Single	323 (52.6)	28 (43.1)
Married	268 (43.7)	33 (50.8)
Widowed	8 (1.3)	2 (3.1)
Not stated	15 (2.4)	2 (3.1)
**Main occupation of household, *n* (%)**		
Armed forces	19 (3.1)	0 (0.0)
Clerical support work	61 (9.9)	9 (13.9)
Craft and related trades worker	116 (18.9)	10 (15.4)
Elementary occupation	62 (10.1)	2 (3.1)
Managers	6 (1.0)	3 (4.6)
Plant and machine operators	102 (16.6)	12 (18.5)
Professionals	61 (9.9)	6 (9.2)
Service and sale work	54 (8.8)	6 (9.2)
Skilled agricultural, forestry and fishery workers	15 (2.4)	2 (3.1)
Technicians and associate professionals	33 (5.4)	1 (1.5)
Unemployed	13 (2.1)	1 (1.5)
Not stated	72 (11.7)	13 (20.0)
**Socioeconomic status, *n* (%)**		
Upper middle class	8 (1.3)	2 (3.1)
Middle class	61 (9.9)	8 (12.3)
Lower middle class	178 (29.0)	17 (26.2)
Skilled working	160 (26.1)	16 (24.6)
Working class	118 (19.2)	8 (12.3)
Non-working	17 (2.8)	1 (1.5)
Not stated	72 (11.7)	13 (20.0)
**Admission year, median (IQR)**	1927 (6)	1929 (9)
**Admission year, min–max**	1918–1946	1918–1940
**Admission duration (days), median (IQR)^b^**	48 (40)	38 (44)
**Consultant, *n* (%)**		
Dr Grainger Stewart	86 (14.0)	4 (6.2)
Dr Kinnier Wilson	83 (13.5)	14 (21.5)
Dr Gordon Holmes	71 (11.6)	12 (18.5)
Dr Risien Russell	68 (11.1)	3 (4.6)
Dr James Collier	66 (10.8)	3 (4.6)
Dr Hinds Howell	63 (10.3)	7 (10.8)
Other	177 (28.8)	22 (33.8)
**Admission outcome, *n* (%)**		
Much improved	20 (3.3)	3 (4.6)
Improved	304 (49.5)	23 (35.4)
Slightly improved	90 (14.7)	7 (10.8)
No change	167 (27.2)	18 (27.7)
Worsened	3 (0.5)	3 (4.6)
Died	15 (2.4)	7 (10.8)
Not stated	5 (0.8)	0 (0)

^a^Statistics shown for full sample of 679 unless otherwise stated.

^b^Violin plots shown in Supplementary Figs 3 and 4.

### Temporality of illness

Year of symptomatic onset ranged between 1903 and 1944 (median 1924, IQR 5), as illustrated in Fig. 2. At admission, the median time since the onset of neurological or neuropsychiatric symptoms was 3.00 years (IQR 3.52) and 34 (5.5%) presented acutely (defined as within 30 days of disease onset). In the 511 cases where it was specified, the median time from onset of neurological or neuropsychiatric symptoms to onset of parkinsonism was 3.9 months (IQR 18.0; see violin plot in Supplementary Fig. 5). Of these patients, 150 (29.4%) had an onset of parkinsonism within 7 days of the onset of neurological or neuropsychiatric symptoms. In terms of distinct illness episodes separated by remission, 488 (79.5%) had only one episode, 122 (19.9%) had two episodes and 4 (0.7%) had three episodes. Onset was considered acute or subacute in 405 (66.0%) of the cases.

### Clinical features

**Figure 4:**
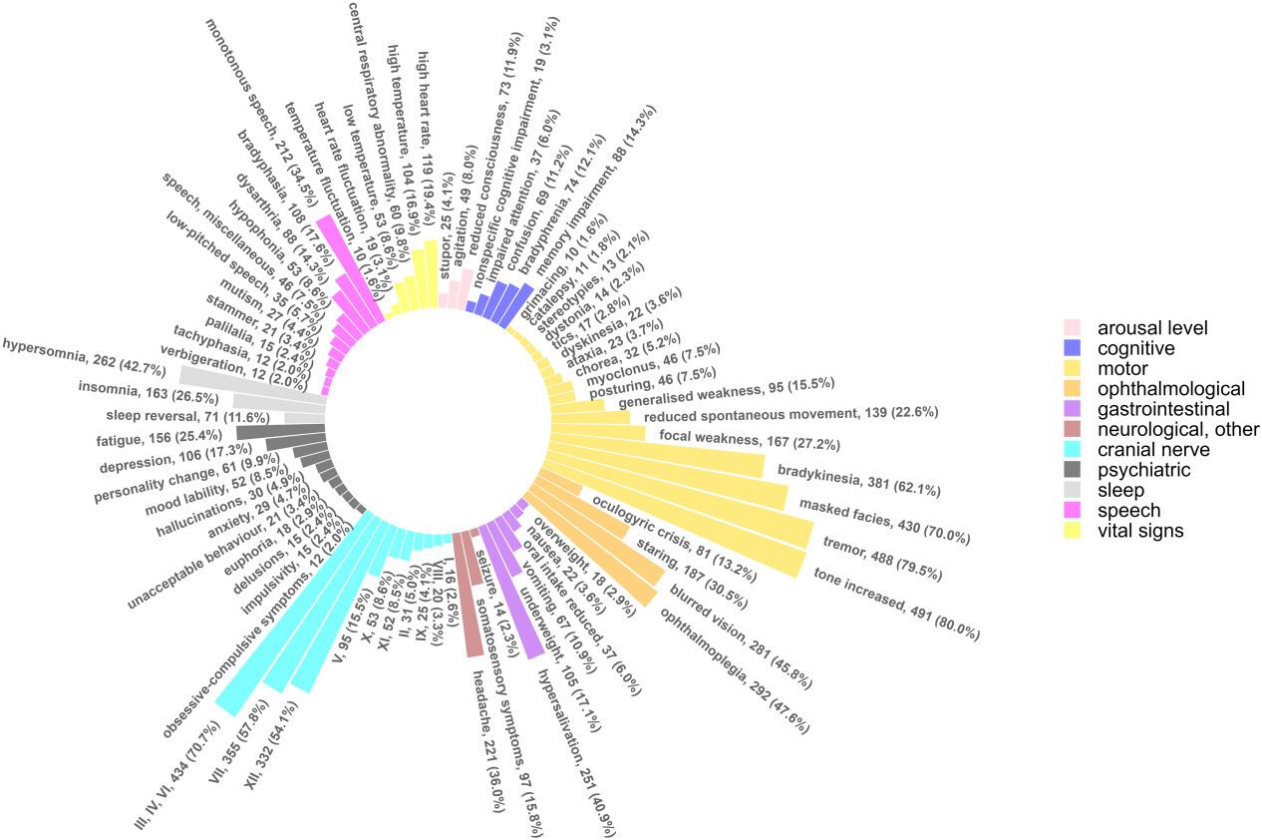
Frequencies of all clinical features occurring in 10 or more individuals.

**Figure 5:**
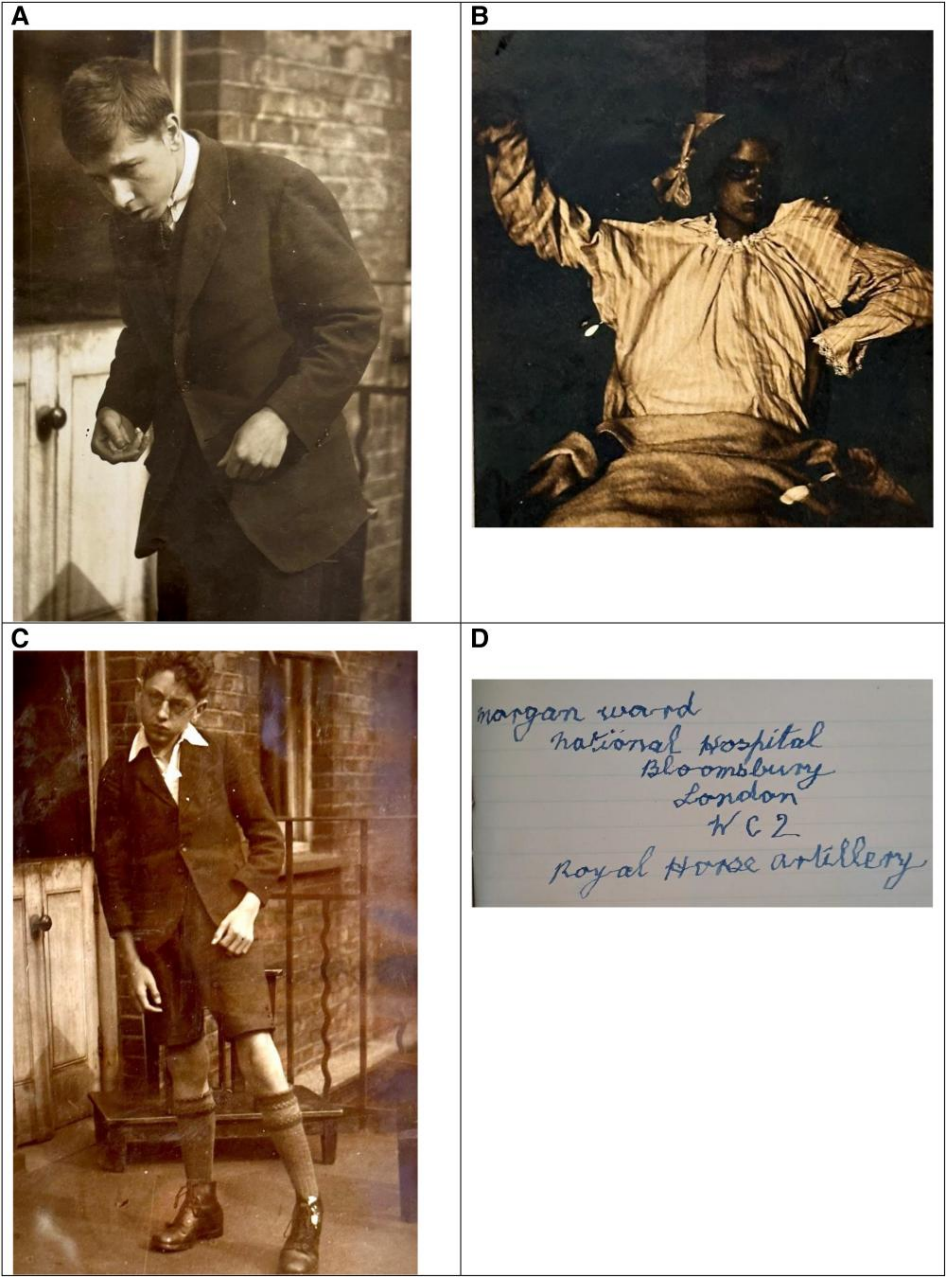
**Photographs of patients with encephalitis lethargica.** (**A**) A 20-year-old man with parkinsonism and bradyphrenia. A metal-worker by trade, one of his early symptoms was finding that he was ‘pulled forward’ when trying to pull a lever. (**B**) A 12-year-old girl in the acute phase of the illness, exhibiting signs of catatonia, including stupor, mutism, staring, waxy flexibility, posturing and catalepsy, her limbs remaining in whichever position they are placed. (**C**) A 14-year-old boy whose symptoms began with a ‘nervous breakdown’ studying for an exam and developed into parkinsonism and a left hemiparesis, illustrating a circumducting gait. (**D**) Tremulous handwriting from a 20-year-old man who first presented 6 years earlier when his employer complained he was falling asleep at work.

Of the 614 cases, 273 (44.5%) had an explicit diagnosis of encephalitis lethargica (e.g. lethargic encephalitis and epidemic encephalitis), while 341 (55.5%) had an implicit diagnosis (e.g. post-encephalitic parkinsonism, post-encephalitic paralysis agitans and post-encephalitis). The categories of clinical features in these explicit and implicit diagnoses are shown in Supplementary Fig. 6 and are extremely similar. The frequencies of all clinical features reported in at least 10 patients are shown in Fig. 4. In addition, during the course of reading through the case notes, we made some additional observations of interesting clinical features that have not hitherto received much attention, which we highlight in Table 3. We suggest the term ‘phonia paradoxa’ for the phenomenon—analogous to kinesia paradoxa—in which individuals’ habitual hypophonic speech was corrected by singing, shouting or public speaking. Some selected photographs taken from the case notes are shown in Fig. 5.

**Table 3:** Miscellaneous observations of clinical features and treatments

Clinical domain	Observations
Motor	Early symptoms: Often the first symptom of the more chronic phase of the illness was unilateral stiffness or a reduced arm swing, as in one patient whose first symptom was that he would travel round in circles when rowing, or another who had difficulty playing the piano. However, musculoskeletal pain was also a very common initial symptom. Insight: Many patients had insight into their parkinsonism, recognizing that they were moving more slowly and that their speech had become quiet and monotonous. One patient with stupor and catalepsy in acute encephalitis lethargica improved and stated she had ‘felt like starch’. Movement during sleep: Tremor was noted in several cases to stop during sleep. However, there was one patient who had abnormal stereotypic ‘piano-playing movements’ during sleep. Kinesia paradoxa: One patient, who usually held himself ‘quite motionless’ and exhibited marked bradykinesia, was able to catch another patient who was falling ‘in the burst of the moment’. Another patient, who was usually unable to stand or undress himself, was once called ‘spindle-legs’ by his father and managed to leap up from his chair to hit him. Several patients were able to run better than walk, while one found it easier to pick up heavy objects than light ones. Bizarre intermittent movements: One patient with ‘fidgeting movements’ would find that each evening, he would fling his limbs about wildly and start running and turning somersaults. Another’s gait was shuffling, but every few minutes would pirouette two or three times.
Oculogyric crises	Precipitants: Emotions—both positive and negative—were cited on several occasions as precipitants and in one patient it was noted that a crisis could be terminated if he could be distracted. One patient’s crises were precipitated by bending down. Psychiatric symptoms during crises: One patient described features of depersonalization with a ‘feeling of unreality’, stating that he knew what to do but did it ‘unconsciously’ and ‘automatically’. Several patients had forced thinking or obsessional thoughts, such as one patient who felt compelled to think of words 7–9 letters long or another who would have to spell out the same word repeatedly. Others described feeling worried or hopeless during the attacks.
Sleep	Hypersomnia: The severity of hypersomnia was very stark in many cases, such as one individual who fell asleep and awoke 10 weeks later and another who was in such deep sleep that she was blistered by a hot water bottle. One patient would fall asleep so easily that she would fall downstairs and on one occasion when she fell asleep in front of the fire was seriously burnt.
Psychiatric	Socially unacceptable behaviour: Several patients had a severe change in their personality and began engaging in behaviours that would previously have been alien to them. Four started engaged in kleptomania and more generally some were noted to have lost the ability to perceive right from wrong. Two patients started making sudden unprovoked attacks, one throwing knives at other patients. Impulse control behaviours: A couple of patients had typical features of obsessive-compulsive disorder. Two were noted to have compulsive nose-picking. One patient found himself unable to stop repetitive behaviours like polishing shoes or beating eggs, despite a desire to. The behaviours were distinctly ego-dystonic. Suicide: Two patients were noted to have died by suicide, though the cause of subsequent death in the period after hospital admission was rarely recorded. In addition, one patient reported suicidal ideation and another had attempted suicide. Difficulty in initiating actions: Several patients were noted to have extreme difficulty in initiating actions, such as one who took ‘a whole day to write a letter, nerving himself up to write one word at a time.’ Affect: While a few patients were noted to have difficulty expressing their emotions despite a wish to do so, uncontrollable emotional expression seemed more common. Several patients were noted to laugh uproariously or break down in tears with very little emotional precipitant.
Speech	‘Phonia paradoxa’: The classic hypophonic, monotonous speech of parkinsonism was extremely common, but in several patients such speech abnormalities were noted to be corrected when the patient sang, shouted or spoke in public.
Other clinical features	Dysphagia: This was a surprisingly common clinical feature and often appeared even in relatively mild cases. Visual distortions: Blurred vision and diplopia were common, but more intriguingly one patient with diplopia in the acute illness saw one of the images upside-down. Another patient in the acute illness could only see half of an object, sometimes the right, sometimes the left.
Treatments	Medical: By far the most common treatments used were anticholinergic medications. Other treatments included parathyroid extract, adrenaline (to regulate the sleep–wake cycle) and even malaria therapy. Surgical: Surgical approaches were occasionally used, including one operation by Sir Percy Sargent to cut some of the right dorsal cervical and thoracic roots to relieve tremor. In another patient, fingers that were in fixed flexion were amputated. Other treatments: One patient underwent X-ray treatment to the parotid gland for hypersalivation. Dr Gordon Holmes conducted daily psychotherapy with a patient for 2 months with the aim of treating her oculogyric crises.

Among the controls, the diagnoses were as follows: neurosyphilis (8 patients, 12%), CNS tumour (6, 9%), multiple sclerosis (6, 9%), primary psychiatric disorder (6, 9%), trigeminal neuralgia (5, 8%), epilepsy (3, 5%), functional neurological disorder (3, 5%), neurasthenia (3, 5%), acute poliomyelitis (2, 3%), CNS infection (2, 3%), Charcot-Marie-Tooth disease (2, 3%), chorea unspecified (2, 3%), meningocoele (2, 3%), Sydenham’s chorea (2, 3%), other specified diagnoses (10, 15%) and undiagnosed (3, 5%).

### Cerebrospinal fluid

One hundred and sixty-seven cases underwent lumbar puncture, of whom 156 (94.0%) had a clear appearance, 7 (4.2%) visible blood and 3 (1.8%) a yellow or turbid appearance. White cells were reported as being present in 31 patients, of whom 20 (12.0% of all lumbar punctures) had a pleocytosis (defined as a white cell count of more than 5 cells per cubic millimetre).^44^ The median protein content (measured in 147 patients) was 0.035 g per 100 millilitres (IQR 0.025). Nonne-Appelt and Pándy tests (markers of raised protein)^48^ were positive in 25/158 (15.8%) and 23/86 (26.7%), respectively. Lange’s test (an indication of neurological syphilis) was not suggestive of syphilis in any of the 130 patients in whom it was performed.

### Validity of diagnostic criteria for encephalitis lethargica

The contingency table comparing cases and controls against the Howard and Lees diagnostic criteria for encephalitis is shown in Supplementary Table 5. Overall, the sensitivity was 28.5% (95% CI 25.0–32.3%) and the specificity was 96.9% (95% CI 89.3–99.6%). The area under the ROC curve was 0.63 (95% CI 0.59–0.66%). The positive likelihood ratio was 9.26 and the negative likelihood ratio was 0.74, giving a diagnostic odds ratio of 12.6. The sensitivity, specificity and area under the ROC curve for the individual items of the Howard and Lees criteria are shown in Supplementary Table 6.

### Assessment of aetiological theories

#### Influenza, febrile illnesses and contagion

Of the 614 cases, 226 (36.8%) had a documented episode of influenza, of whom 134 (21.8%) had an episode in the calendar year of the illness onset, or the previous calendar year. It was often not clear whether a febrile illness was influenza or not. Including all episodes of febrile illness within one calendar year of the illness onset, there were 195 cases (31.8%, 95% CI 28.1–35.6%). This compared to eight (12%, 95% CI 5–23%) of the controls, corresponding to an odds ratio (OR) in Model 1 of 2.70 (95% CI 1.02–7.20, *P* = 0.05) and OR in Model 2 of 2.43 (95% CI 0.90–6.55, *P* = 0.08). However, the absence of influenza or another febrile illness was rarely stated. Only five (0.8%) cases had a definite family history of encephalitis, while three (0.5%) had a possible family history.

#### Environmental exposures

Among the cases, 526 (85.7%) had an urban address, compared to 58 (89.2%) among the controls. In logistic regression, the Model 1 OR was 1.07 (95% CI 0.39–2.92, *P* = 0.90) and the Model 2 OR was 1.10 (95% CI 0.39–3.08, *P* = 0.86). Limiting the analysis of occupational exposures to the 369 cases and 35 controls whose own occupations were listed, 106 (28.7%) of the cases had likely solvent exposure, compared to 9 (25.7%) of the controls. The Model 1 OR was 1.00 (95% CI 0.44–2.27, *P* = 0.95), and the Model 2 OR was 0.86 (95% CI 0.37–1.97, *P* = 0.71). In terms of heavy metals, 71 (19.2%) of the cases had likely exposure, compared to 9 (25.7%) of the controls, giving a Model 1 OR of 0.69 (95% CI 0.30–1.60, *P* = 0.39) and Model 2 OR of 0.58 (95% CI 0.24–1.38, *P* = 0.22).

#### Autoimmune encephalitis

In terms of the Graus criteria for possible autoimmune encephalitis (Table 1), 278 (45.3%) of the cases met criterion 1 (subacute onset of working memory deficits, altered mental status or psychiatric symptoms) and 611 (99.5%) met criterion 2 (new focal CNS findings, seizures not explained by a previously known seizure disorder or CSF pleocytosis). Given the absence of modern investigations, it was not possible to establish reasonable exclusion of alternative causes, but a total of 276 (45.0%) met criteria 1 and 2 for possible autoimmune encephalitis.

In terms of the criteria for probable NMDA receptor encephalitis (Table 1), 31 (5.1%) of cases met criterion 1 (rapid onset of at least four of abnormal behaviour or cognitive dysfunction, speech dysfunction, seizures, movement disorder, decreased level of consciousness, autonomic dysfunction or central hypoventilation) and 20 (3.3%) met criterion 2 (CSF pleocytosis). As previously, it was not possible to establish exclusion of alternative causes, but three (0.5%) cases met criteria 1 and 2 for probable NMDA receptor encephalitis. Only two cases (0.3%) had a diagnosis of a neoplastic disease, of which both were brain tumours. The cases were largely very dissimilar to NMDA receptor encephalitis.

#### Catatonia

Among the cases, 14 (2.3%) exhibited three or more of the DSM-5-TR features of catatonia at any time in their illness, compared to none of the controls. Of these 14 patients, 11 (1.8%) exhibited three or more signs at the same point in time, reaching the DSM-5-TR definition of catatonia.

## Discussion

### Summary of findings

In this study of 614 cases of encephalitis lethargica admitted to a neurological hospital between 1918 and 1946, there had been a median of 3.00 years since the onset of symptoms. The sociodemographic features of our cases are consistent with the contemporaneous literature, which found a peak among young adults, a roughly equal sex ratio and a spread across socioeconomic groups.^8^ The median time from disease onset to parkinsonism was approximately four months, but there was a wide range, which fits with other reports of an interval of between a few months to several years.^2,18^ The most common clinical features were motor, but cranial nerve findings, ophthalmological features, sleep abnormalities, gastrointestinal or nutritional features, speech disorders and psychiatric features were also very common. In those who underwent lumbar puncture, the vast majority had a clear appearance to their CSF with only 12.0% having a pleocytosis.

The Howard and Lees criteria for encephalitis lethargica applied retrospectively were highly specific (96.9%, 95% CI 89.3–99.6) but relatively insensitive (28.5%, 95% CI 25.0–32.3%). Febrile illnesses were more possibly likely to be associated with a case of encephalitis lethargica than with a control condition (aOR 2.70, 95% CI 1.02–7.20, *P* = 0.05), but only 31.8% had such an illness among the cases within one calendar year prior to illness onset and only five had a definite family history of encephalitis. There was little evidence that patients with encephalitis lethargica may have had occupational exposure to solvents or heavy metals. Only 11 (1.8%) of the cases convincingly met the DSM-5-TR criteria for catatonia. Almost half the cases might meet criteria for possible autoimmune encephalitis, although only 0.5% might meet criteria for probable NMDA receptor encephalitis.

### Strengths and limitations

This study is original in the study of encephalitis lethargica, as it uses retrospective case data from the time of the epidemic but applies modern epidemiological methods. The number of cases is also much larger than other studies published in recent decades. The control group is taken from the same population as the cases and constitutes a broad range of alternative diagnoses.

However, the study does have limitations in its generalizability, selection, measurement, missing data, unmeasured confounding and wide effect size measurements.

In terms of the descriptive statistics, our study is drawn from one hospital that specialized in the study of neurological disorders, which might not be truly representative, although it is reassuring that patients were admitted from many parts of Great Britain (Fig. 3). What is probably a greater issue is the timing of the study. The start date of 1918 is consistent with this being the year of the first outbreak in England,^10^ but the end date of 1946 meant that there were relatively few cases with the extremely advanced parkinsonism described elsewhere in the later literature.^49^ We also expect that the results may be affected by survival bias, given that 20–40% of individuals died during the acute illness,^9,13,50^ such individuals having less opportunity to be admitted to a specialist hospital. Our assumption that no mention in the notes of a clinical feature indicates it was not present is commonly applied in retrospective studies, but this runs some risk of misclassification bias. This is likely to have led to the underreporting of some clinical features, particularly those for which there was not consistently systematic enquiry or examination, such as psychiatric symptoms. It may also have led to an underrepresentation of symptoms occurring during the acute illness. It is also possible that there was overdiagnosis of encephalitis lethargica during the epidemic period.^51,52^ If this is the case, it is likely to have introduced a bias towards the null hypothesis in the diagnostic accuracy, such that we may have underestimated the sensitivity and specificity of the Howard and Lees criteria.

Some additional limitations must be considered with regard to the analyses of diagnostic criteria and aetiology. A larger sample size would have provided more precise estimates. Hospital controls have been criticized based on the assumption that they are representative of the exposure rate in the background population.^53^ However, given the diversity of neurological diagnoses in the control group, it is unlikely that this had a large effect. Moreover, hospital controls in this study had the advantage of representing the geographical and occupational diversity of the cases in a way that healthy local residents would not. However, the impact of unmentioned clinical features is likely—in at least some variables—to have been highly differential between cases and controls. In particular, a relationship between influenza and encephalitis lethargica was an early hypothesis,^54-56^ albeit one that was heavily disputed,^12,57,58^ so it is not surprising that influenza, which was a clinical diagnosis, was mentioned as being present and absent more among the cases than the controls. There is also the issue that it is hard to distinguish influenza from other febrile illnesses clinically, especially when the infection may have occurred some years before hospital admission. Finally, the moderately wide confidence intervals do not rule out any result of epidemiological relevance, but they do not point to a single environmental cause for encephalitis lethargica.

### Interpretation of findings

Our study demonstrates that encephalitis lethargica had a wide range of clinical features, which varied over time but usually culminated in parkinsonism. While there were undoubtedly misdiagnoses of encephalitis lethargica,^59^ and it has been asserted that encephalitis lethargica was a ‘heterogeneous group of conditions’,^2^ the current work has shown that there were clinical features that were consistently identified and clinical criteria can be applied that essentially exclude non-cases. There were several descriptions of paradoxical movement and speech. Kinesia paradoxa, first coined with reference to Parkinson’s disease in 1921,^60^ was soon used with reference to encephalitis lethargica, describing patients who despite their habitual bradykinesia were sometimes able to move quickly, dance or even perform gymnastics.^61,62^ The ability consciously to increase speech volume or to amplify the voice in speech is reported in Parkinson’s disease,^63,64^ but—to our knowledge—has not been described in encephalitis lethargica.

Given the centrality of parkinsonism, it is worth asking whether a monogenic young-onset Parkinson’s disease could explain a large proportion of the cases, but this is inconsistent with the abnormal findings on examination of the extraocular motor nerves in 70.7%, profound sleep abnormalities in a large number and the much larger number of cases of encephalitis lethargica during these years compared to cases of Parkinson’s.

Febrile illnesses were a common prodrome of encephalitis lethargica, and likely much more common than in neurological controls. However, given that only 36.8% of encephalitis lethargica cases ever had influenza recorded, it seems implausible that influenza was the single cause. Moreover, the extremely low contagion in our study weakens this hypothesis further. We did not find any evidence to support the role of an environmental toxin, although we were limited to exposures that might be due to occupation.

While the cases of catatonia in our study were striking, they accounted for a very small proportion, refuting the suggestion that encephalitis lethargica is simply a form of catatonia. Our finding that almost half the cases may meet criteria for possible autoimmune encephalitis but only 0.5% for NMDA receptor encephalitis suggest that encephalitis lethargica might have been a different form of autoimmune encephalitis. There is, however, concern that careless implementation of criteria for possible autoimmune encephalitis without use of magnetic resonance imaging or exclusion of alternative diagnoses can result in spurious diagnoses,^65,66^ potentially missing neoplastic, infectious, genetic, neurodegenerative, primary psychiatric and functional neurological disorders.^67,68^ The proportion who may meet criteria should therefore be regarded as an upper bound. How this fits with the findings from Dale *et al.*^28^ of NMDA receptor antibodies in the serum of half their recent supposed encephalitis lethargica patients is probably explained by the patients’ phenotypes. Among Dale and colleagues’ antibody-positive paediatric patients, 90% had agitation (compared to 8.0% in the current study), 30% had catatonia (1.8%), 70% had mutism (4.4%), 100% had dyskinesias (3.6%), 0% had parkinsonism (85.0%) and 50% had seizures (2.3%). Notwithstanding some overlap in the insomnia and autonomic dysfunction between the two groups, it is clear that these are very different groups of patients. We therefore refute the suggestion that encephalitis lethargica is purely unrecognized NMDA receptor encephalitis, a condition that does not commonly feature parkinsonism. However, despite its recent description,^69^ it seems highly plausible that there were many historical cases that were diagnosed differently at the time.^70^ It is therefore possible that—were a case of NMDA receptor encephalitis to have developed in the 1920s or 1930s—the individual would have been misdiagnosed with encephalitis lethargica.

## Conclusion

Our study, which reports a large number of cases from the epidemic period, supports the idea that encephalitis lethargica is a distinct neuropsychiatric entity. We have found that a modern case definition (Howard and Lees) shows good reliability and validity and may be applied retrospectively to historical case records. In terms of aetiology, we have been able to cast serious doubt on various hypotheses, specifically those relying on environmental exposures preferentially related to urban or rural settings, occupational solvent exposure and occupational heavy metal exposure. Influenza, though likely present in a large proportion of cases, seems to have been neither necessary nor sufficient for the genesis of encephalitis lethargica. A large proportion of cases may have met modern criteria for autoimmune encephalitis and this remains a plausible aetiological theory, although typical cases of encephalitis lethargica are dissimilar to contemporary NMDA receptor encephalitis.

## Supplementary Material

fcae347_Supplementary_Data

## Data Availability

The ‘Stata’ code is available in Supplementary Table 3. The R code is available in Supplementary Table 4. The original data are freely available for download from OSF at https://osf.io/6nafj/? view_only=507840bd2b624beb8dc672f301bdd60c.
