# Supplementary material for: Encephalitis lethargica: clinical features and aetiology
Source: Brain Commun. 2024 Oct 4;6(5):fcae347. doi: 10.1093/braincomms/fcae347 (PMC11495101; doi:10.1093/braincomms/fcae347)
Supplement: fcae347_Supplementary_Data [file fcae347_supplementary_data.docx]

Encephalitis lethargica – a contemporary re-analysis

Supplementary material

Contents

[Supplementary Table 1: STROBE checklist 2](#_Toc166761302)

[Supplementary Table 2: definition and derivation of variables in extraction database 3](#_Toc166761303)

[Supplementary Table 3: Stata code 12](#_Toc166761304)

[000 Master do-file.do 12](#_Toc166761305)

[001 Data cleaning.do 12](#_Toc166761306)

[002 Create secondary variables.do 22](#_Toc166761307)

[003 Eligibility.do 26](#_Toc166761308)

[004 Basic descriptive stats.do 27](#_Toc166761309)

[006 Correspondence to diagnostic criteria.do 31](#_Toc166761310)

[007 Influenza and febrile illnesses.do 34](#_Toc166761311)

[008 Geographical distribution.do 34](#_Toc166761312)

[011 Validation of Lees criteria.do 36](#_Toc166761313)

[013 Interrater reliability.do 38](#_Toc166761314)

[014 CSF.do 43](#_Toc166761315)

[Supplementary Table 4: R code 44](#_Toc166761316)

[Supplementary Table 5: Contingency table for Howard and Lees criteria for encephalitis lethargica 49](#_Toc166761317)

[Supplementary Table 6: Performance of individual items of the Howard and Lees criteria for encephalitis lethargica 50](#_Toc166761318)

[Supplementary Figure 1: Example case notes from the first eligible patient admitted 51](#_Toc166761319)

[Supplementary Figure 2: Example case notes from the last eligible patient admitted 52](#_Toc166761320)

[Supplementary Figure 3: Violin plot of age at admission 53](#_Toc166761321)

[Supplementary Figure 4: Violin plot of admission duration 54](#_Toc166761322)

[Supplementary Figure 5: Violin plot of time from onset of neurological or neuropsychiatric symptoms to onset of parkinsonism 55](#_Toc166761323)

[Supplementary Figure 6: Frequencies of categories of clinical features where diagnosis of encephalitis lethargica was implicit and explicit 56](#_Toc166761324)

[Supplementary References 56](#_Toc166761325)

# Supplementary Table 1: STROBE checklist

|  | Item No | Recommendation | Page No |
| --- | --- | --- | --- |
| **Title and abstract** | 1 | (*a*) Indicate the study’s design with a commonly used term in the title or the abstract | 1 |
|  |  | (*b*) Provide in the abstract an informative and balanced summary of what was done and what was found | 1 |
| Introduction | | | |
| Background/rationale | 2 | Explain the scientific background and rationale for the investigation being reported | 2-5 |
| Objectives | 3 | State specific objectives, including any prespecified hypotheses | 5 |
| Methods | | | |
| Study design | 4 | Present key elements of study design early in the paper | 6 |
| Setting | 5 | Describe the setting, locations, and relevant dates, including periods of recruitment, exposure, follow-up, and data collection | 6 |
| Participants | 6 | (*a*) Give the eligibility criteria, and the sources and methods of case ascertainment and control selection. Give the rationale for the choice of cases and controls | 6 |
|  |  | (*b*) For matched studies, give matching criteria and the number of controls per case | 6 |
| Variables | 7 | Clearly define all outcomes, exposures, predictors, potential confounders, and effect modifiers. Give diagnostic criteria, if applicable | Supplementary Table 1 |
| Data sources/ measurement | 8* | For each variable of interest, give sources of data and details of methods of assessment (measurement). Describe comparability of assessment methods if there is more than one group | Supplementary Table 1; 7 |
| Bias | 9 | Describe any efforts to address potential sources of bias | 7; 9 |
| Study size | 10 | Explain how the study size was arrived at | 6 |
| Quantitative variables | 11 | Explain how quantitative variables were handled in the analyses. If applicable, describe which groupings were chosen and why | Supplementary Table 1; 7; 9 |
| Statistical methods | 12 | (*a*) Describe all statistical methods, including those used to control for confounding | 7; 9 |
|  |  | (*b*) Describe any methods used to examine subgroups and interactions | N/A |
|  |  | (*c*) Explain how missing data were addressed | 7 |
|  |  | (*d*) If applicable, explain how matching of cases and controls was addressed | 6-7; 9 |
|  |  | (*e*) Describe any sensitivity analyses | N/A |
| Results | | | |
| Participants | 13* | (a) Report numbers of individuals at each stage of study—eg numbers potentially eligible, examined for eligibility, confirmed eligible, included in the study, completing follow-up, and analysed | Figure 1 |
|  |  | (b) Give reasons for non-participation at each stage | Figure 1 |
|  |  | (c) Consider use of a flow diagram | Figure 1 |
| Descriptive data | 14* | (a) Give characteristics of study participants (eg demographic, clinical, social) and information on exposures and potential confounders | 10-11 |
|  |  | (b) Indicate number of participants with missing data for each variable of interest | 10-11 |
| Outcome data | 15* | Report numbers in each exposure category, or summary measures of exposure | 16-17 |

# Supplementary Table 2: definition and derivation of variables in extraction database

| Variable category | Variable | Source | Definition | Pre-processing |
| --- | --- | --- | --- | --- |
|  |  |  |  |  |
| Demographic | Year | Structured field | Year of discharge |  |
|  | Age | Structured field | Age in years at time of admission |  |
|  | Sex | Free text | Inferred based on name and pronouns used |  |
|  | Address | Structured field | Patient’s home address | County extracted separately. Coded as urban or rural on the basis that city and town addresses are urban, while villages and the countryside are rural. |
|  | Marital status | Structured field | Patient’s marital status at time of admission |  |
|  | Occupation | Structured field | Occupation of the patient or a close relative (either the father, mother or husband) | Occupation classified by whether it belongs to the patient or their relative. Occupational types classified according to the major groups of the International Standard Classification of Occupations 8 (ISCO-08). ^1^ Socioeconomic status was derived based on the National Statistics Socio-Economic Classification (NS-SEC). ^2^ Exposure to solvents* and heavy metals † were derived based on high-risk occupational groups. |
|  | Handedness | Free text | Whether patient is left-handed, right-handed or ambidextrous |  |
| Admission details | Admission date | Structured field | Date of index admission to hospital |  |
|  | Discharge date | Structured field | Date of discharge from current admission or death |  |
|  | Outcome | Structured field | Global impression of patient outcome in terms of status relative to their admission. | Grouped as the following categories: died, worsened, no change, slightly improved, improved, much improved and recovered. |
|  | Cause of death | Free text | Inferred from clinical notes and postmortem examination (if performed) |  |
|  | Presenting complaint | Structured field | Reason for presentation to hospital |  |
| Disease timing | Duration of illness | Free text | Time from start of neurological or psychiatric symptoms until hospital admission in days. |  |
|  | Neurological episodes | Free text | Number of distinct episodes of neurological illness separated by remission |  |
|  | Time from neurological onset to parkinsonism | Free text | Time from first neurological symptoms to onset of parkinsonism |  |
|  | Acute or subacute onset | Free text | 1 if the illness arises suddenly (e.g. an acute encephalitic illness) or there is rapid progression over < 3 months. 0 if there is a gradual onset of symptoms that probably occurred over ≥ 3 months. |  |
| Past medical history | Influenza | Free text | 1 if there is any episode of influenza mentioned in the 10 years prior to the onset of symptoms |  |
|  | Influenza date 1 - 8 | Free text | 8 variables indicating the years of any episodes of influenza in the previous 10 years |  |
|  | Febrile illness | Free text | 1 if there was any febrile illness temporally related to the symptomatic onset that was not stated to be influenza; otherwise, 0. |  |
|  | Febrile illness date | Free text | Year of any such febrile illness |  |
|  | Neoplasia | Free text | 1 if there was any neoplastic disease; otherwise, 0. |  |
|  | Neoplasia type | Free text | Type of any such neoplastic disease |  |
| Family history | Family history of encephalitis | Free text | 1 if there was any family history of encephalitis of any form; otherwise, 0. |  |
| Level of arousal | Reduced consciousness | Free text | ‡ |  |
|  | Stupor | Free text | According to DSM-5-TR catatonia definition: absence of psychomotor activity; not actively responding to the environment. ‡ |  |
|  | Agitation | Free text | Agitation that was not influenced by external stimuli. ‡ |  |
| Gastrointestinal / nutritional features | Nausea | Free text | ‡ |  |
|  | Vomiting | Free text | ‡ |  |
|  | Hypersalivation | Free text | Includes dribbling during the examination. ‡ |  |
|  | Overweight | Free text | ‡ |  |
|  | Underweight | Free text | ‡ |  |
|  | Increased appetite | Free text | ‡ |  |
|  | Reduced oral intake | Free text | ‡ |  |
|  | Polydipsia | Free text | ‡ |  |
| Sleep disorders | Sleep-wake cycle reversal | Free text | ‡ |  |
|  | Insomnia | Free text | Coded as 0 if insomnia is present only in the context of sleep-wake cycle reversal. ‡ |  |
|  | Hypersomnia | Free text | Coded as 0 if hypersomnia is present only in the context of sleep-wake cycle reversal. ‡ |  |
|  | Parasomnia | Free text | ‡ |  |
| Motor features | Tremor | Free text | Of any type, e.g. resting, postural, kinetic. ‡ |  |
|  | Tone increased | Free text | Includes rigidity and spasticity (which were often not differentiated). ‡ |  |
|  | Bradykinesia | Free text | Includes an objective examination finding or a subjective description of motor slowing. Does not include less precise descriptions of slowing (e.g. ‘slow at work’), which might indicate either bradykinesia or bradyphrenia. ‡ |  |
|  | Tics | Free text | ‡ |  |
|  | Chorea | Free text | ‡ |  |
|  | Myoclonus | Free text | ‡ |  |
|  | Ataxia | Free text | ‡ |  |
|  | Generalised weakness | Free text | As elicited on examination. ‡ |  |
|  | Focal weakness | Free text | As elicited on examination. ‡ |  |
|  | Masked facies | Free text | Includes parkinsonian facies and lack of emotional expression. ‡ |  |
|  | Reduction in spontaneous movement | Free text | ‡ |  |
|  | Akinetic mutism | Free text | Absence (or almost complete absence) of speech and voluntary movement. ‡ |  |
|  | Catalepsy | Free text | According to DSM-5-TR catatonia definition: passive induction of a posture held against gravity. ‡ |  |
|  | Waxy flexibility | Free text | According to DSM-5-TR catatonia definition: slight and even resistance to positioning by examiner. ‡ |  |
|  | Posturing | Free text | According to DSM-5-TR catatonia definition: spontaneous and active maintenance of a posture against gravity. ‡ |  |
|  | Mannerisms | Free text | According to DSM-5-TR catatonia definition: odd caricature of normal actions. ‡ |  |
|  | Stereotypies | Free text | According to DSM-5-TR catatonia definition: repetitive, abnormally frequent, non-goal-directed movements. ‡ |  |
|  | Grimacing | Free text | ‡ |  |
|  | Echopraxia | Free text | According to DSM-5-TR catatonia definition: mimicking another person’s movements. ‡ |  |
|  | Dyskinesia | Free text | ‡ |  |
|  | Dystonia | Free text | ‡ |  |
| Psychiatric features | Euphoria | Free text | ‡ |  |
|  | Depression | Free text | ‡ |  |
|  | Emotional lability | Free text | Includes affective incontinence and forced laughter or crying. ‡ |  |
|  | Negativism | Free text | According to DSM-5-TR catatonia definition: opposing or not responding to instructions or external stimuli. ‡ |  |
|  | Fatigue | Free text | ‡ |  |
|  | Impulsivity | Free text | ‡ |  |
|  | Socially unacceptable behaviour | Free text | Includes compulsive lying and criminal behaviour. ‡ |  |
|  | Hypersexuality | Free text | ‡ |  |
|  | Obsessive-compulsive behaviour | Free text | Including during oculogyric crises. ‡ |  |
|  | Self-harming | Free text | ‡ |  |
|  | Personality change | Free text | Excludes other psychiatric categories. |  |
|  | Hallucinations | Free text | ‡ |  |
|  | Delusions | Free text | ‡ |  |
|  | Anxiety | Free text | ‡ |  |
|  | Apathy | Free text | ‡ |  |
|  | Depersonalisation / derealisation | Free text | ‡ |  |
|  | Affective blunting | Free text | ‡ |  |
|  | Dissociation | Free text | ‡ |  |
|  | Reduced libido | Free text | ‡ |  |
|  | Miscellaneous psychiatric features | Free text | ‡ |  |
| Cognitive features | Confusion | Free text | ‡ |  |
|  | Memory impairment | Free text | Involving working memory or long-term memory. ‡ |  |
|  | Bradyphrenia | Free text | ‡ |  |
|  | Disorientation | Free text | ‡ |  |
|  | Dysphasia | Free text | ‡ |  |
|  | Anomia | Free text | ‡ |  |
|  | Impaired attention | Free text | ‡ |  |
|  | Impaired numeric function | Free text | ‡ |  |
|  | Perseveration | Free text | ‡ |  |
|  | Right-left disorientation | Free text | ‡ |  |
|  | Subjective cognitive impairment | Free text | ‡ |  |
|  | Cognitive impairment not otherwise specified | Free text | ‡ |  |
| Speech features | Mutism | Free text | Complete or almost complete absence of speech. ‡ |  |
|  | Oligophasia | Free text | Reduction in quantity of speech not to the extent of mutism. ‡ |  |
|  | Echolalia | Free text | According to DSM-5-TR catatonia definition: mimicking another person’s speech. ‡ |  |
|  | Pressure of speech | Free text | ‡ |  |
|  | Palilalia | Free text | Repetition of syllables or single words. ‡ |  |
|  | Verbigeration | Free text | Repetition of entire phrases. ‡ |  |
|  | Bradyphasia | Free text | Slow speech. ‡ |  |
|  | Tachyphasia | Free text | Fast speech. ‡ |  |
|  | Hypophonia | Free text | Quiet speech. ‡ |  |
|  | Monotonous speech | Free text | ‡ |  |
|  | Dysarthric speech | Free text | Includes indistinct speech. ‡ |  |
|  | High-pitched speech | Free text | ‡ |  |
|  | Low-pitched speech | Free text | ‡ |  |
|  | Stammer | Free text | ‡ |  |
|  | Very deliberate speech | Free text | ‡ |  |
|  | Miscellaneous speech abnormality | Free text | ‡ |  |
| Cranial nerve findings | Cranial nerve I | Free text | ‡§ |  |
|  | Cranial nerve II | Free text | ‡§ |  |
|  | Cranial nerves III, IV or VI | Free text | ‡§ |  |
|  | Cranial nerve V | Free text | ‡§ |  |
|  | Cranial nerve VII | Free text | Includes asymmetry or weakness of voluntary movement in the distribution of the facial nerve, but excludes masked facies. ‡§ |  |
|  | Cranial nerve VIII | Free text | ‡§ |  |
|  | Cranial nerve IX | Free text | ‡§ |  |
|  | Cranial nerve X | Free text | Excludes higher level speech abnormalities, such as slow or monotonous speech. ‡§ |  |
|  | Cranial nerve XI | Free text | ‡§ |  |
|  | Cranial nerve XII | Free text | ‡§ |  |
| Ophthalmological features | Ophthalmoplegia | Free text | Paralysis of the eye muscles, including a failure of convergence. ‡ |  |
|  | Oculogyric crisis | Free text | ‡ |  |
|  | Blurred or double vision | Free text | ‡ |  |
|  | Staring | Free text | ‡ |  |
| Abnormalities of physical observations | High temperature | Observation chart | Any temperature > 99⁰F within first 5 days of admission. ‡ |  |
|  | Low temperature | Observation chart | Any temperature < 97⁰F within first 5 days of admission. ‡ |  |
|  | Fluctuating temperature | Observation chart | Both high and low temperature within 24 hours in the first 5 days of admission. ‡ |  |
|  | High heart rate | Observation chart | If age ≥ 15 years, pulse ≥ 100 / minute within the first 5 days of admission. If age < 15 years, refer to Advanced Paediatric Life Support normal values. ^3^ ‡ |  |
|  | Low heart rate | Observation chart | If age ≥ 15 years, pulse < 60 / minute within the first 5 days of admission. If age < 15 years, refer to Advanced Paediatric Life Support normal values. ^3^ ‡ |  |
|  | Fluctuating heart rate | Observation chart | Change of 30 bpm within 24 hours in the first 5 days of admission. ‡ |  |
|  | Central hypoventilation | Free text and observation chart | ‡ |  |
|  | Other central respiratory irregularities | Free text | ‡ |  |
| CSF results | CSF results available | Free text | 1 if CSF results are available; otherwise, 0. |  |
|  | CSF protein | Free text | In grams per 100 millilitres. |  |
|  | CSF albumin | Free text | In grams per 100 millilitres |  |
|  | CSF cell count | Free text | As number per cubic centimetre. | Pleocytosis defined as a white cell count of more than 5 cells per cubic millimetre. ^4^ |
|  | CSF cell type(s) | Free text | Predominant cell type(s) |  |
|  | Nonne-Appelt test | Free text | Archaic test where a positive result indicated raised CSF protein, but its sensitivity and specificity were imperfect. ^5^ |  |
|  | Pándy test | Free text | Archaic test where a positive result indicated raised CSF protein, but its sensitivity and specificity were imperfect. ^5^ |  |
|  | Lange’s test | Free text | Archaic test for neurological syphilis. Results given as a series of numbers representing the amount of colloidal gold precipitation in serial CSF dilutions. ^6^ | Results interpreted as negative, mid-zone pattern, paretic or indeterminate. ^6^ |
|  | Wassermann reaction | Free text | Archaic test for syphilis. ^7^ |  |
| Other neurological features | Headache | Free text | ‡ |  |
|  | Somatosensory symptoms | Free text | Symptoms only (e.g. numbness, tingling), not signs. Does not include pain. ‡ |  |
|  | Seizure | Free text | ‡ |  |
|  | Seizure otherwise unexplained | Free text | Seizure where there is no evident explanation other than encephalitis lethargica. ‡ |  |

bpm – beats per minute. CSF – cerebrospinal fluid.

*Occupations considered as high-risk for solvent exposure: laboratory workers, mechanics, workers in printing shops, workers mixing solvents, painters, dry cleaners, metalworkers, metal degreasers, workers in the oil or chemical industries, artists, cosmetologists and beauticians.

†Occupations considered as high-risk for heavy metal exposure: construction or home renovation workers, miners, firing range employees or those who do target shooting, smelters, stained glass makers, battery or electronic workers, welders, artists, worker conducting grinding, refinishing and repair work, and workers in forges, foundries, refineries, power plants, nuclear power stations, plastic manufacturing, wood preservation and paper processing.

‡Clinical features were coded as binary variables with 1 indicating that the feature was noted to be present at any point in the disease course. Otherwise, these variables were 0.

§Any neurological abnormalities elicited on examination of the specified cranial nerve are coded here. These may or may not actually be due to a cranial nerve palsy, as the aetiology was often unclear.

# Supplementary Table 3: Stata code

## 000 Master do-file.do

//0. Setup

version 17

global ROOT "G:\My Drive\Research\Encephalitis lethargica QS series\Stata\"

set logtype text, permanently

ssc install statplot

ssc install spmap

ssc install shp2dta

ssc install vioplot

ssc install heatplot

ssc install palettes, replace

ssc install colrspace, replace

ssc install venndiag

//1. Call variable-modifying do-files

do "${ROOT}003.2 Do files v2\001 Data cleaning.do"

do "${ROOT}003.2 Do files v2\002 Create secondary variables.do"

do "${ROOT}003.2 Do files v2\003 Eligibility.do"

save "${ROOT}002 DTA files\XXX Ready for analysis.dta", replace

//2. Call analysis do-files

do "${ROOT}003.2 Do files v2\004 Basic descriptive stats.do"

do "${ROOT}003.2 Do files v2\005 Clinical features.do"

do "${ROOT}003.2 Do files v2\006 Correspondence to diagnostic criteria.do"

do "${ROOT}003.2 Do files v2\007 Influenza and febrile illnesses.do"

do "${ROOT}003.2 Do files v2\008 Geographical distribution.do"

do "${ROOT}003.2 Do files v2\010 Evolution of clinical features.do"

do "${ROOT}003.2 Do files v2\011 Validation of Lees criteria.do"

do "${ROOT}003.2 Do files v2\013 Interrater reliability.do"

do "${ROOT}003.2 Do files v2\014 CSF.do"

## 001 Data cleaning.do

log using "${ROOT}004 Log files\001 Data cleaning.log", replace

//Import Excel spreadsheet

clear

import excel "${ROOT}001 Raw data\001 Raw data extraction.xlsx", sheet("Sheet1") firstrow case(lower)

save "${ROOT}002 DTA files\001 Imported data.dta", replace

//Drop unhelpful variables

drop diagnosisindex othercognitive10 othercognitivespecify other_cognitive_code otherspeech10 otherspeechspecify otherpsychiatric10 otherpsychiatricspecify outcome excludereason diagnosisspreadsheet

//Rename & label variables

rename year year_volume

label variable year_volume "Year of the volume in which casenotes found"

label variable initials "Patient initials"

label variable rater "Person who did data extraction"

rename dateofentry extraction_date

rename diagnosispatientnotes diagnosis_notes

label variable diagnosis_notes "Diagnosis given on front sheet of Pt notes"

rename el diagnosis_EL

label variable diagnosis_EL "Diagnosis includes 'encephalitis lethargica', 'lethargic encephalitis' or 'epidemic encephalitis'"

rename parkinsonism diagnosis_parkinsonism

label variable diagnosis_parkinsonism "Diagnosis includes 'parkinsonism' or 'paralysis agitans'"

rename postencephalitis diagnosis_post_EL

label variable diagnosis_post_EL "Diagnosis mentions 'post-encephalitis' or suggests that these are post-acute sequelae of encephalitis"

rename acute diagnosis_acute

label variable diagnosis_acute "Diagnosis mentions encephalitis being acute"

rename query diagnosis_query

label variable diagnosis_query "Diagnosis is possible or probable"

rename comorbidity diagnosis_comorbid

label variable diagnosis_comorbid "Diagnosis mentions some comorbidity with encephalitis"

rename admissiondate admi_date

rename dischargedate disc_date

rename duration illness_duration

label variable illness_duration "Time since start of neuro(psychiatric) Sx of present illness in days"

rename maritalstatus marital_status

rename numberofepisodes episode_number

label variable episode_number "Number of distinct episodes"

rename subacuteonset subacute_onset

rename toneincreased tone_increased

rename timefromneuroonsettoparkins time_neuro_onset_park

label variable time_neuro_onset_park "Time (days) from neurological onset to parkinsonism"

rename febrileillnesstemporallyrelat febrile_illness

rename febrileillnessdate febrile_illness_date

rename neoplasiatype neoplasia_type

rename fhencephalitis fh_encephalitis

rename reducedconsciousness reduced_consciousness

label variable reduced_consciousness "Reduced consciousness"

label variable stupor "Stupor"

label variable agitation "Agitation"

label variable headache "Headache"

label variable nausea "Nausea"

label variable vomiting "Vomiting"

label variable hypersalivation "Hypersalivation"

rename sleepwakecyclereversal sleep_reversal

label variable sleep_reversal "Sleep-wake cycle reversal"

label variable insomnia "Insomnia"

label variable hypersomnia "Hypersomnia"

label variable tremor "Tremor"

label variable tone_increased "Increased tone"

label variable bradykinesia "Bradykinesia"

label variable tics "Tics"

label variable chorea "Chorea"

label variable myoclonus "Myoclonus"

label variable ataxia "Ataxia"

rename generalisedweakness weakness_general

label variable weakness_general "Generalised weakness"

rename focalweakness weakness_focal

label variable weakness_focal "Focal weakness"

rename maskedfacies masked_facies

label variable masked_facies "Masked facies"

rename reductioninspontaneousmovemen reduced_spont_mov

label variable reduced_spont_mov "Reduction in spontaneous movement"

rename akineticmutism akinetic_mutism

label variable akinetic_mutism "Akinetic mutism"

label variable catalepsy "Catalepsy"

rename waxyflexibility waxy_flex

label variable waxy_flex "Waxy flexibility"

label variable posturing "Posturing"

label variable mannerisms "Mannerisms"

label variable stereotypies "Stereotypies"

label variable grimacing "Grimacing"

label variable echopraxia "Echopraxia"

label variable dyskinesia "Dyskinesia"

label variable dystonia "Dystonia"

rename somatosensorysymptoms somatosens_sx

label variable somatosens_sx "Somatosensory symptoms"

label variable euphoria "Euphoria"

label variable depression "Depression"

rename emotionallability lability

label variable lability "Emotional lability"

label variable confusion "Confusion"

rename memoryimpairment memory_impairment

label variable memory_impairment "Memory impairment"

label variable bradyphrenia "Bradyphrenia"

label variable disorientation "Disorientation"

label variable dysphasia "Dysphasia"

label variable impairedattention "Impaired attention"

label variable impairednumericfunction "Impaired numeric function"

label variable nonspecificcognitiveimpairmen "Non-specific cognitive impairment"

label variable perseveration "Perseveration"

label variable rldisorientation "Right-left disorientation"

label variable subjectivecognitiveimpairment "Subjective cognitive impairment"

rename mutismorsignificantverbalre mute

label variable mute "Mutism"

label variable echolalia "Echolalia"

rename pressureofspeech pressure_of_speech

label variable pressure_of_speech "Pressure of speech"

label variable palilalia "Palilalia"

label variable verbigeration "Verbigeration"

label variable oligophasia "Oligophasia"

label variable bradyphasia "Bradyphasia"

label variable tachyphasia "Tachyphasia"

label variable quietspeech "Quiet speech"

rename monotonousspeech monotonous_speech

label variable monotonous_speech "Monotonous speech"

label variable dysarthricindistinctspeech "Dysarthria"

label variable anomia "Anomia"

label variable highpitchedspeech "High-pitched speech"

label variable lowpitched "Low-pitched speech"

label variable stammer "Stammer"

label variable verydeliberate "Very deliberate speech"

label variable speech_miscellaneous "Miscellaneous speech abnormality"

label variable negativism "Negativism"

label variable fatigue "Fatigue"

label variable impulsivity "Impulsivity"

rename sociallyunacceptablebehaviour unaccept_behav

label variable unaccept_behav "Socially unacceptable behaviour"

label variable hypersexuality "Hypersexuality"

rename obsessivecompulsivebehaviour obs_compuls

label variable obs_compuls "Obsessive-compulsive behaviour"

rename selfharming self_harm

label variable self_harm "Self-harm"

rename personalitychange personality_change

label variable personality_change "Personality change"

label variable hallucinations "Hallucination"

label variable delusions "Delusion"

label variable anxiety "Anxiety"

label variable apathy "Apathy"

label variable depersonalisationderealisation "Depersonalisation or derealisation"

rename affectiveblunting affect_blunt

label variable affect_blunt "Affective blunting"

label variable dissociation "Dissociation"

label variable parasomnia "Parasomnia"

rename reducedlibido reduced_libido

label variable reduced_libido "Reduced libido"

label variable psych_misc "Miscellaneous psychiatric symptoms"

label variable overweight "Overweight"

label variable underweight "Underweight"

rename increasedappetite appetite_increase

label variable appetite_increase "Increased appetite"

rename reducedoralintake intake_reduced

label variable intake_reduced "Reduced oral intake"

label variable polydipsia "Polydipsia"

rename iiiivvi iii_iv_vi

label variable i "I"

label variable ii "II"

label variable iii_iv_vi "III, IV, VI"

label variable v "V"

label variable vii "VII"

label variable viii "VIII"

label variable ix "IX"

label variable x "X"

label variable xi "XI"

label variable xii "XII"

rename oculogyriccrisis oculogyric_crisis

label variable oculogyric_crisis "Oculogyric crisis"

rename blurredvisionordoublevision vision_blurred

label variable vision_blurred "Blurred or double vision"

rename seizurenotexplainedbypreviou seizure_unexplained

rename hightemperature99fwithin5 temp_high

label variable temp_high "Hyperthermia"

rename lowtemperature97fwithin5 temp_low

label variable temp_low "Hypothermia"

rename fluctuatingtemperaturebothlo temp_fluct

label variable temp_fluct "Fluctuating temperature"

rename highhr100within5daysofa hr_high

label variable hr_high "Tachycardia"

rename lowhr60within5daysofadm hr_low

label variable hr_low "Bradycardia"

rename fluctuatinghrchangeof30bpm hr_fluct

label variable hr_fluct "Fluctuating heart rate"

rename centralhypoventilation central_hypovent

rename othercentralrespiratoryirregu central_resp_abn

rename csfresults10 csf

rename brainpathology brain_pathology

rename subjectivelyel subjective_EL

rename phototaken photo

rename exclreason excl_reason_code

rename oligophasianottoseverityof oligophasia

rename occupation occupation_raw

label variable occupation_raw "Raw occupation data"

rename notesifneededtoclarifyoccup occupation_notes

rename solventexposure1foryes0f exp_solvent

rename heavymetalexposure1foryes exp_heavy_metal

rename appearance csf_appearance

rename numberofcellspercubicmillim csf_cells

label variable csf_cells "Number of cells per cubic millimetre"

rename celltype csf_cell_type

rename albumingramsper100millilitr csf_albumin

label variable csf_albumin "Albumin in grams per 100 millilitres"

rename proteingramsper100millilitr csf_protein

label variable csf_protein "grams per 100 millilitres"

rename na1or0 csf_nonne

label variable csf_nonne "Nonne-Appelt test"

rename pandystest1or0 csf_pandy

rename langestest1or0 csf_lange

rename wassermannreactionwr1or0 csf_wasserman

rename sugardescription csf_sugar

rename bromides csf_bromide

rename chlorides csf_chloride

rename langeinterpretation csf_lange_interp

label variable csf_lange_interp "Interpretation of Lange's test"

rename causeofdeath death_cause

//Ensure correct variable types

describe

encode sex, gen (sex1)

drop sex

rename sex1 sex

tab marital_status, m

encode marital_status, gen(marital)

drop marital_status

tab handedness

encode handedness, gen (handed)

drop handedness

tab outcome_code

encode outcome_code, gen(outcome)

drop outcome_code

tab excl_reason_code

encode excl_reason_code, gen(excl_reason)

drop excl_reason_code

encode fh_encephalitis, gen(fh_encephalit)

drop fh_encephalitis

replace occupationofpatientpfath = "P" if occupationofpatientpfath=="p"

encode occupationofpatientpfath, gen(occupation_ref)

label define occupation_ref 1 "father", modify

label define occupation_ref 2 "husband", modify

label define occupation_ref 3 "mother", modify

label define occupation_ref 4 "patient", modify

label variable occupation_ref "Person to whom occupation refers"

drop occupationofpatientpfath

encode occupationalcategoryaccording, gen(occupation_cat)

drop occupationalcategoryaccording

encode sesseepage15forbreakdown, gen(seclass)

drop sesseepage15forbreakdown

replace addressurban1orrural0 = "" if addressurban1orrural0=="-"

destring addressurban1orrural0, gen(urban)

drop addressurban1orrural0

encode consultant, gen(consultant1)

drop consultant

rename consultant1 consultant

*The problem with year_volume is that it's a string, as it contains ranges of years

destring year_volume, gen(year_volume_median) force

replace year_volume_median = 1922 if year_volume=="1921-22"

replace year_volume_median = 1929 if year_volume=="1928-9"

//Drop all non-cases

*Remove all rows where no patients were found

tab excl_reason

drop if excl_reason==3

*Remove where there is no id

drop if id==""

//Replace missing data with zero, where appropriate

*This is legitimate, as we are attributing error to misclassification

recode diagnosis_EL diagnosis_parkinsonism diagnosis_post_EL diagnosis_acute diagnosis_query diagnosis_comorbid influenza febrile_illness neoplasia fh_encephalit subacute_onset reduced_consciousness stupor agitation headache nausea vomiting hypersalivation sleep_reversal insomnia hypersomnia tremor tone_increased bradykinesia tics chorea myoclonus ataxia weakness_general weakness_focal masked_facies reduced_spont_mov akinetic_mutism catalepsy waxy_flex posturing mannerisms stereotypies grimacing echopraxia dyskinesia dystonia somatosens_sx euphoria depression lability confusion memory_impairment bradyphrenia disorientation dysphasia impairedattention impairednumericfunction nonspecificcognitiveimpairmen perseveration rldisorientation subjectivecognitiveimpairment mute echolalia pressure_of_speech palilalia verbigeration oligophasia bradyphasia tachyphasia quietspeech monotonous_speech dysarthricindistinctspeech anomia highpitchedspeech lowpitched stammer verydeliberate speech_miscellaneous negativism fatigue impulsivity unaccept_behav hypersexuality obs_compuls self_harm personality_change hallucinations delusions anxiety apathy depersonalisationderealisation affect_blunt dissociation parasomnia reduced_libido psych_misc overweight underweight appetite_increase intake_reduced polydipsia i ii iii_iv_vi ophthalmoplegia v vii viii ix x xi xii oculogyric_crisis vision_blurred staring seizure seizure_unexplained temp_high temp_low temp_fluct hr_high hr_low hr_fluct central_hypovent central_resp_abn csf brain_pathology subjective_EL photo (. = 0)

//Label values

label define EL_not_EL 0 "Related diagnoses" 1 "Encephalitis lethargica"

label values diagnosis_EL EL_not_EL

//Create case-control variable

gen case = 1

replace case = 0 if strpos(id, "C")==1

label define case_control 0 "control" 1 "case"

label values case case_control

//Check for duplicate IDs

codebook id

duplicates list id

save "${ROOT}002 DTA files\002 Cleaned data.dta", replace

log close

## 002 Create secondary variables.do

log using "${ROOT}004 Log files\002 Create secondary variables.log", replace

use "${ROOT}002 DTA files\002 Cleaned data.dta", clear

//Sort date variables

format %td extraction_date admi_date disc_date

gen admi_year = year(admi_date)

gen admi_duration = disc_date - admi_date

sum admi_duration

gen illness_duration_year = illness_duration/365.25

gen illness_onset = admi_date - illness_duration

format %td illness_onset

gen illness_onset_year = year(illness_onset)

gen time_neuro_onset_park_yr = time_neuro_onset_park/365.25

//Create influenza & febrile illness within time window variables

gen influenza_febrile = 0

replace influenza_febrile = 1 if influenza==1 | febrile_illness==1

gen influenza_1yr = 0

foreach v of varlist influenzadate1 - influenzadate8 {

replace influenza_1yr = 1 if `v'==illness_onset_year

replace influenza_1yr = 1 if `v'==illness_onset_year - 1

}

label variable influenza_1yr "Had influenza in the calendar year of or the year before illness onset"

gen febrile_illness_1yr = 0

replace febrile_illness_1yr = 1 if febrile_illness_date==illness_onset_year | febrile_illness_date==illness_onset_year - 1

label variable febrile_illness_1yr "Had febrile illness in calendar year of or year before illness onset"

gen influenza_febrile_1yr = 0

replace influenza_febrile_1yr = 1 if influenza_1yr==1 | febrile_illness_1yr==1

label variable influenza_febrile_1yr "Had influenza or febrile illness in calendar year of or year before illness onset"

//Create diagnostic variable

*diagnosis_EL has 0 and 1 the wrong way round for a graph - create a variable that flips it

gen diagnosis_not_EL = 1 - diagnosis_EL

label define not_EL_EL 1 "Implicit diagnosis of EL" 0 "Explicit diagnosis of EL"

label values diagnosis_not_EL not_EL_EL

**# Create variable list macros

global loc_agitation "reduced_consciousness stupor agitation"

global gastro_nutri "nausea vomiting hypersalivation overweight underweight appetite_increase intake_reduced polydipsia"

global sleep "sleep_reversal insomnia hypersomnia parasomnia"

global motor "tremor tone_increased bradykinesia tics chorea myoclonus ataxia weakness_general weakness_focal masked_facies reduced_spont_mov akinetic_mutism catalepsy waxy_flex posturing mannerisms stereotypies grimacing echopraxia dyskinesia dystonia"

global cognition "confusion memory_impairment bradyphrenia disorientation dysphasia impairedattention impairednumericfunction nonspecificcognitiveimpairmen perseveration rldisorientation subjectivecognitiveimpairment anomia"

global speech "mute echolalia pressure_of_speech palilalia verbigeration oligophasia bradyphasia tachyphasia quietspeech monotonous_speech dysarthricindistinctspeech highpitchedspeech lowpitched stammer verydeliberate speech_miscellaneous"

global psychiatric "euphoria depression lability negativism fatigue impulsivity unaccept_behav hypersexuality obs_compuls self_harm personality_change hallucinations delusions anxiety apathy depersonalisationderealisation affect_blunt dissociation reduced_libido psych_misc"

global cranial_nerves "i ii iii_iv_vi v vii viii ix x xi xii"

global ophth "ophthalmoplegia oculogyric_crisis vision_blurred staring"

global vital_signs "temp_high temp_low temp_fluct hr_high hr_low hr_fluct central_hypovent central_resp_abn"

global neuro_other "headache somatosens_sx seizure seizure_unexplained"

**# Create higher level clinical categories

gen loc_agitation = 0

label variable loc_agitation "Level of arousal abnormalities"

foreach v in $loc_agitation {

replace loc_agitation = 1 if `v'==1

}

gen gastro_nutri = 0

label variable gastro_nutri "GI and nutritional features"

foreach v in $gastro_nutri {

replace gastro_nutri = 1 if `v'==1

}

gen sleep = 0

label variable sleep "Sleep disorders"

foreach v in $sleep {

replace sleep = 1 if `v'==1

}

gen motor = 0

label variable motor "Motor features"

foreach v in $motor {

replace motor = 1 if `v'==1

}

gen cognition = 0

label variable cognition "Cognitive features"

foreach v in $cognition {

replace cognition = 1 if `v'==1

}

gen speech = 0

label variable speech "Speech disorders"

foreach v in $speech {

replace speech = 1 if `v'==1

}

gen psychiatric = 0

label variable psychiatric "Psychiatric features"

foreach v in $psychiatric {

replace psychiatric = 1 if `v'==1

}

gen cranial_nerves = 0

label variable cranial_nerves "Cranial nerve findings"

foreach v in $cranial_nerves {

replace cranial_nerves = 1 if `v'==1

}

gen ophth = 0

label variable ophth "Ophthalmological features"

foreach v in $ophth {

replace ophth = 1 if `v'==1

}

gen vital_signs = 0

label variable vital_signs "Abnormalities of vital signs"

foreach v in $vital_signs {

replace vital_signs = 1 if `v'==1

}

gen neuro_other = 0

label variable neuro_other "Other neurological abnormalities"

foreach v in $neuro_other {

replace neuro_other = 1 if `v'==1

}

global category_list "loc_agitation gastro_nutri sleep motor cognition speech psychiatric cranial_nerves ophth vital_signs neuro_other"

**# Create custom groups of clinical features

gen parkinsonism = 0

replace parkinsonism=1 if tremor==1 | tone_increased==1 | bradykinesia==1

**# Age variables

gen child = .

replace child = 1 if age<18

replace child = 0 if age>=18

label variable child "Age<18 on admission"

label define child 0 "adult" 1 "child"

label values child child

**# CSF variables

table csf_appearance

gen csf_appear = ""

replace csf_appear = "blood" if csf_appearance=="blood contaminated" | csf_appearance=="bloodstained" | csf_appearance=="clear colourless with some blood" | csf_appearance=="red fluid" | csf_appearance=="slightly bloodstained" | csf_appearance=="with blood" | csf_appearance=="yellowish, turbid due to blood cells"

replace csf_appear = "clear" if csf_appearance=="clear" | csf_appearance=="clear colourless"

replace csf_appear = "yellow/turbid" if csf_appearance=="dark yellow" | csf_appearance=="slightly turbid, yellowish" | csf_appearance=="slightly yellow" | csf_appearance=="turbid colourless" | csf_appearance=="yellowish, no coagulum"

table csf_appearance csf_appear

table csf_cells

*Where a range is given (e.g. 5 - 10) replace this with the half-way point

replace csf_cells = "7.5" if csf_cells==" 5-Oct"

replace csf_cells = "12.5" if csf_cells=="Oct-15"

replace csf_cells = "0" if csf_cells=="< 1"

replace csf_cells = "0" if csf_cells=="<1"

replace csf_cells = "2.5" if csf_cells=="< 5"

*Other obvious changes

replace csf_cells = "138" if csf_cells=="138 RBC / 1"

replace csf_cells = "0" if csf_cells=="only those due to blood admixture"

replace csf_cells = "0" if csf_cells=="<1"

destring csf_cells, replace

gen csf_cells_high = .

replace csf_cells_high = 0 if csf_cells<.

replace csf_cells_high=1 if csf_cells>5 & csf_cells<.

*Cell types

*Just coding whether cells are predominantly RBCs or WBCs

tab csf_cell_type

gen csf_cell_type_white = .

replace csf_cell_type_white = 1 if csf_cell_type=="10% large mono" | csf_cell_type=="15% large mono" | csf_cell_type=="2% large mono" | csf_cell_type=="20% large mono" | csf_cell_type=="8% poly, 8% large mono" | csf_cell_type=="an occasional lymphocyte" | csf_cell_type=="large mono" | csf_cell_type=="large monos (5%)" | csf_cell_type=="lymphocytes" | csf_cell_type=="medium mono" | csf_cell_type=="mono" | csf_cell_type=="mononuclear" | csf_cell_type=="mononuclear, phagocytes, indefinite polymorphs with lobed nucleus" | csf_cell_type=="polymoprh due to blood" | csf_cell_type=="small / large mono" | csf_cell_type=="small lympho" | csf_cell_type=="small mono" | csf_cell_type=="small mononuclears" | csf_cell_type=="small monos" | csf_cell_type=="small to large mono" | csf_cell_type=="small to large mononuclears" | csf_cell_type=="small to large monos" | csf_cell_type=="small to large monos and polymorph" | csf_cell_type=="small to medium mononuclears"

replace csf_cell_type_white = 0 if csf_cell_type=="50 red cells, 1 lymphocyte" | csf_cell_type=="RBC" | csf_cell_type=="due to blood"

*Albumin

tab csf_albumin

replace csf_albumin = "0.0125" if csf_albumin=="< 0.025"

destring csf_albumin, replace

*Protein

tab csf_protein

replace csf_protein = "" if csf_protein=="not increased"

destring csf_protein, replace

*Nonne

tab csf_nonne

*Pandy

tab csf_pandy

*Lange

tab csf_lange_interp

*Sugar - too few to be interesting

tab csf_sugar

*Bromide - too few to be interesting

tab csf_bromide

*Chloride - too few to be interesting

tab csf_chloride

*Pleocytosis

*Graus et al, 2016 define this as WCC > 5 cells per cubic millimetre

*We therefore want to exclude samples where cell type was noted to be RBC

*To be conservative, we can also exclude any samples that appear to be blood-stained

gen csf_pleocytosis = .

replace csf_pleocytosis = 0 if csf==1

replace csf_pleocytosis = 1 if csf_cells_high==1

replace csf_pleocytosis = 0 if csf_cell_type_white==0 | csf_appear=="blood"

//Stratum variable for matching

*year_volume, sex, consultant

egen volume = concat(year_volume sex consultant), decode punct(_)

encode volume, gen(volume_num)

save "${ROOT}002 DTA files\003 Secondary variables added.dta", replace

log close

## 003 Eligibility.do

log using "${ROOT}004 Log files\003 Eligibility.log", replace

use "${ROOT}002 DTA files\003 Secondary variables added.dta", clear

//Establish current inclusions/exclusions

count

tab case

tab include case

tab excl_reason case

//Create -v_basic- variable

*v_basic = 1 if diagnosis is EL/similar (cases only), there are adequate notes and there is no prior admission

gen v_basic = 0

replace v_basic = 1 if include=="1"

label variable v_basic "1 if EL diag (cases only), adequate notes and no prior admission"

tab v_basic case

*v_def = 1 if v_basic and diagnosis is definite (i.e. not with some comorbidity and not possible/probable)

*For controls, v_def is the same as v_basic

tab diagnosis_query case if v_basic==1

tab diagnosis_comorbid case if v_basic==1 & diagnosis_query==0

gen v_def = 0

replace v_def = 1 if v_basic==1 & diagnosis_query==0 & diagnosis_comorbid==0

label variable v_def "v_basic and diagnosis definite without diagnostic comorbidity. For controls, same as v_basic."

tab v_def case if v_basic==1

save "${ROOT}002 DTA files\004 Eligibility defined.dta", replace

log close

## 004 Basic descriptive stats.do

log using "${ROOT}004 Log files\004 Basic descriptives.log", replace

use "${ROOT}002 DTA files\XXX Ready for analysis.dta", clear

**# Demographics

*Age at hospital admission

sort case

tab admi_year if v_def==1 & case==1

histogram age if v_def==1 & case==1

by case: sum age if v_def==1, detail

*One age missing - not in original notes

vioplot age if v_def==1, over(case) graphregion(fcolor(white)) ytitle ("Age at admission / years")

graph save "Graph" "${ROOT}005 Graphs\008.1 - Violin plot of age.gph", replace

graph export "${ROOT}005 Graphs\008.1 - Violin plot of age.png", as(png) name("Graph") replace

*Age in 2023 (for GDPR purposes)

gen age_2023 = 2023 - (admi_year - age)

sum age_2023

sort age_2023

br id admi_year age age_2023

*Sex

tab case sex if v_def==1, row mi

*Marital status

tab case marital if v_def==1, row mi

*Handedness

tab case handed if v_def==1, row mi

**# Urbanicity

tab case urban if v_def==1, row mi

melogit case urban year_volume_median sex if v_def==1 & occupation_ref==4 || consultant: , or

melogit case urban year_volume_median sex age if v_def==1 & occupation_ref==4 || consultant: , or

**# Occupation & exposures

tab case occupation_ref if v_def==1, row mi

tab occupation_cat case if v_def==1, col mi

tab case exp_solvent if v_def==1, row mi

tab case exp_heavy_metal if v_def==1, row mi

*Limit to individuals who have had exposure themselves

tab occupation_cat case if v_def==1 & occupation_ref==4, col mi

*Multilevel model using consultant as higher level

tab case exp_solvent if v_def==1 & occupation_ref==4, row mi

melogit case exp_solvent year_volume_median sex if v_def==1 & occupation_ref==4 || consultant: , or

melogit case exp_solvent year_volume_median sex age if v_def==1 & occupation_ref==4 || consultant: , or

tab case exp_heavy_metal if v_def==1 & occupation_ref==4, row mi

melogit case exp_heavy_metal year_volume_median sex if v_def==1 & occupation_ref==4 || consultant: , or

melogit case exp_heavy_metal year_volume_median sex age if v_def==1 & occupation_ref==4 || consultant: , or

**# Conditional logistic regression for environmental exposures

clogit case exp_solvent if v_def==1 & occupation_ref==4, strata(volume_num) or

clogit case exp_solvent age if v_def==1 & occupation_ref==4, strata(volume_num) or

clogit case exp_heavy_metal if v_def==1 & occupation_ref==4, strata(volume_num) or

clogit case exp_heavy_metal age if v_def==1 & occupation_ref==4, strata(volume_num) or

*Conditional logistic regression models don't work well because all the case-only strata are omitted from the analysis

**# Socioeconomic status

tab seclass case if v_def==1, col mi

**# Hospitalisation details

sort case

by case: sum admi_year if v_def==1, detail

graph hbar (count) if v_def==1 & case==1, over(admi_year)

twoway histogram admi_year if v_def==1 & case==1, discrete frequency width(1)

graph save "Graph" "${ROOT}005 Graphs\001.1 - Bar chart of years of admission (cases).gph", replace

graph export "${ROOT}005 Graphs\001.1 - Bar chart of years of admission (cases).png", as(png) name("Graph") replace

*Consultant

tab consultant case if v_def==1, col mi

sort case

by case: tab1 consultant if v_def==1, mi sort

*Admission duration

histogram admi_duration if v_def==1 & case==1

by case: sum admi_duration if v_def==1, detail

vioplot admi_duration if v_def==1, over(case) graphregion(fcolor(white)) ytitle ("Admission duration / days")

graph save "Graph" "${ROOT}005 Graphs\008.2 - Violin plot of admission duration.gph", replace

graph export "${ROOT}005 Graphs\008.2 - Violin plot of admission duration.png", as(png) name("Graph") replace

*Outcome

tab outcome case if v_def==1, col mi

**# Diagnosis

tab diagnosis_EL if case==1 & v_def==1

tab diagnosis_parkinsonism if case==1 & v_def==1

tab diagnosis_post_EL if case==1 & v_def==1

tab diagnosis_acute if case==1 & v_def==1

**# Disease timing

*Duration at admission

by case: sum illness_duration if v_def==1, detail

by case: sum illness_duration_year if v_def==1, detail

*Year of first illness

sum illness_onset_year if v_def==1 & case==1, detail

graph hbar (count) if v_def==1 & case==1, over(illness_onset_year)

twoway histogram illness_onset_year if v_def==1 & case==1, discrete frequency width(1)

graph save "Graph" "${ROOT}005 Graphs\001.2 - Bar chart of year of illness onset (cases).gph", replace

graph export "${ROOT}005 Graphs\001.2 - Bar chart of year of illness onset (cases).png", as(png) name("Graph") replace

*Combine year of first illness with year of admission

twoway (histogram illness_onset_year if v_def==1 & case==1, discrete frequency width(1) color(red%30)) (histogram admi_year if v_def==1 & case==1, discrete frequency width(1) color(green%30)) , legend(order (1 "Illness onset" 2 "Hospital admission")) graphregion(color(white)) ytitle("Cases") xtitle("Year") xtic(1900(5)1950)

graph save "Graph" "${ROOT}005 Graphs\001.3 - Bar chart of years of admission and illness onset (cases).gph", replace

graph export "${ROOT}005 Graphs\001.3 - Bar chart of years of admission and illness onset (cases).png", as(png) name("Graph") replace

*Number of episodes

tab episode_number if case==1 & v_def==1, mi

*Time from neurological onset to parkinsonism

**Days

sum time_neuro_onset_park if case==1 & v_def==1, detail

vioplot time_neuro_onset_park if v_def==1, graphregion(fcolor(white)) ytitle ("Time / days") xlabel("")

**Months

gen time_neuro_onset_park_m = time_neuro_onset_park/30.4375

sum time_neuro_onset_park_m if case==1 & v_def==1, detail

vioplot time_neuro_onset_park_m if v_def == 1, graphregion(fcolor(white)) ytitle("Time / months") xlabel("") ymtick(0(12)300) ytick(0(60)300) ylabel(0(60)300)

graph export "${ROOT}005 Graphs\008.3 - Violin plot of time to parkinsonism months.png", as(png) name("Graph") replace

**Years

sum time_neuro_onset_park_yr if case==1 & v_def==1, detail

count if time_neuro_onset_park==0 & case==1 & v_def==1

count if time_neuro_onset_park<=7 & case==1 & v_def==1

vioplot time_neuro_onset_park_yr if v_def==1, graphregion(fcolor(white)) ytitle ("Time / years") xlabel("")

graph save "Graph" "${ROOT}005 Graphs\008.3 - Violin plot of time to parkinsonism.gph", replace

graph export "${ROOT}005 Graphs\008.3 - Violin plot of time to parkinsonism.png", as(png) name("Graph") replace

*(Sub)acute onset

tab subacute_onset if case==1 & v_def==1

*Number presenting to the hospital in their acute illness (within 30 days)

gen presentation_acute = 0

replace presentation_acute =1 if illness_duration <=30

tab presentation_acute if case==1 & v_def==1

**# Circumstances of disease onset

*Influenza

tab influenza if case==1 & v_def==1

*Febrile illness

tab febrile_illness if case==1 & v_def==1

*Neoplasia

tab neoplasia if case==1 & v_def==1

tab neoplasia_type if case==1 & v_def==1

*FH encephalitis

tab fh_encephalit if case==1 & v_def==1

**# Control diagnoses

tab diagnosis_cat_control if v_def==1 & case==0, sort

log close

## 006 Correspondence to diagnostic criteria.do

log using "${ROOT}004 Log files\006 Correspondence to diagnostic criteria.log", replace

use "${ROOT}002 DTA files\XXX Ready for analysis.dta", clear

**# Catatonia

*DSM-5 criteria require 3 of the following: catalepsy, waxy flexibility, stupor, agitation, mutism, negativism, posturing, mannerisms, stereotypies, grimacing, echolalia, echopraxia

gen catatonia_score = catalepsy + waxy_flex + stupor + agitation + mute + negativism + posturing + mannerisms + stereotypies + grimacing + echolalia + echopraxia

label variable catatonia_score "Number of DSM-5 catatonic features"

gen catatonia_3 = 0

replace catatonia_3 = 1 if catatonia_score >=3

label variable catatonia_3 "1 if have 3+ DSM-5 catatonic features"

tab case catatonia_3 if v_def==1

*Of course, a patient may have exhibited these features many years apart. We should verify this diagnosis by checking whether features were within, say, a week

*Check back with original data

browse id catatonia_score catatonia_3 catalepsy waxy_flex stupor agitation mute negativism posturing mannerisms stereotypies grimacing echolalia echopraxia if catatonia_3 ==1 & case==1 & v_def==1

gen catatonia_dsm = .

replace catatonia_dsm = 1 if id == "25" | id == "132" | id == "194" | id == "207" | id == "211" | id == "212" | id == "218" | id == "493" | id == "587" | id == "851" | id == "860"

replace catatonia_dsm = 0 if id == "276" | id == "531" | id == "606"

replace catatonia_dsm = 0 if catatonia_dsm==.

tab catatonia_dsm if case==1 & v_def==1, mi

**# Graus criteria: possible autoimmune encephalitis

*Criteria: All 3 of

*• 1 Subacute onset (rapid progression of less than 3 months) of working memory deficits (short-term memory loss), altered mental status (altered level of consciousness, lethargy or personality change), or psychiatric symptoms

*• 2 At least one of the following: • New focal CNS findings • Seizures not explained by a previously known seizure disorder • CSF pleocytosis (white blood cell count of more than five cells per mm³) • MRI features suggestive of encephalitis†

*• 3 Reasonable exclusion of alternative causes (appendix)

*Criterion 1

gen alt_ment_stat = 0

replace alt_ment_stat = 1 if reduced_consciousness==1 | confusion==1 | disorientation==1| fatigue==1 | apathy==1 | personality_change==1 | impulsivity==1 | unaccept_behav==1

gen psych_sx = 0

replace psych_sx = 1 if tics==1 | euphoria==1 | depression==1 | lability==1 | hypersexuality==1 | obs_compuls==1 | self_harm==1 | hallucinations==1 | delusions==1 | anxiety==1 | depersonalisationderealisation==1 | affect_blunt==1 | dissociation==1 | parasomnia==1 | reduced_libido==1 | psych_misc==1

gen graus_pos1 = 0

replace graus_pos1 = 1 if subacute_onset==1 & (memory_impairment==1 | alt_ment_stat==1 | psych_sx==1)

tab graus_pos1 if case==1 & v_def==1, mi

*Criterion 2

gen focal_cns = 0

replace focal_cns = 1 if tremor==1 | tone_increased==1 | bradykinesia==1 | chorea==1 | myoclonus==1 | ataxia==1 | weakness_focal==1 | masked_facies==1 | akinetic_mutism==1 | dyskinesia==1 | dystonia==1 | somatosens_sx==1 | dysphasia==1 | anomia ==1 |i==1 | ii==1 | iii_iv_vi==1 | ophthalmoplegia==1 | v==1 | vii==1 | viii==1 | ix==1 | x==1 | xi==1 | xii==1 | oculogyric_crisis==1 | vision_blurred==1

gen graus_pos2 = 0

replace graus_pos2 = 1 if focal_cns==1 | seizure_unexplained==1 | csf_pleocytosis==1

tab graus_pos2 if case==1 & v_def==1, mi

*Criteria 1 & 2

gen graus_pos = 0

replace graus_pos = 1 if graus_pos1==1 & graus_pos2==1

tab graus_pos if case==1 & v_def==1, mi

*Limitations:

*Subacute onset might not necessarily refer to the particular clinical features specified in Criterion 1

*We have not specifically assessed whether all focal CNS findings are new (though they were overwhelmingly)

*Not all Pts had CSF results and we don't have MRI results, so Criterion 2 may be an underestimate

*We haven't assessed Criterion 3, though given the lack of availability of neuroimaging, we might perhaps assume that alternative causes haven't been ruled out

**# Graus criteria: probable NMDARE

*Criteria: All 3 of

*• 1 Rapid onset (less than 3 months) of at least four of the six following major groups of symptoms: • Abnormal (psychiatric) behaviour or cognitive dysfunction • Speech dysfunction (pressured speech, verbal reduction, mutism) • Seizures • Movement disorder, dyskinesias, or rigidity/abnormal postures • Decreased level of consciousness • Autonomic dysfunction or central hypoventilation

*• 2 At least one of the following laboratory study results: • Abnormal EEG (focal or diff use slow or disorganised activity, epileptic activity, or extreme delta brush) • CSF with pleocytosis or oligoclonal bands

*• 3 Reasonable exclusion of other disorders (appendix)

*Criterion 1

gen abn_behav_cog = 0

replace abn_behav_cog = 1 if stupor==1 | agitation ==1 | tics ==1 | euphoria ==1 | depression ==1 | lability ==1 | confusion ==1 | memory_impairment ==1 | bradyphrenia ==1 | disorientation ==1 | dysphasia ==1 | impairedattention ==1 | impairednumericfunction ==1 | nonspecificcognitiveimpairmen ==1 | perseveration ==1 | rldisorientation ==1 | anomia ==1 | impulsivity ==1 | unaccept_behav ==1 | hypersexuality ==1 | obs_compuls ==1 | self_harm ==1 | personality_change ==1 | hallucinations ==1 | delusions ==1 | anxiety ==1 | apathy ==1 | depersonalisationderealisation ==1 | affect_blunt ==1 | dissociation ==1 | parasomnia ==1 | reduced_libido ==1 | psych_misc==1

gen speech_dys = 0

replace speech_dys = 1 if pressure_of_speech ==1 | mute==1 | oligophasia==1

gen mov_dis =0

replace mov_dis = 1 if tremor==1 | tone_increased ==1 | bradykinesia ==1 | tics ==1 | chorea ==1 | myoclonus ==1 | ataxia ==1 | masked_facies ==1 | reduced_spont_mov ==1 | akinetic_mutism ==1 | catalepsy ==1 | waxy_flex ==1 | posturing ==1 | mannerisms ==1 | stereotypies ==1 | grimacing ==1 | echopraxia ==1 | dyskinesia ==1 | dystonia ==1 | oculogyric_crisis ==1

gen autonomic_dys = 0

replace autonomic_dys = 1 if temp_high==1 | temp_low ==1 | temp_fluct ==1 | hr_high ==1 | hr_low ==1 | hr_fluct ==1 | central_hypovent==1

gen graus_nmda1 = 0

replace graus_nmda1 = 1 if subacute_onset==1 & (abn_behav_cog + speech_dys + seizure + mov_dis + reduced_consciousness + autonomic_dys >=4)

tab graus_nmda1 if case==1 & v_def==1, mi

*Criterion 2

table csf_pleocytosis if case==1 & v_def==1, mi

gen graus_nmda2 = csf_pleocytosis

*Criteria 1 & 2

gen graus_nmda_prob = 0

replace graus_nmda_prob = 1 if graus_nmda1==1 & graus_nmda2==1

tab graus_nmda_prob if case==1 & v_def==1, mi

tab graus_nmda_prob if case==1 & v_def==1 & csf_pleocytosis<., mi

*Limitations:

*Criterion 2 is only evaluated in a minority of patients, so we don't know about most of them

*We haven't assessed Criterion 3, though given the lack of availability of neuroimaging, we might perhaps assume that alternative causes haven't been ruled out

*Number meeting Criterion 1 gives an upper bound for number who may have NMDARE though, i.e. very small

log close

## 007 Influenza and febrile illnesses.do

log using "${ROOT}004 Log files\007 Influenza and febrile illness.log", replace

use "${ROOT}002 DTA files\XXX Ready for analysis.dta", clear

**# Any episode of influenza or febrile illness

tab influenza if case==1 & v_def==1, mi

tab febrile_illness if case==1 & v_def==1, mi

tab influenza_febrile if case==1 & v_def==1, mi

**# An episode of influenza or febrile illness in calendar year of illness onset or previous calendar year

tab influenza_1yr if case==1 & v_def==1, mi

tab febrile_illness_1yr if case==1 & v_def==1, mi

tab influenza_febrile_1yr if case==1 & v_def==1, mi

ci proportions influenza_febrile_1yr case if case==1 & v_def==1

**# Comparison to control group

tab influenza_febrile_1yr if case==0 & v_def==1, mi

ci proportions influenza_febrile_1yr case if case==0 & v_def==1

*Case-control study, with exposure as flu/febrile illness and outcome as development of EL

*Primary analysis: relationship between influenza in previous 1yr and subsequent EL

tab case influenza_febrile_1yr if v_def==1, row

melogit case influenza_febrile_1yr year_volume_median sex if v_def==1 & occupation_ref==4 || consultant: , or

melogit case influenza_febrile_1yr year_volume_median sex age if v_def==1 & occupation_ref==4 || consultant: , or

**# FH encephalitis

tab fh_encephalit if case==1 & v_def==1

log close

## 008 Geographical distribution.do

log using "${ROOT}004 Log files\008 Geographical distribution.log", replace

use "${ROOT}002 DTA files\XXX Ready for analysis.dta", clear

**# Basic descriptives

tab county case if v_def==1, col mi

tab urban case if v_def==1, col mi chi2

**# Collapse original data to counties

keep if case==1 & v_def==1

tab county

encode county, gen(county_code)

collapse (count) checked, by(county_code)

rename checked num_cases

label variable num_cases "Number of cases"

decode county_code, gen(county_name)

save "${ROOT}002 DTA files\B001 Cases per county.dta", replace

**# Import map

*County data from https://www.ukpostcode.net/shapefile-of-uk-administrative-counties-wiki-16.html

*shp2dta using "${ROOT}001 Raw data\Geo data\Map_UK.dbf", database("${ROOT}002 DTA files\A001 uk_counties") coordinates("${ROOT}002 DTA files\A002 uk_coord") genid(county_id) replace

clear

cd "${ROOT}002 DTA files"

shp2dta using "${ROOT}001 Raw data\Geo data\Map_UK.shp", database(A001 uk_counties) coordinates(uk_coord) genid(id) replace

*spshape2dta "${ROOT}001 Raw data\Geo data\Map_UK.shp", replace saving(A001 uk_counties)

*Barnet seems to be missing

use "A001 uk_counties", clear

describe

list ID_2 NAME_2

rename NAME_2 county_name

save "${ROOT}002 DTA files\A001 uk_counties.dta", replace

**# Merge in data on cases

use "A001 uk_counties", clear

merge 1:1 county_name using "${ROOT}002 DTA files\B001 Cases per county.dta"

*RoI, Barnet and missing data didn't merge

replace num_cases=0 if _merge==1

drop if _merge==2

*Make group categories for cases

gen cat_cases = "0"

replace cat_cases = "1 - 5" if num_cases >0

replace cat_cases = "6 - 10" if num_cases >5

replace cat_cases = "11 - 15" if num_cases >10

replace cat_cases = "16 - 20" if num_cases >15

replace cat_cases = "21 - 25" if num_cases >20

replace cat_cases = "26 - 30" if num_cases >25

replace cat_cases = "31 - 35" if num_cases >30

replace cat_cases = "36 - 40" if num_cases >35

replace cat_cases = "41 - 45" if num_cases >40

encode cat_cases, gen(cases_category)

save "${ROOT}002 DTA files\B002 Cases per county merged with map.dta", replace

**# Draw map

use "${ROOT}002 DTA files\B002 Cases per county merged with map.dta", replace

spmap num_cases using uk_coord, id(id) cln(10) fcolor(Blues2) osize(vvthin ..) clmethod(custom) clbreaks(0 0.5 5.5 10.5 15.5 20.5 25.5 30.5 35.5 40.5 45.5) legstyle(2) legend(label (2 "0") label(3 "1 - 5") label(4 "6 - 10") label(5 "11 - 15") label(6 "16 - 20") label(7 "21 - 25") label(8 "26 - 30") label(9 "31 - 35") label(10 "36 - 40") label(11 "40 - 45") ) legtitle("Case numbers")

graph save "Graph" "${ROOT}005 Graphs\003.1 - Intensity map for case numbers.gph", replace

graph export "${ROOT}005 Graphs\003.1 - Intensity map for case numbers.png", as(png) name("Graph") replace width(5000)

log close

## 011 Validation of Lees criteria.do

log using "${ROOT}004 Log files\011 Validation of Lees criteria.log", replace

use "${ROOT}002 DTA files\XXX Ready for analysis.dta", clear

**# Make programs for subsequent analysis

program drop report_ci

/* Program to report CIs for sensitivity and specificity */

program report_ci

args clinical_criterion

count if case==1 & `clinical_criterion'==1 & v_def==1

scalar tp_total = r(N)

count if case==0 & `clinical_criterion'==1 & v_def==1

scalar fp_total = r(N)

count if case==0 & `clinical_criterion'==0 & v_def==1

scalar tn_total= r(N)

count if case==1 & `clinical_criterion'==0 & v_def==1

scalar fn_total= r(N)

scalar case_total = tp_total + fn_total

scalar control_total = tn_total + fp_total

di "Sensitivity"

cii proportions case_total tp_total

di "Specificity"

cii proportions control_total tn_total

end

*Howard & Lees criteria:

*Acute of subacute encephalitic illness with at least 3 of the following:

*• Signs of basal ganglia involvement

*• Oculogyric crises

*• Ophthalmoplegia

*• Obsessive-compulsive behaviour

*• Akinetic mutism

*• Central respiratory irregularities

*• Somnolence and/or sleep inversion

**# Create appropriate variables

gen basal_gang = 0

label variable basal_gang "Signs of basal ganglia involvement"

*Howard/Lees describe this as 'cogwheel rigidity, expressionless mask-like facies and slowness and paucity of movement'

replace basal_gang = 1 if tremor==1 | tone_increased==1 | masked_facies==1 | bradykinesia==1 | reduced_spont_mov==1

gen resp_abn = 0

label variable central_resp_abn "Central respiratory irregularities"

replace resp_abn = 1 if central_hypovent==1 | central_resp_abn==1

gen sleep_lees = 0

label variable sleep_lees "Somnolence and/or sleep inversion"

replace sleep_lees = 1 if sleep_reversal==1 | hypersomnia==1

gen howard_lees=0

label variable howard_lees "Meets Howard & Lees criteria for EL"

replace howard_lees=1 if (subacute_onset==1) & (basal_gang + oculogyric_crisis + ophthalmoplegia + obs_compuls + akinetic_mutism + resp_abn + sleep_lees)>=3

**# Overall sensitivity & specificity

roctab case howard_lees if v_def==1, binomial detail summary table graph

graph save "Graph" "${ROOT}005 Graphs\005.1 - Howard-Lees criteria ROC.gph", replace

graph export "${ROOT}005 Graphs\005.1 - Howard-Lees criteria ROC.png", as(png) name("Graph") replace

report_ci howard_lees

**# Sensitivity & specificity of individual clinical features

*(Sub)acute onset

roctab case subacute if v_def==1, binomial detail summary table graph

graph save "Graph" "${ROOT}005 Graphs\005.2 - Subacute onset ROC.gph", replace

graph export "${ROOT}005 Graphs\005.2 - Subacute onset ROC.png", as(png) name("Graph") replace

report_ci subacute

*Signs of basal ganglia involvement

roctab case basal_gang if v_def==1, binomial detail summary table graph

graph save "Graph" "${ROOT}005 Graphs\005.3 Basal ganglia involvement ROC.gph", replace

graph export "${ROOT}005 Graphs\005.3 Basal ganglia involvement ROC.png", as(png) name("Graph") replace

report_ci howard_lees

*Oculogyric crisis

roctab case oculogyric_crisis if v_def==1, binomial detail summary table graph

graph save "Graph" "${ROOT}005 Graphs\005.4 - Oculogyric crisis ROC.gph", replace

graph export "${ROOT}005 Graphs\005.4 - Oculogyric crisis ROC.png", as(png) name("Graph") replace

report_ci howard_lees

*Ophthalmoplegia

roctab case ophthalmoplegia if v_def==1, binomial detail summary table graph

graph save "Graph" "${ROOT}005 Graphs\005.5 - Ophthalmoplegia ROC.gph", replace

graph export "${ROOT}005 Graphs\005.5 - Ophthalmoplegia ROC.png", as(png) name("Graph") replace

report_ci howard_lees

*Obsessive-compulsive features

roctab case obs_compuls if v_def==1, binomial detail summary table graph

graph save "Graph" "${ROOT}005 Graphs\005.6 - Obsessive-compulsive features ROC.gph", replace

graph export "${ROOT}005 Graphs\005.6 - Obsessive-compulsive features ROC.png", as(png) name("Graph") replace

report_ci howard_lees

*Akinetic mutism

roctab case akinetic_mutism if v_def==1, binomial detail summary table graph

graph save "Graph" "${ROOT}005 Graphs\005.7 - Akinetic mutism ROC.gph", replace

graph export "${ROOT}005 Graphs\005.7 - Akinetic mutism ROC.png", as(png) name("Graph") replace

report_ci howard_lees

*Central respiratory irregularities

roctab case resp_abn if v_def==1, binomial detail summary table graph

graph save "Graph" "${ROOT}005 Graphs\005.8 - Central resp irregularities ROC.gph", replace

graph export "${ROOT}005 Graphs\005.8 - Central resp irregularities ROC.png", as(png) name("Graph") replace

report_ci howard_lees

*Sleep disorder

roctab case sleep if v_def==1, binomial detail summary table graph

graph save "Graph" "${ROOT}005 Graphs\005.9 - Sleep disorder.gph", replace

graph export "${ROOT}005 Graphs\005.9 - Sleep disorder.png", as(png) name("Graph") replace

report_ci howard_lees

**# Assess sensitivity & specificity for EL vs related diagnoses

roctab case howard_lees if v_def==1 & (diagnosis_EL==1 | case==0), binomial detail summary table graph

report_ci howard_lees

roctab case howard_lees if v_def==1 & (diagnosis_EL==0 | case==0), binomial detail summary table graph

report_ci howard_lees

log close

## 013 Interrater reliability.do

log using "${ROOT}004 Log files\013 Interrater reliability.log", replace

**# Number performed by each rater in main dataset

use "${ROOT}002 DTA files\XXX Ready for analysis.dta", clear

tab rater if v_def==1

**# Prepare data

import excel "${ROOT}001 Raw data\A001 Interrater reliability - for analysis.xlsx", sheet("Sheet2") firstrow case(lower) clear

global interrater_vars influenza febrile_illness neoplasia fh_encephalit subacute_onset reduced_consciousness stupor agitation headache nausea vomiting hypersalivation sleep_reversal insomnia hypersomnia tremor tone_increased bradykinesia tics chorea myoclonus ataxia weakness_general weakness_focal masked_facies reduced_spont_mov akinetic_mutism catalepsy waxy_flex posturing mannerisms stereotypies grimacing echopraxia dyskinesia somatosens_sx euphoria depression lability confusion memory_impairment bradyphrenia mute echolalia pressure_of_speech palilalia verbigeration negativism fatigue impulsivity hypersexuality obs_compuls self_harm personality_change hallucinations delusions overweight underweight appetite_increase intake_reduced polydipsia i ii iii_iv_vi ophthalmoplegia v vii viii ix x xi xii vision_blurred staring seizure seizure_unexplained temp_high temp_low temp_fluct hr_high hr_low hr_fluct central_hypovent central_resp_abn

save "${ROOT}002 DTA files\C001 Interrater reliability.dta", replace

//Rename and label variables

use "${ROOT}002 DTA files\C001 Interrater reliability.dta", clear

rename year year_volume

label variable year_volume "Year of the volume in which casenotes found"

label variable initials "Patient initials"

label variable rater "Person who did data extraction"

rename diagnosispatientnotes diagnosis_notes

label variable diagnosis_notes "Diagnosis given on front sheet of Pt notes"

rename admissiondate admi_date

rename dischargedate disc_date

rename duration illness_duration

label variable illness_duration "Time since start of neuro(psychiatric) Sx of present illness in days"

rename maritalstatus marital_status

rename numberofepisodes episode_number

label variable episode_number "Number of distinct episodes"

rename subacuteonset subacute_onset

rename toneincreased tone_increased

rename febrileillnesstemporallyrelat febrile_illness

rename febrileillnessdate febrile_illness_date

rename neoplasiatype neoplasia_type

rename fhencephalitis fh_encephalitis

rename reducedconsciousness reduced_consciousness

label variable reduced_consciousness "Reduced consciousness"

label variable stupor "Stupor"

label variable agitation "Agitation"

label variable headache "Headache"

label variable nausea "Nausea"

label variable vomiting "Vomiting"

label variable hypersalivation "Hypersalivation"

rename sleepwakecyclereversal sleep_reversal

label variable sleep_reversal "Sleep-wake cycle reversal"

label variable insomnia "Insomnia"

label variable hypersomnia "Hypersomnia"

label variable tremor "Tremor"

label variable tone_increased "Increased tone"

label variable bradykinesia "Bradykinesia"

label variable tics "Tics"

label variable chorea "Chorea"

label variable myoclonus "Myoclonus"

label variable ataxia "Ataxia"

rename generalisedweakness weakness_general

label variable weakness_general "Generalised weakness"

rename focalweakness weakness_focal

label variable weakness_focal "Focal weakness"

rename maskedfacies masked_facies

label variable masked_facies "Masked facies"

rename reductioninspontaneousmovemen reduced_spont_mov

label variable reduced_spont_mov "Reduction in spontaneous movement"

rename akineticmutism akinetic_mutism

label variable akinetic_mutism "Akinetic mutism"

label variable catalepsy "Catalepsy"

rename waxyflexibility waxy_flex

label variable waxy_flex "Waxy flexibility"

label variable posturing "Posturing"

label variable mannerisms "Mannerisms"

label variable stereotypies "Stereotypies"

label variable grimacing "Grimacing"

label variable echopraxia "Echopraxia"

label variable dyskinesia "Dyskinesia"

rename somatosensorysymptoms somatosens_sx

label variable somatosens_sx "Somatosensory symptoms"

label variable euphoria "Euphoria"

label variable depression "Depression"

rename emotionallability lability

label variable lability "Emotional lability"

label variable confusion "Confusion"

rename memoryimpairment memory_impairment

label variable memory_impairment "Memory impairment"

label variable bradyphrenia "Bradyphrenia"

rename mutismorsignificantverbalre mute

label variable mute "Mutism"

label variable echolalia "Echolalia"

rename pressureofspeech pressure_of_speech

label variable pressure_of_speech "Pressure of speech"

label variable palilalia "Palilalia"

label variable verbigeration "Verbigeration"

label variable negativism "Negativism"

label variable fatigue "Fatigue"

label variable impulsivity "Impulsivity"

label variable hypersexuality "Hypersexuality"

rename obsessivecompulsivebehaviour obs_compuls

label variable obs_compuls "Obsessive-compulsive behaviour"

rename selfharming self_harm

label variable self_harm "Self-harm"

rename personalitychange personality_change

label variable personality_change "Personality change"

label variable hallucinations "Hallucination"

label variable delusions "Delusion"

label variable overweight "Overweight"

label variable underweight "Underweight"

rename increasedappetite appetite_increase

label variable appetite_increase "Increased appetite"

rename reducedoralintake intake_reduced

label variable intake_reduced "Reduced oral intake"

label variable polydipsia "Polydipsia"

rename iiiivvi iii_iv_vi

label variable i "I"

label variable ii "II"

label variable iii_iv_vi "III, IV, VI"

label variable v "V"

label variable vii "VII"

label variable viii "VIII"

label variable ix "IX"

label variable x "X"

label variable xi "XI"

label variable xii "XII"

rename blurredvisionordoublevision vision_blurred

label variable vision_blurred "Blurred or double vision"

rename seizurenotexplainedbypreviou seizure_unexplained

rename hightemperature99fwithin5 temp_high

label variable temp_high "Hyperthermia"

rename lowtemperature97fwithin5 temp_low

label variable temp_low "Hypothermia"

rename fluctuatingtemperaturebothlo temp_fluct

label variable temp_fluct "Fluctuating temperature"

rename highhr100within5daysofa hr_high

label variable hr_high "Tachycardia"

rename lowhr60within5daysofadm hr_low

label variable hr_low "Bradycardia"

rename fluctuatinghrchangeof30bpm hr_fluct

label variable hr_fluct "Fluctuating heart rate"

rename centralhypoventilation central_hypovent

rename othercentralrespiratoryirregu central_resp_abn

rename subjectivelyel subjective_EL

rename occupation occupation_raw

label variable occupation_raw "Raw occupation data"

//Sort out inconsistent data

replace tics = "1" if substr(tics,1,1)=="1"

replace myoclonus = "1" if substr(myoclonus,1,1)=="1"

replace myoclonus = "1" if myoclonus == "(twitching?)"

replace grimacing = "1" if substr(grimacing,1,1)=="1"

replace hypersexuality = "1" if substr(hypersexuality,1,1)=="1"

replace personality_change = "1" if substr(personality_change,1,1)=="1"

replace i = "0" if substr(i,1,1)=="n"

replace vii = "1" if substr(vii,1,1)=="1"

replace hr_low = "1" if substr(hr_low,1,1)=="1"

//Ensure correct variable types

describe

encode sex, gen (sex1)

drop sex

rename sex1 sex

tab marital_status, m

encode marital_status, gen(marital)

drop marital_status

tab handedness

encode handedness, gen (handed)

drop handedness

encode rater, gen(rater1)

drop rater

rename rater1 rater

foreach var of varlist tics myoclonus grimacing hypersexuality personality_change i vii hr_low {

gen `var'1 = real(`var')

drop `var'

rename `var'1 `var'

}

//Replace missing data with zero, where appropriate

*This is legitimate, as we are attributing error to misclassification

recode $interrater_vars (. = 0)

//Kappa doesn't work if all numbers for a variable are the same

*Drop all variables without any variability

foreach var of varlist $interrater_vars {

sum `var'

di r(sum)

local var_sum = r(sum)

if `var_sum'==0 drop `var'

}

*Create new macro for these variables

global interrater_vars influenza febrile_illness subacute_onset reduced_consciousness stupor agitation headache hypersalivation sleep_reversal insomnia hypersomnia tremor tone_increased bradykinesia tics chorea myoclonus weakness_general weakness_focal masked_facies reduced_spont_mov waxy_flex mannerisms stereotypies grimacing dyskinesia depression confusion memory_impairment bradyphrenia mute fatigue hypersexuality obs_compuls personality_change overweight underweight ii iii_iv_vi ophthalmoplegia v vii ix x xi xii vision_blurred staring seizure seizure_unexplained temp_high temp_low hr_high hr_low hr_fluct central_resp_abn

desc $interrater_var

save "${ROOT}002 DTA files\C002 Interrater reliability - data cleaned.dta", replace

//Reshape data for kappa considering all results to be from the same measure

use "${ROOT}002 DTA files\C002 Interrater reliability - data cleaned.dta", clear

*Drop -iii_iv_vi-, as it stops the programme working - the variable has been changed already apparently

global interrater_vars influenza febrile_illness subacute_onset reduced_consciousness stupor agitation headache hypersalivation sleep_reversal insomnia hypersomnia tremor tone_increased bradykinesia tics chorea myoclonus weakness_general weakness_focal masked_facies reduced_spont_mov waxy_flex mannerisms stereotypies grimacing dyskinesia depression confusion memory_impairment bradyphrenia mute fatigue hypersexuality obs_compuls personality_change overweight underweight ii ophthalmoplegia v vii ix x xi xii vision_blurred staring seizure seizure_unexplained temp_high temp_low hr_high hr_low hr_fluct central_resp_abn

*Rename measures

global count = 0

foreach var of varlist $interrater_vars {

global count = $count+1

rename `var' measure$count

}

rename measure* measure*_

*Reshape

keep id rater measure*

reshape long measure, i(id rater) j(measure_num) string

tostring id, replace

replace id = id + measure_num

drop measure_num

reshape wide measure, i(id) j(rater)

*Calculate kappa considering all results to be from the same measure

kap measure1 measure2 measure3 measure4

log close

## 014 CSF.do

log using "${ROOT}004 Log files\014 CSF.log", replace

use "${ROOT}002 DTA files\XXX Ready for analysis.dta", clear

**# Cohort numbers

tab csf case if v_def==1

**# Descriptive statistics

table csf_appear if case==1 & v_def==1, statistic(frequency) statistic(percent)

sum csf_cells if case==1 & v_def==1, detail

table csf_cells_high if case==1 & v_def==1, statistic(frequency) statistic(percent)

table csf_cell_type_white if case==1 & v_def==1, statistic(frequency) statistic(percent)

table csf_pleocytosis if case==1 & v_def==1, statistic(frequency) statistic(percent)

sum csf_albumin if case==1 & v_def==1, detail

sum csf_protein if case==1 & v_def==1, detail

table csf_nonne if case==1 & v_def==1, statistic(frequency) statistic(percent)

table csf_pandy if case==1 & v_def==1, statistic(frequency) statistic(percent)

table csf_lange_interp if case==1 & v_def==1, statistic(frequency) statistic(percent)

log close

# Supplementary Table 4: R code

# Setup -------------------------------------------------------------------

setwd("G:/My Drive/Research/Encephalitis lethargica QS series/R")

library(tidyverse)

library(haven)

library(ggplot2)

library(RColorBrewer)

# Import data -------------------------------------------------------------

el.df <- read_dta("G:/My Drive/Research/Encephalitis lethargica QS series/Stata/002 DTA files/XXX Ready for analysis.dta")

# Summarise data ----------------------------------------------------------

# Drop all ineligible rows

el.df = subset(el.df, case==1 & v_def==1)

# Create summary dataframe

el.sum <- data.frame(

clin_feat = c("reduced consciousness", "stupor", "agitation", "nausea", "vomiting", "hypersalivation", "overweight", "underweight",

"appetite increased",

"oral intake reduced", "polydipsia", "sleep reversal", "insomnia", "hypersomnia", "parasomnia", "tremor", "tone increased",

"bradykinesia", "tics", "chorea", "myoclonus", "ataxia", "generalised weakness", "focal weakness", "masked facies",

"reduced spontaneous movement", "akinetic mutism", "catalepsy", "waxy flexibility", "posturing", "mannerisms", "stereotypies",

"grimacing", "echopraxia", "dyskinesia", "dystonia", "confusion", "memory impairment", "bradyphrenia", "disorientation",

"dysphasia", "impaired attention", "impaired numeric function", "nonspecific cognitive impairment", "perseveration",

"rldisorientation", "subjective cognitive impairment", "anomia", "mutism", "echolalia", "pressure of speech", "palilalia",

"verbigeration", "oligophasia", "bradyphasia", "tachyphasia", "hypophonia", "monotonous speech",

"dysarthria", "high-pitched speech", "low-pitched speech", "stammer", "very deliberate speech", "speech, miscellaneous",

"euphoria", "depression", "mood lability", "negativism", "fatigue", "impulsivity", "unacceptable behaviour",

"hypersexuality",

"obsessive-compulsive symptoms", "self-harm", "personality change", "hallucinations", "delusions", "anxiety", "apathy",

"depersonalisation-derealisation", "blunted affect", "dissociation", "reduced libido", "psychiatric, miscellaneous",

"I", "II", "III, IV, VI", "V", "VII", "VIII", "IX", "X", "XI", "XII", "ophthalmoplegia", "oculogyric crisis",

"blurred vision", "staring", "high temperature", "low temperature", "temperature fluctuation", "high heart rate", "low heart rate",

"heart rate fluctuation", "central hypoventilation", "central respiratory abnormality", "headache",

"somatosensory symptoms", "seizure", "seizure, unexplained"),

sum = c(sum(el.df$reduced_consciousness), sum(el.df$stupor), sum(el.df$agitation), sum(el.df$nausea), sum(el.df$vomiting),

sum(el.df$hypersalivation), sum(el.df$overweight), sum(el.df$underweight), sum(el.df$appetite_increase),

sum(el.df$intake_reduced), sum(el.df$polydipsia), sum(el.df$sleep_reversal), sum(el.df$insomnia), sum(el.df$hypersomnia),

sum(el.df$parasomnia), sum(el.df$tremor), sum(el.df$tone_increased), sum(el.df$bradykinesia), sum(el.df$tics),

sum(el.df$chorea), sum(el.df$myoclonus), sum(el.df$ataxia), sum(el.df$weakness_general), sum(el.df$weakness_focal),

sum(el.df$masked_facies), sum(el.df$reduced_spont_mov), sum(el.df$akinetic_mutism), sum(el.df$catalepsy),

sum(el.df$waxy_flex), sum(el.df$posturing), sum(el.df$mannerisms), sum(el.df$stereotypies), sum(el.df$grimacing),

sum(el.df$echopraxia), sum(el.df$dyskinesia), sum(el.df$dystonia), sum(el.df$confusion), sum(el.df$memory_impairment),

sum(el.df$bradyphrenia), sum(el.df$disorientation), sum(el.df$dysphasia), sum(el.df$impairedattention),

sum(el.df$impairednumericfunction), sum(el.df$nonspecificcognitiveimpairmen), sum(el.df$perseveration),

sum(el.df$rldisorientation), sum(el.df$subjectivecognitiveimpairment), sum(el.df$anomia), sum(el.df$mute),

sum(el.df$echolalia), sum(el.df$pressure_of_speech), sum(el.df$palilalia), sum(el.df$verbigeration),

sum(el.df$oligophasia), sum(el.df$bradyphasia), sum(el.df$tachyphasia), sum(el.df$quietspeech), sum(el.df$monotonous_speech),

sum(el.df$dysarthricindistinctspeech), sum(el.df$highpitchedspeech), sum(el.df$lowpitched), sum(el.df$stammer),

sum(el.df$verydeliberate), sum(el.df$speech_miscellaneous), sum(el.df$euphoria), sum(el.df$depression),

sum(el.df$lability), sum(el.df$negativism), sum(el.df$fatigue), sum(el.df$impulsivity), sum(el.df$unaccept_behav),

sum(el.df$hypersexuality), sum(el.df$obs_compuls), sum(el.df$self_harm), sum(el.df$personality_change),

sum(el.df$hallucinations), sum(el.df$delusions), sum(el.df$anxiety), sum(el.df$apathy),

sum(el.df$depersonalisationderealisation), sum(el.df$affect_blunt), sum(el.df$dissociation), sum(el.df$reduced_libido),

sum(el.df$psych_misc), sum(el.df$i), sum(el.df$ii), sum(el.df$iii_iv_vi), sum(el.df$v), sum(el.df$vii), sum(el.df$viii),

sum(el.df$ix), sum(el.df$x), sum(el.df$xi), sum(el.df$xii), sum(el.df$ophthalmoplegia), sum(el.df$oculogyric_crisis),

sum(el.df$vision_blurred), sum(el.df$staring), sum(el.df$temp_high), sum(el.df$temp_low), sum(el.df$temp_fluct),

sum(el.df$hr_high), sum(el.df$hr_low), sum(el.df$hr_fluct), sum(el.df$central_hypovent), sum(el.df$central_resp_abn),

sum(el.df$headache), sum(el.df$somatosens_sx), sum(el.df$seizure), sum(el.df$seizure_unexplained))

)

# Remove clinical features ------------------------------------------------

# Remove features with < 10 results

el.sum <- el.sum[el.sum$sum >= 10, ]

# Group the clinical features ---------------------------------------------

# Create a named vector with the mapping of 'clin_feat' values to 'group' values

group_mapping <- c(

"reduced consciousness" = "arousal level",

"stupor" = "arousal level",

"agitation" = "arousal level",

"nausea" = "gastrointestinal",

"vomiting" = "gastrointestinal",

"hypersalivation" = "gastrointestinal",

"overweight" = "gastrointestinal",

"underweight" = "gastrointestinal",

"appetite increased" = "gastrointestinal",

"oral intake reduced" = "gastrointestinal",

"polydipsia" = "gastrointestinal",

"sleep reversal" = "sleep",

"insomnia" = "sleep",

"hypersomnia" = "sleep",

"parasomnia" = "sleep",

"tremor" = "motor",

"tone increased" = "motor",

"bradykinesia" = "motor",

"tics" = "motor",

"chorea" = "motor",

"myoclonus" = "motor",

"ataxia" = "motor",

"generalised weakness" = "motor",

"focal weakness" = "motor",

"masked facies" = "motor",

"reduced spontaneous movement" = "motor",

"akinetic mutism" = "motor",

"catalepsy" = "motor",

"waxy flexibility" = "motor",

"posturing" = "motor",

"mannerisms" = "motor",

"stereotypies" = "motor",

"grimacing" = "motor",

"echopraxia" = "motor",

"dyskinesia" = "motor",

"dystonia" = "motor",

"confusion" = "cognitive",

"memory impairment" = "cognitive",

"bradyphrenia" = "cognitive",

"disorientation" = "cognitive",

"dysphasia" = "cognitive",

"impaired attention" = "cognitive",

"impaired numeric function" = "cognitive",

"nonspecific cognitive impairment" = "cognitive",

"perseveration" = "cognitive",

"rldisorientation" = "cognitive",

"subjective cognitive impairment" = "cognitive",

"anomia" = "cognitive",

"mutism" = "speech",

"echolalia" = "speech",

"pressure of speech" = "speech",

"palilalia" = "speech",

"verbigeration" = "speech",

"oligophasia" = "speech",

"bradyphasia" = "speech",

"tachyphasia" = "speech",

"hypophonia" = "speech",

"monotonous speech" = "speech",

"dysarthria" = "speech",

"high-pitched speech" = "speech",

"low-pitched speech" = "speech",

"stammer" = "speech",

"very deliberate speech" = "speech",

"speech, miscellaneous" = "speech",

"euphoria" = "psychiatric",

"depression" = "psychiatric",

"mood lability" = "psychiatric",

"negativism" = "psychiatric",

"fatigue" = "psychiatric",

"impulsivity" = "psychiatric",

"unacceptable behaviour" = "psychiatric",

"hypersexuality" = "psychiatric",

"obsessive-compulsive symptoms" = "psychiatric",

"self-harm" = "psychiatric",

"personality change" = "psychiatric",

"hallucinations" = "psychiatric",

"delusions" = "psychiatric",

"anxiety" = "psychiatric",

"apathy" = "psychiatric",

"depersonalisation-derealisation" = "psychiatric",

"blunted affect" = "psychiatric",

"dissociation" = "psychiatric",

"reduced libido" = "psychiatric",

"psych, miscellaneous" = "psychiatric",

"I" = "cranial nerve",

"II" = "cranial nerve",

"III, IV, VI" = "cranial nerve",

"V" = "cranial nerve",

"VII" = "cranial nerve",

"VIII" = "cranial nerve",

"IX" = "cranial nerve",

"X" = "cranial nerve",

"XI" = "cranial nerve",

"XII" = "cranial nerve",

"ophthalmoplegia" = "ophthalmological",

"oculogyric crisis" = "ophthalmological",

"blurred vision" = "ophthalmological",

"staring" = "ophthalmological",

"high temperature" = "vital signs",

"low temperature" = "vital signs",

"temperature fluctuation" = "vital signs",

"high heart rate" = "vital signs",

"low heart rate" = "vital signs",

"heart rate fluctuation" = "vital signs",

"central hypoventilation" = "vital signs",

"central respiratory abnormality" = "vital signs",

"headache" = "neurological, other",

"somatosensory symptoms" = "neurological, other",

"seizure" = "neurological, other",

"seizure, unexplained" = "neurological, other"

)

# Add the 'group' column based on the mapping

el.sum$group <- group_mapping[el.sum$clin_feat]

# Sort by group

custom_order <- c('arousal level', 'cognitive', 'motor', 'ophthalmological', 'gastrointestinal', 'neurological, other', 'cranial nerve', 'psychiatric', 'sleep', 'speech', 'vital signs')

el.sum$group <- factor(el.sum$group, levels = custom_order)

el.sum <- el.sum[order(el.sum$group, el.sum$sum), ]

el.sum = cbind(id = seq_len(nrow(el.sum)), el.sum)

# Code adapted from: https://r-graph-gallery.com/circular-barplot.html

# Graph setup -------------------------------------------------------------

# Prepare labels ----------------------------------------------------------

# create % column

el.sum$percent = el.sum$sum / 614 * 100

el.sum$percent <- format(round(el.sum$percent, 1), nsmall = 1)

# make label text

el.sum$lab_text <- paste(el.sum$clin_feat, ", ", el.sum$sum,

" (", trimws(el.sum$percent), "%)", sep = "")

# calculate the ANGLE of the labels

number_of_bar <- nrow(el.sum)

angle <- 90 - 360 * (el.sum$id-0.5) /number_of_bar # I substract 0.5 because the letter must have the angle of the center of the bars. Not extreme right(1) or extreme left (0)

# calculate the alignment of labels: right or left

# If I am on the left part of the plot, my labels have currently an angle < -90

el.sum$hjust<-ifelse( angle < -90, 1, 0)

# flip angle BY to make them readable

el.sum$angle<-ifelse(angle < -90, angle+180, angle)

# Make graph --------------------------------------------------------------

# Make the plot

p <- ggplot(el.sum, aes(x=as.factor(id), y=sum, fill=group)) + # Note that id is a factor. If x is numeric, there is some space between the first bar

# This adds the bars with a blue color

geom_bar(stat="identity", alpha=0.5) +

# Limits of the plot = very important. The negative value controls the size of the inner circle, the positive one is useful to add size over each bar

ylim(-200,614) +

# Customise the theme: no axis title and no cartesian grid

theme_classic() +

theme(

axis.text = element_blank(),

axis.title = element_blank(),

panel.grid = element_blank(),

plot.margin = unit(rep(0,5), "cm"), # This removes unnecessary margin around plot

legend.margin= margin(6,50,6,6),

legend.title = element_blank(),

legend.key.size = unit(0.5, "lines"),

legend.text = element_text(size=5)

) +

# This makes the coordinate polar instead of cartesian.

coord_polar() +

# Add the labels, using the label_data dataframe that we have created before

geom_text(data=el.sum, aes(x=id, y=sum+25, label=lab_text, hjust=hjust),

color="black", fontface="bold",alpha=0.6, size=1.4, angle= el.sum$angle, inherit.aes = FALSE ) +

# Replace green color with another color

scale_fill_manual(values = c("pink", "blue", "gold", "orange", "purple",

"brown", "cyan", "black", "gray", "magenta", "yellow"))

p

ggsave("./005 Graphs/003 Circular bar of clinical features.png", dpi=500, bg="white")

# Make summary graph for graphical abstract -------------------------------

## Import data -------------------------------------------------------------

el.df <- read_dta("G:/My Drive/Research/Encephalitis lethargica QS series/Stata/002 DTA files/XXX Ready for analysis.dta")

## Summarise data ----------------------------------------------------------

# Drop all ineligible rows

el.df = subset(el.df, case==1 & v_def==1)

# Create summary dataframe

el.sum <- data.frame(

clin_feat = c("reduced consciousness", "stupor", "agitation", "nausea", "vomiting", "hypersalivation", "overweight", "underweight",

"appetite increased",

"oral intake reduced", "polydipsia", "sleep reversal", "insomnia", "hypersomnia", "parasomnia", "tremor", "tone increased",

"bradykinesia", "tics", "chorea", "myoclonus", "ataxia", "generalised weakness", "focal weakness", "masked facies",

"reduced spontaneous movement", "akinetic mutism", "catalepsy", "waxy flexibility", "posturing", "mannerisms", "stereotypies",

"grimacing", "echopraxia", "dyskinesia", "dystonia", "confusion", "memory impairment", "bradyphrenia", "disorientation",

"dysphasia", "impaired attention", "impaired numeric function", "nonspecific cognitive impairment", "perseveration",

"rldisorientation", "subjective cognitive impairment", "anomia", "mutism", "echolalia", "pressure of speech", "palilalia",

"verbigeration", "oligophasia", "bradyphasia", "tachyphasia", "hypophonia", "monotonous speech",

"dysarthria", "high-pitched speech", "low-pitched speech", "stammer", "very deliberate speech", "speech, miscellaneous",

"euphoria", "depression", "mood lability", "negativism", "fatigue", "impulsivity", "unacceptable behaviour",

"hypersexuality",

"obsessive-compulsive symptoms", "self-harm", "personality change", "hallucinations", "delusions", "anxiety", "apathy",

"depersonalisation-derealisation", "blunted affect", "dissociation", "reduced libido", "psychiatric, miscellaneous",

"I", "II", "cranial nerve III, IV, VI", "V", "cranial nerve VII", "VIII", "IX", "X", "XI", "cranial nerve XII", "ophthalmoplegia", "oculogyric crisis",

"blurred vision", "staring", "high temperature", "low temperature", "temperature fluctuation", "tachycardia", "low heart rate",

"heart rate fluctuation", "central hypoventilation", "central respiratory abnormality", "headache",

"somatosensory symptoms", "seizure", "seizure, unexplained"),

sum = c(sum(el.df$reduced_consciousness), sum(el.df$stupor), sum(el.df$agitation), sum(el.df$nausea), sum(el.df$vomiting),

sum(el.df$hypersalivation), sum(el.df$overweight), sum(el.df$underweight), sum(el.df$appetite_increase),

sum(el.df$intake_reduced), sum(el.df$polydipsia), sum(el.df$sleep_reversal), sum(el.df$insomnia), sum(el.df$hypersomnia),

sum(el.df$parasomnia), sum(el.df$tremor), sum(el.df$tone_increased), sum(el.df$bradykinesia), sum(el.df$tics),

sum(el.df$chorea), sum(el.df$myoclonus), sum(el.df$ataxia), sum(el.df$weakness_general), sum(el.df$weakness_focal),

sum(el.df$masked_facies), sum(el.df$reduced_spont_mov), sum(el.df$akinetic_mutism), sum(el.df$catalepsy),

sum(el.df$waxy_flex), sum(el.df$posturing), sum(el.df$mannerisms), sum(el.df$stereotypies), sum(el.df$grimacing),

sum(el.df$echopraxia), sum(el.df$dyskinesia), sum(el.df$dystonia), sum(el.df$confusion), sum(el.df$memory_impairment),

sum(el.df$bradyphrenia), sum(el.df$disorientation), sum(el.df$dysphasia), sum(el.df$impairedattention),

sum(el.df$impairednumericfunction), sum(el.df$nonspecificcognitiveimpairmen), sum(el.df$perseveration),

sum(el.df$rldisorientation), sum(el.df$subjectivecognitiveimpairment), sum(el.df$anomia), sum(el.df$mute),

sum(el.df$echolalia), sum(el.df$pressure_of_speech), sum(el.df$palilalia), sum(el.df$verbigeration),

sum(el.df$oligophasia), sum(el.df$bradyphasia), sum(el.df$tachyphasia), sum(el.df$quietspeech), sum(el.df$monotonous_speech),

sum(el.df$dysarthricindistinctspeech), sum(el.df$highpitchedspeech), sum(el.df$lowpitched), sum(el.df$stammer),

sum(el.df$verydeliberate), sum(el.df$speech_miscellaneous), sum(el.df$euphoria), sum(el.df$depression),

sum(el.df$lability), sum(el.df$negativism), sum(el.df$fatigue), sum(el.df$impulsivity), sum(el.df$unaccept_behav),

sum(el.df$hypersexuality), sum(el.df$obs_compuls), sum(el.df$self_harm), sum(el.df$personality_change),

sum(el.df$hallucinations), sum(el.df$delusions), sum(el.df$anxiety), sum(el.df$apathy),

sum(el.df$depersonalisationderealisation), sum(el.df$affect_blunt), sum(el.df$dissociation), sum(el.df$reduced_libido),

sum(el.df$psych_misc), sum(el.df$i), sum(el.df$ii), sum(el.df$iii_iv_vi), sum(el.df$v), sum(el.df$vii), sum(el.df$viii),

sum(el.df$ix), sum(el.df$x), sum(el.df$xi), sum(el.df$xii), sum(el.df$ophthalmoplegia), sum(el.df$oculogyric_crisis),

sum(el.df$vision_blurred), sum(el.df$staring), sum(el.df$temp_high), sum(el.df$temp_low), sum(el.df$temp_fluct),

sum(el.df$hr_high), sum(el.df$hr_low), sum(el.df$hr_fluct), sum(el.df$central_hypovent), sum(el.df$central_resp_abn),

sum(el.df$headache), sum(el.df$somatosens_sx), sum(el.df$seizure), sum(el.df$seizure_unexplained))

)

## Remove clinical features ------------------------------------------------

# Remove features with < 10 results

el.sum <- el.sum[el.sum$sum >= 100, ]

## Group the clinical features ---------------------------------------------

# Create a named vector with the mapping of 'clin_feat' values to 'group' values

group_mapping <- c(

"reduced consciousness" = "arousal level",

"stupor" = "arousal level",

"agitation" = "arousal level",

"nausea" = "gastrointestinal",

"vomiting" = "gastrointestinal",

"hypersalivation" = "gastrointestinal",

"overweight" = "gastrointestinal",

"underweight" = "gastrointestinal",

"appetite increased" = "gastrointestinal",

"oral intake reduced" = "gastrointestinal",

"polydipsia" = "gastrointestinal",

"sleep reversal" = "sleep",

"insomnia" = "sleep",

"hypersomnia" = "sleep",

"parasomnia" = "sleep",

"tremor" = "motor",

"tone increased" = "motor",

"bradykinesia" = "motor",

"tics" = "motor",

"chorea" = "motor",

"myoclonus" = "motor",

"ataxia" = "motor",

"generalised weakness" = "motor",

"focal weakness" = "motor",

"masked facies" = "motor",

"reduced spontaneous movement" = "motor",

"akinetic mutism" = "motor",

"catalepsy" = "motor",

"waxy flexibility" = "motor",

"posturing" = "motor",

"mannerisms" = "motor",

"stereotypies" = "motor",

"grimacing" = "motor",

"echopraxia" = "motor",

"dyskinesia" = "motor",

"dystonia" = "motor",

"confusion" = "cognitive",

"memory impairment" = "cognitive",

"bradyphrenia" = "cognitive",

"disorientation" = "cognitive",

"dysphasia" = "cognitive",

"impaired attention" = "cognitive",

"impaired numeric function" = "cognitive",

"nonspecific cognitive impairment" = "cognitive",

"perseveration" = "cognitive",

"rldisorientation" = "cognitive",

"subjective cognitive impairment" = "cognitive",

"anomia" = "cognitive",

"mutism" = "speech",

"echolalia" = "speech",

"pressure of speech" = "speech",

"palilalia" = "speech",

"verbigeration" = "speech",

"oligophasia" = "speech",

"bradyphasia" = "speech",

"tachyphasia" = "speech",

"hypophonia" = "speech",

"monotonous speech" = "speech",

"dysarthria" = "speech",

"high-pitched speech" = "speech",

"low-pitched speech" = "speech",

"stammer" = "speech",

"very deliberate speech" = "speech",

"speech, miscellaneous" = "speech",

"euphoria" = "psychiatric",

"depression" = "psychiatric",

"mood lability" = "psychiatric",

"negativism" = "psychiatric",

"fatigue" = "psychiatric",

"impulsivity" = "psychiatric",

"unacceptable behaviour" = "psychiatric",

"hypersexuality" = "psychiatric",

"obsessive-compulsive symptoms" = "psychiatric",

"self-harm" = "psychiatric",

"personality change" = "psychiatric",

"hallucinations" = "psychiatric",

"delusions" = "psychiatric",

"anxiety" = "psychiatric",

"apathy" = "psychiatric",

"depersonalisation-derealisation" = "psychiatric",

"blunted affect" = "psychiatric",

"dissociation" = "psychiatric",

"reduced libido" = "psychiatric",

"psych, miscellaneous" = "psychiatric",

"I" = "cranial nerve",

"II" = "cranial nerve",

"cranial nerve III, IV, VI" = "cranial nerve",

"V" = "cranial nerve",

"cranial nerve VII" = "cranial nerve",

"VIII" = "cranial nerve",

"IX" = "cranial nerve",

"X" = "cranial nerve",

"XI" = "cranial nerve",

"cranial nerve XII" = "cranial nerve",

"ophthalmoplegia" = "ophthalmological",

"oculogyric crisis" = "ophthalmological",

"blurred vision" = "ophthalmological",

"staring" = "ophthalmological",

"high temperature" = "vital signs",

"low temperature" = "vital signs",

"temperature fluctuation" = "vital signs",

"tachycardia" = "vital signs",

"low heart rate" = "vital signs",

"heart rate fluctuation" = "vital signs",

"central hypoventilation" = "vital signs",

"central respiratory abnormality" = "vital signs",

"headache" = "neurological, other",

"somatosensory symptoms" = "neurological, other",

"seizure" = "neurological, other",

"seizure, unexplained" = "neurological, other"

)

# Add the 'group' column based on the mapping

el.sum$group <- group_mapping[el.sum$clin_feat]

# Sort by group

custom_order <- c('gastrointestinal', 'arousal level', 'cognitive', 'motor', 'ophthalmological', 'cranial nerve', 'neurological, other', 'psychiatric', 'sleep', 'speech', 'vital signs')

el.sum$group <- factor(el.sum$group, levels = custom_order)

el.sum <- el.sum[order(el.sum$group, el.sum$sum), ]

el.sum = cbind(id = seq_len(nrow(el.sum)), el.sum)

# Code adapted from: https://r-graph-gallery.com/circular-barplot.html

## Graph setup -------------------------------------------------------------

## Prepare labels ----------------------------------------------------------

# create % column

el.sum$percent = el.sum$sum / 614 * 100

el.sum$percent <- format(round(el.sum$percent, 1), nsmall = 1)

# make label text

el.sum$lab_text <- paste(el.sum$clin_feat, ", ",

" ", trimws(el.sum$percent), "%", sep = "")

# calculate the ANGLE of the labels

number_of_bar <- nrow(el.sum)

angle <- 90 - 360 * (el.sum$id-0.5) /number_of_bar # I substract 0.5 because the letter must have the angle of the center of the bars. Not extreme right(1) or extreme left (0)

# calculate the alignment of labels: right or left

# If I am on the left part of the plot, my labels have currently an angle < -90

el.sum$hjust<-ifelse( angle < -90, 1, 0)

# flip angle BY to make them readable

el.sum$angle<-ifelse(angle < -90, angle+180, angle)

## Make graph --------------------------------------------------------------

# Make the plot

p <- ggplot(el.sum, aes(x=as.factor(id), y=sum, fill=group)) + # Note that id is a factor. If x is numeric, there is some space between the first bar

# This adds the bars with a blue color

geom_bar(stat="identity", alpha=0.5) +

# Limits of the plot = very important. The negative value controls the size of the inner circle, the positive one is useful to add size over each bar

ylim(-200,1000) +

# Customise the theme: no axis title and no cartesian grid

theme_classic() +

theme(

axis.text = element_blank(),

axis.title = element_blank(),

panel.grid = element_blank(),

) +

# This makes the coordinate polar instead of cartesian.

coord_polar() +

# Add the labels, using the label_data dataframe that we have created before

geom_text(data=el.sum, aes(x=id, y=sum+25, label=lab_text, hjust=hjust),

color="black", fontface="bold",alpha=0.6, size=3, angle= el.sum$angle, inherit.aes = FALSE ) +

guides(fill = 'none')

p

ggsave("./005 Graphs/004 Circular bar of clinical features.png", dpi=500, bg="white")

# Supplementary Table 5: Contingency table for Howard and Lees criteria for encephalitis lethargica

|  | Meets Howard and Lees criteria | Does not meet Howard and Lees criteria | Total |
| --- | --- | --- | --- |
| Case | 175 | 439 | 614 |
| Control | 2 | 63 | 65 |
| Total | 177 | 502 | 679 |

# Supplementary Table 6: Performance of individual items of the Howard and Lees criteria for encephalitis lethargica

| Clinical feature | Sensitivity | Specificity | Area under the receiver operator characteristics curve |
| --- | --- | --- | --- |
| Acute or subacute onset | 66.0% | 63.1% | 0.65 |
| Signs of basal ganglia involvement | 93.0% | 56.9% | 0.75 |
| Oculogyric crisis | 13.2% | 100% | 0.57 |
| Ophthalmoplegia | 47.6% | 89.2% | 0.68 |
| Obsessive-compulsive features | 2.0% | 100% | 0.51 |
| Akinetic mutism | 1.5% | 100% | 0.51 |
| Central respiratory irregularities | 10.8% | 95.4% | 0.53 |
| Sleep disorder | 66.1% | 87.7% | 0.77 |

# Supplementary Figure 1: Example case notes from the first eligible patient admitted


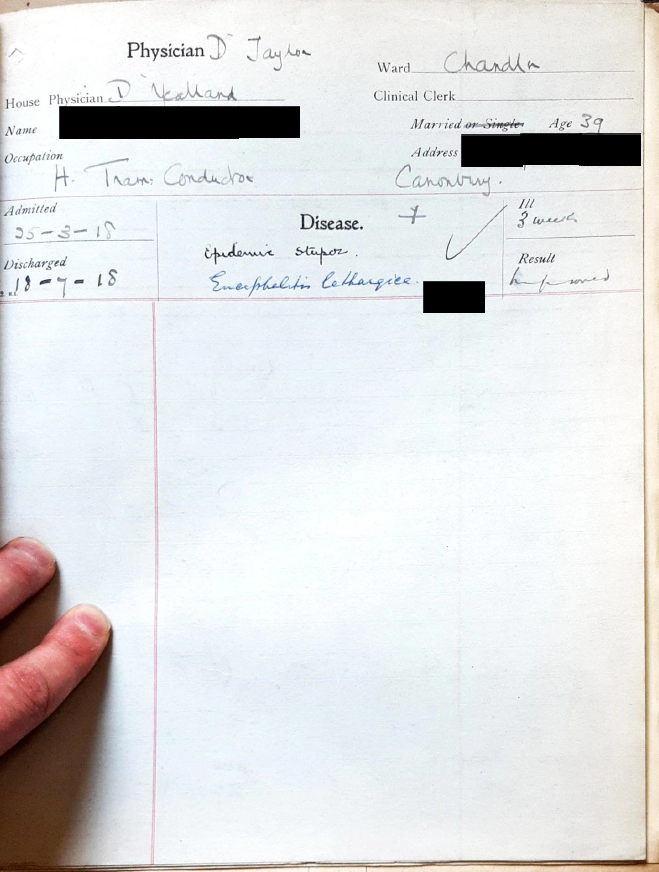


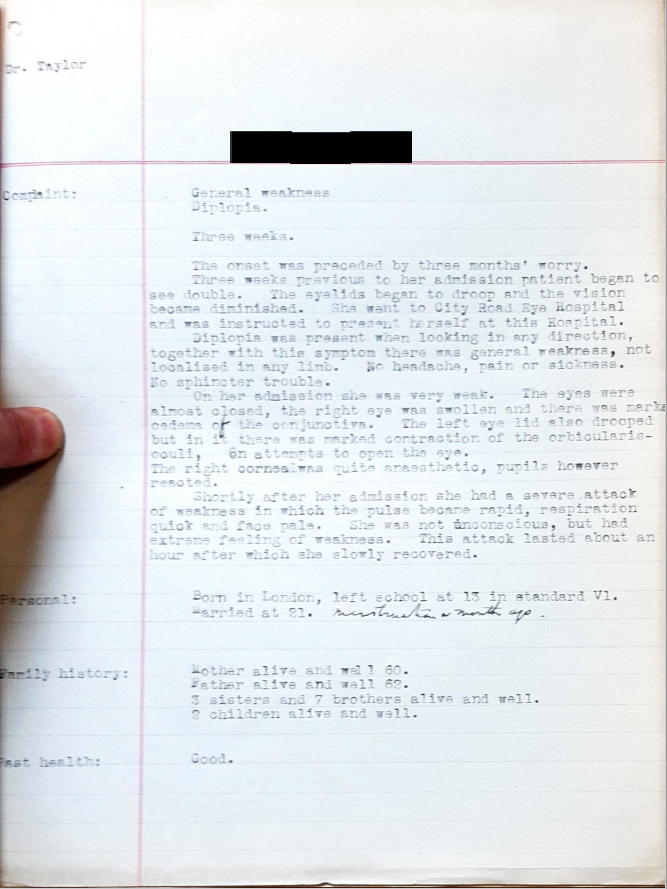


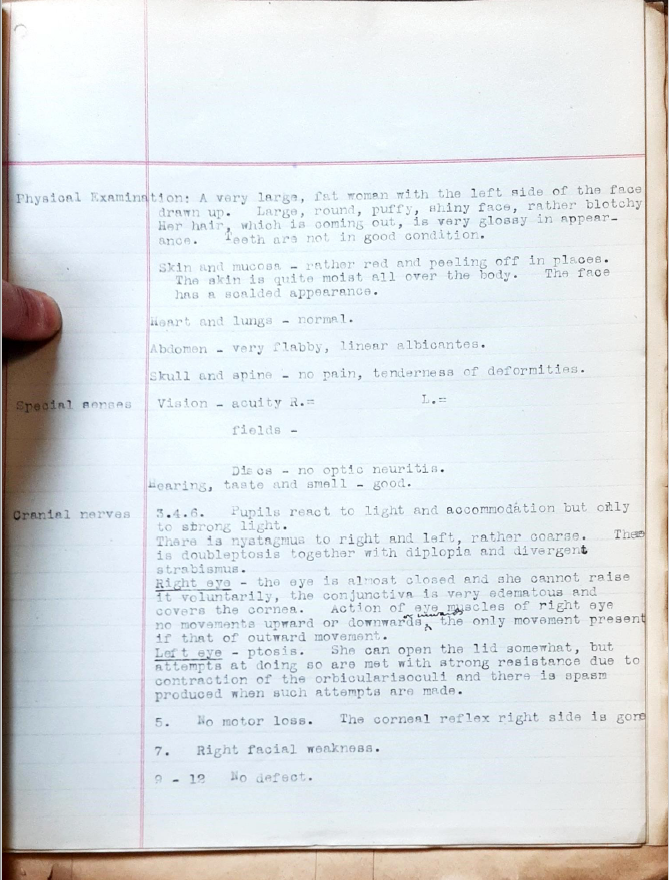


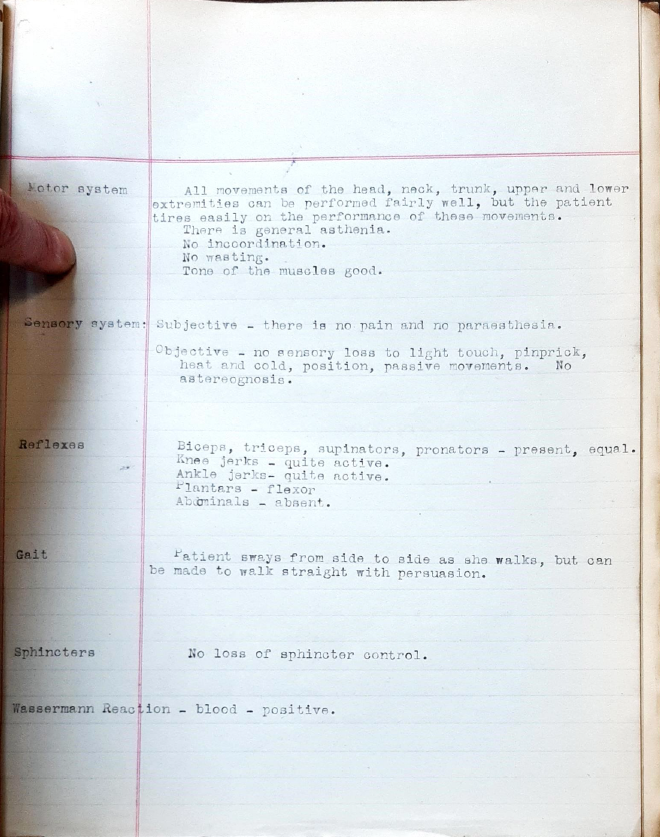


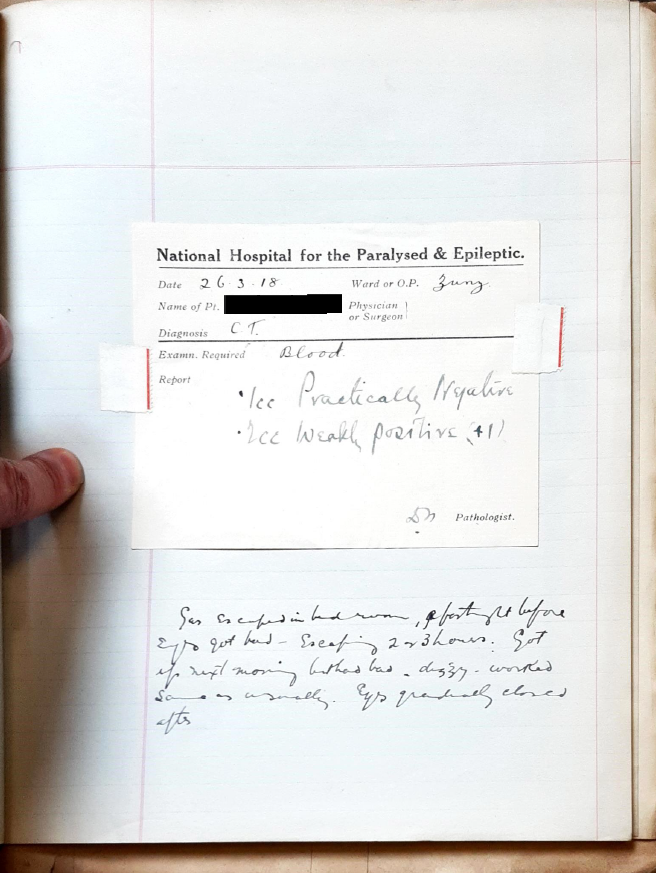


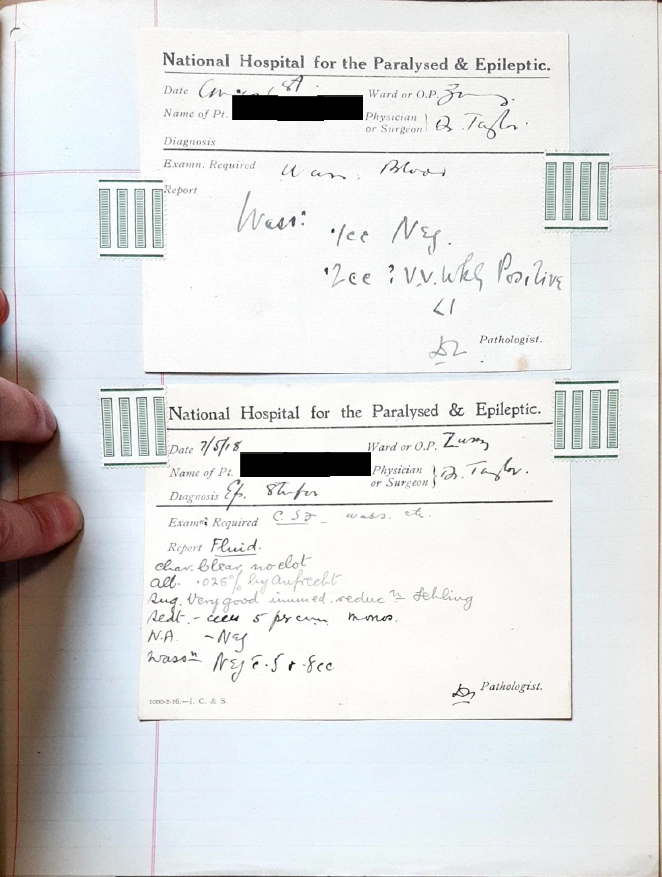


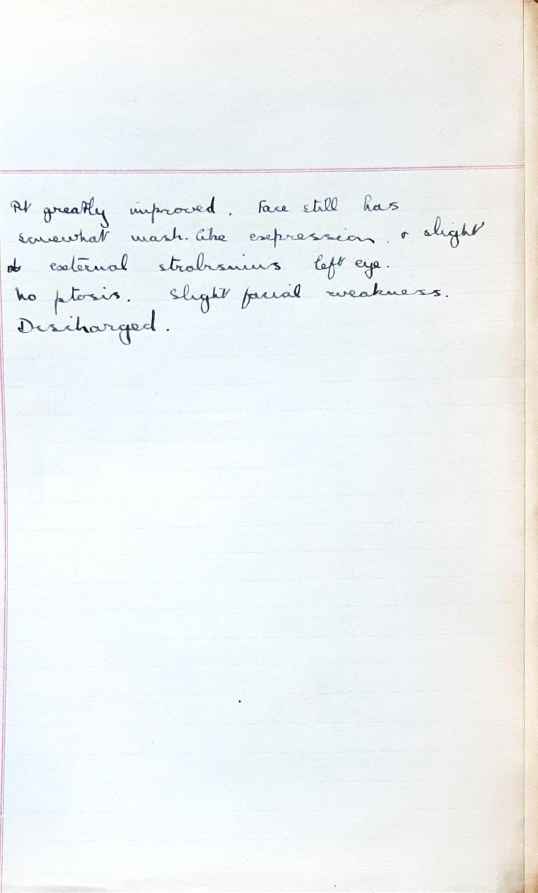


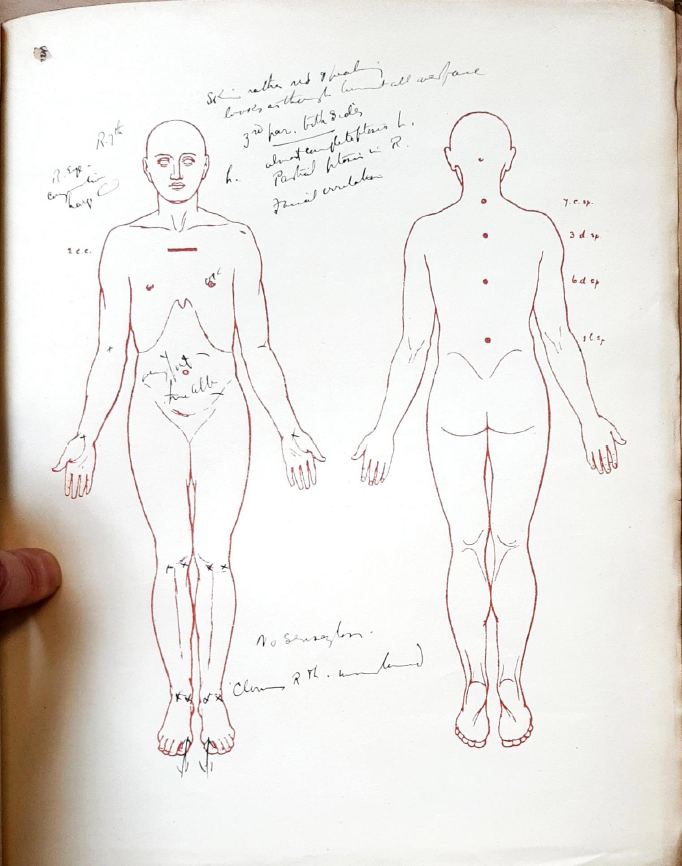


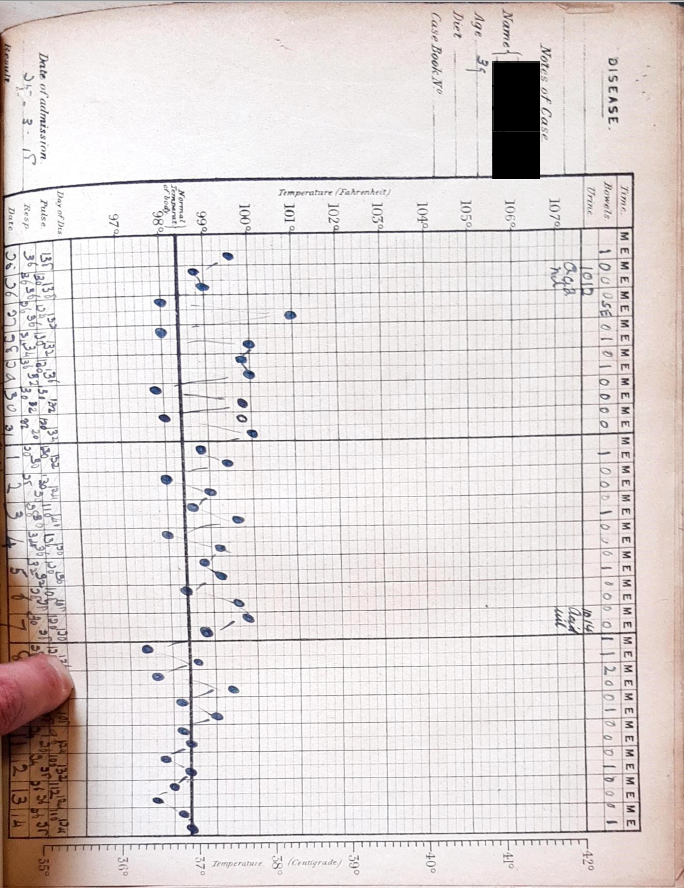


# Supplementary Figure 2: Example case notes from the last eligible patient admitted


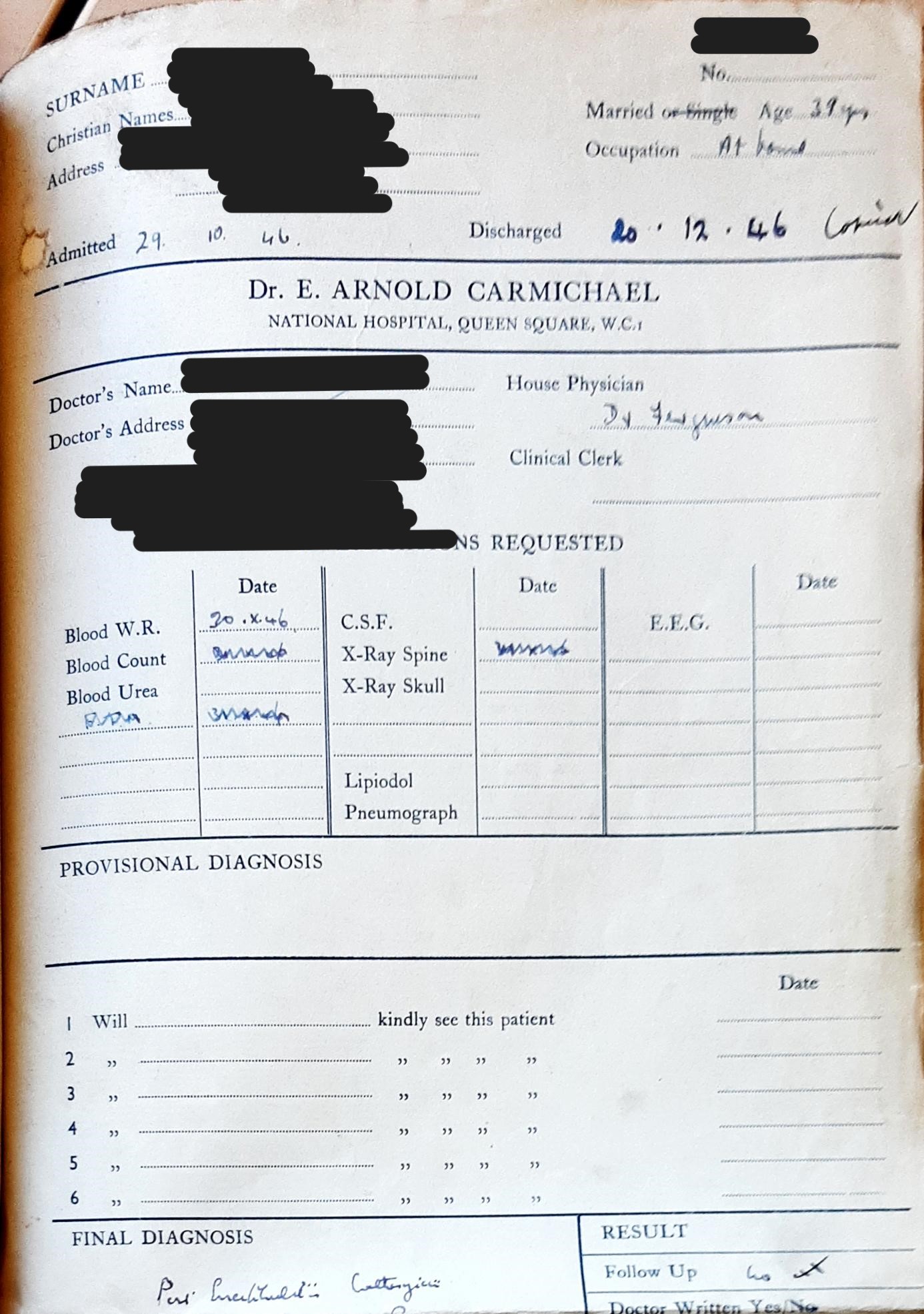


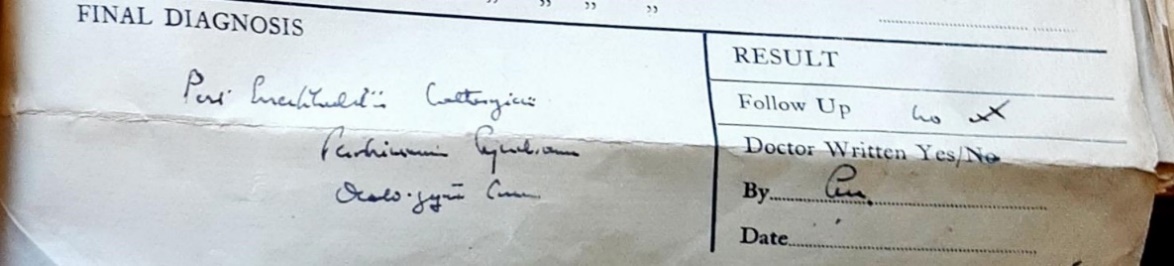


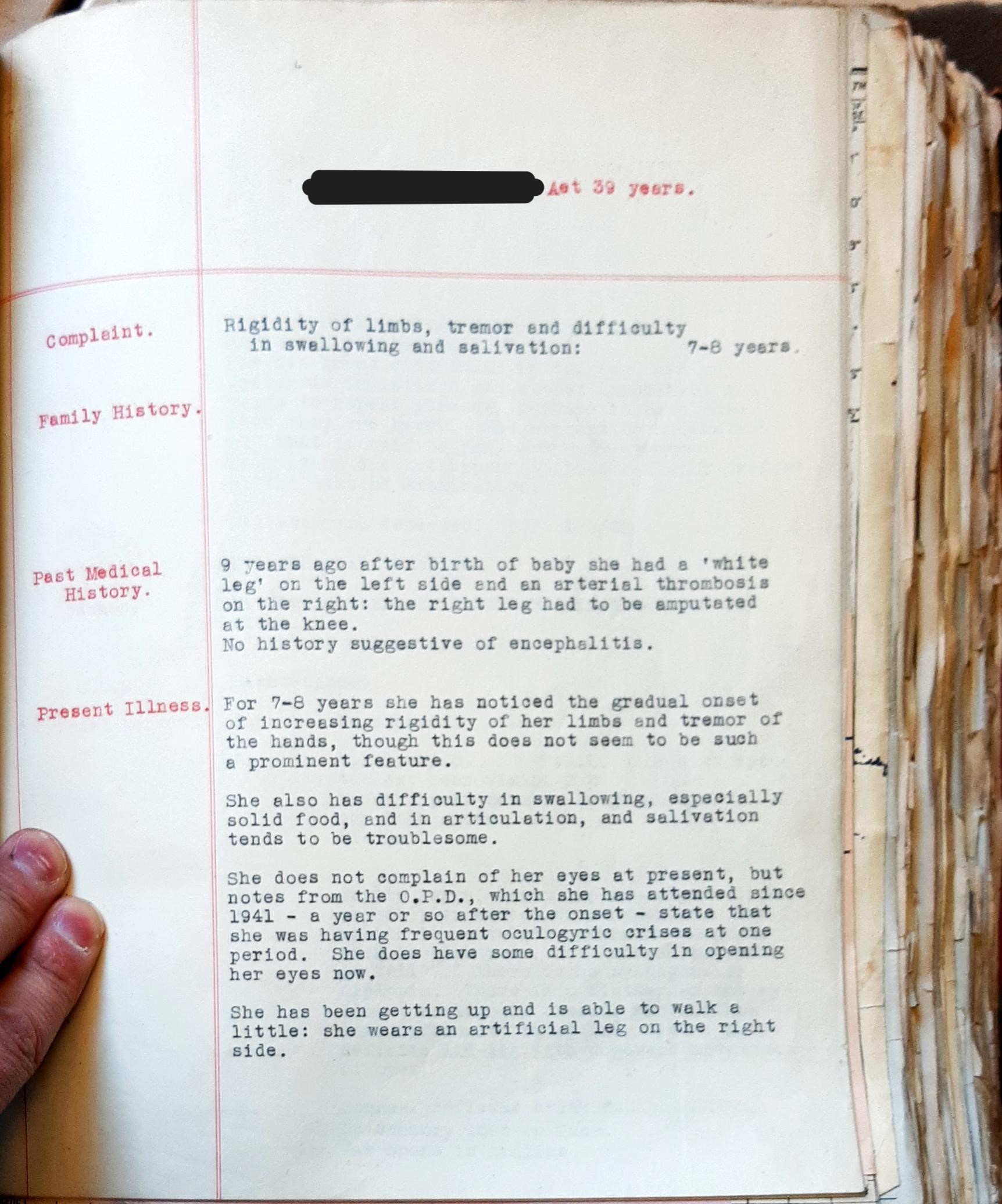


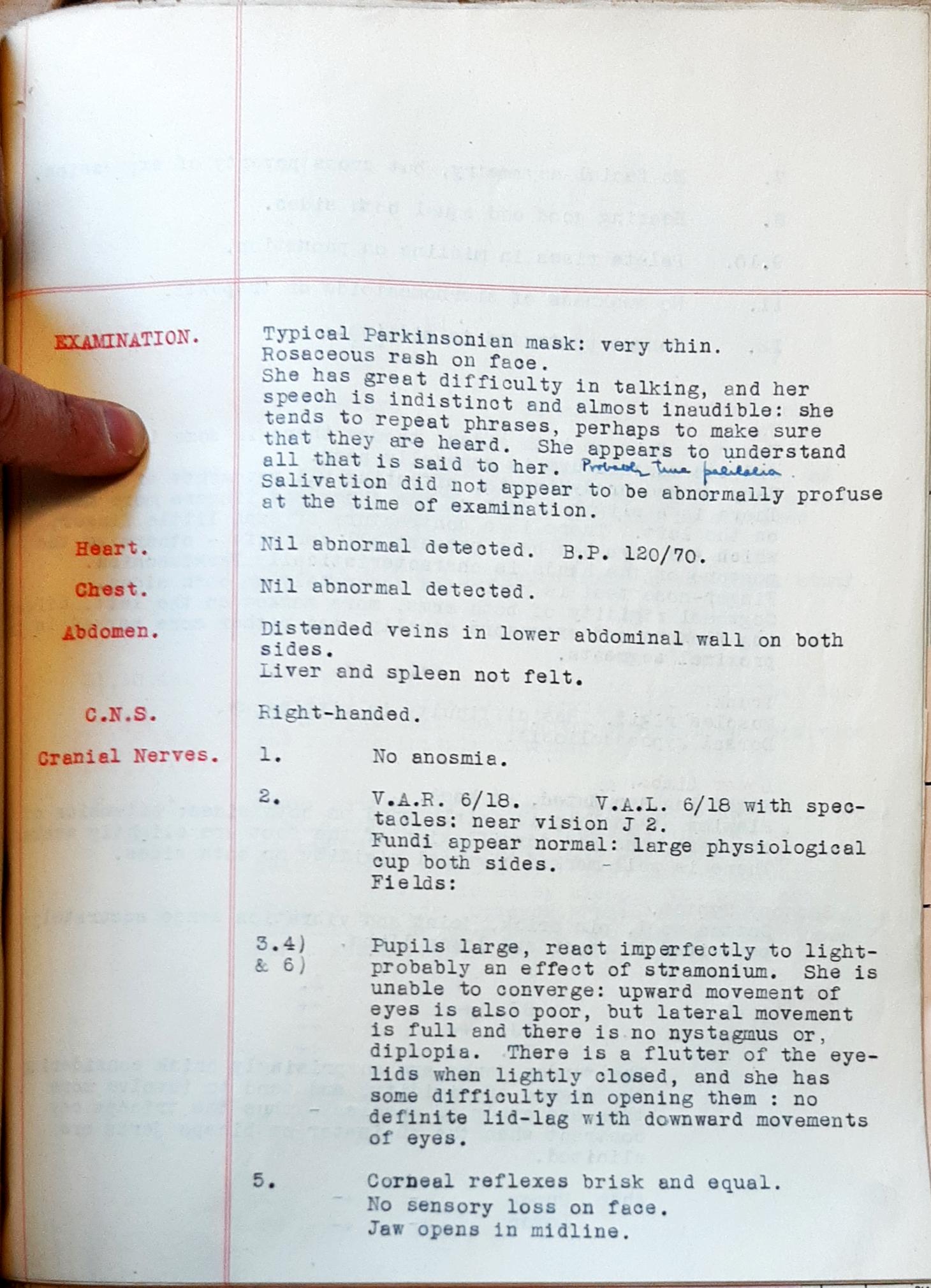


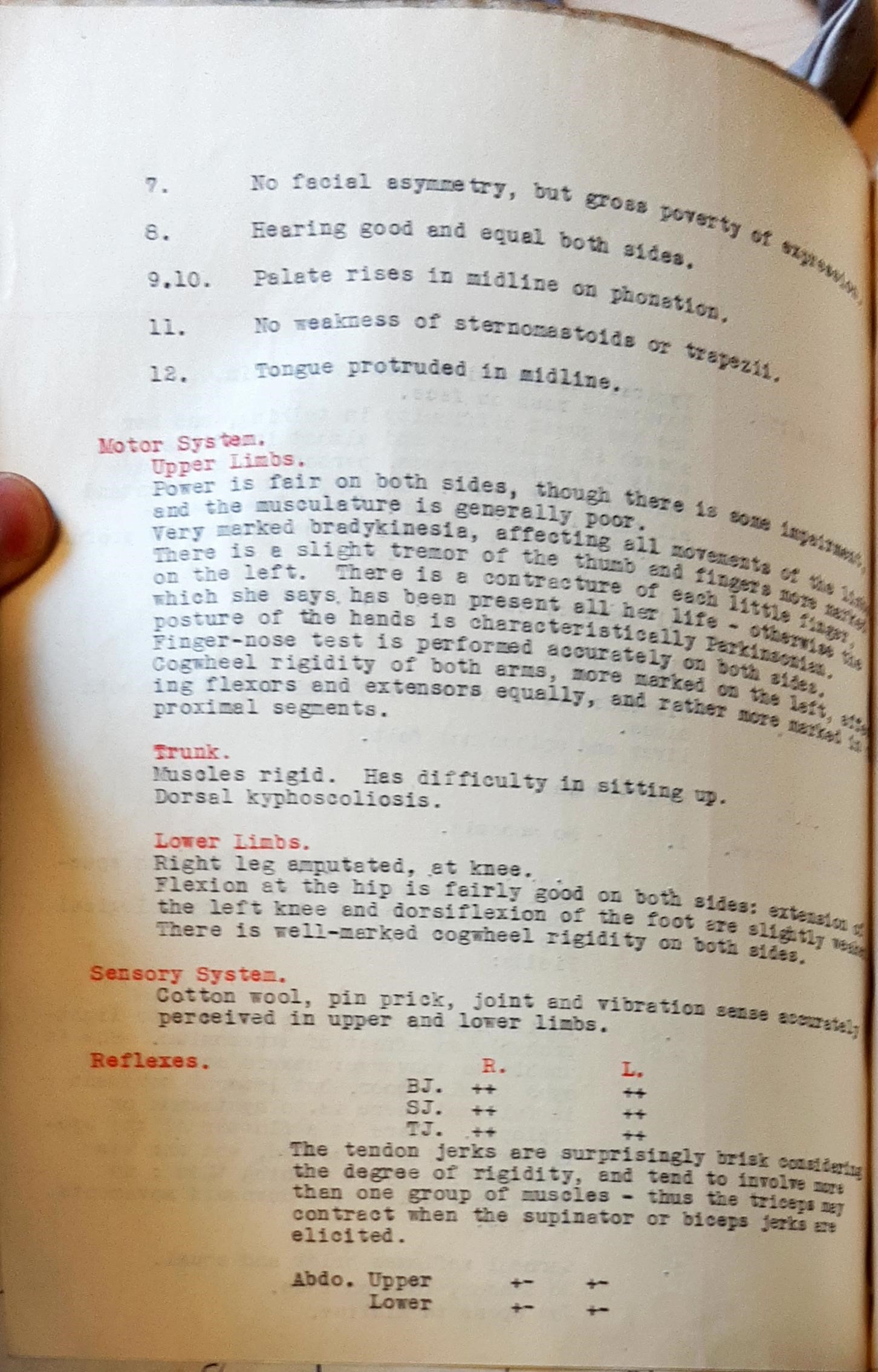


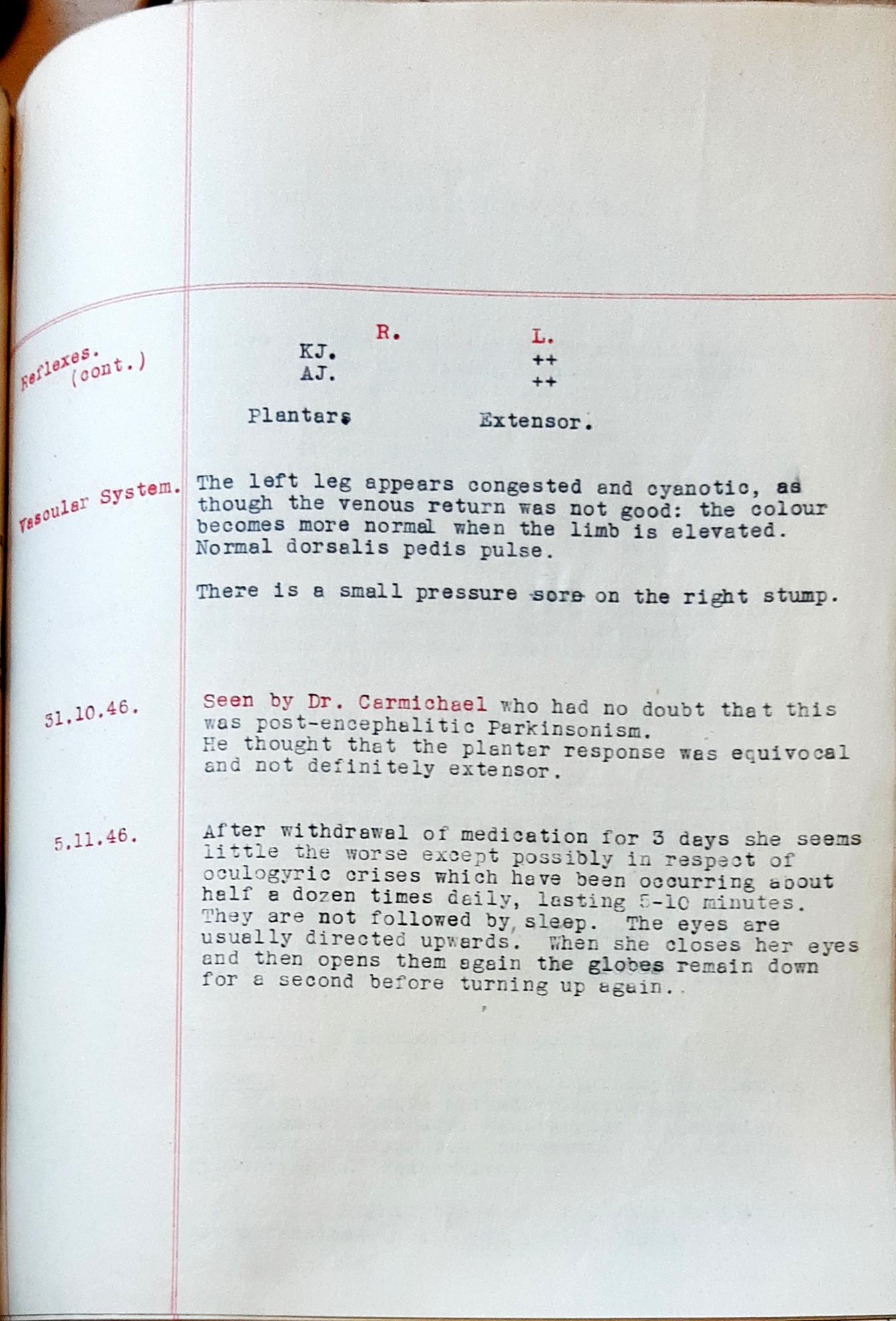


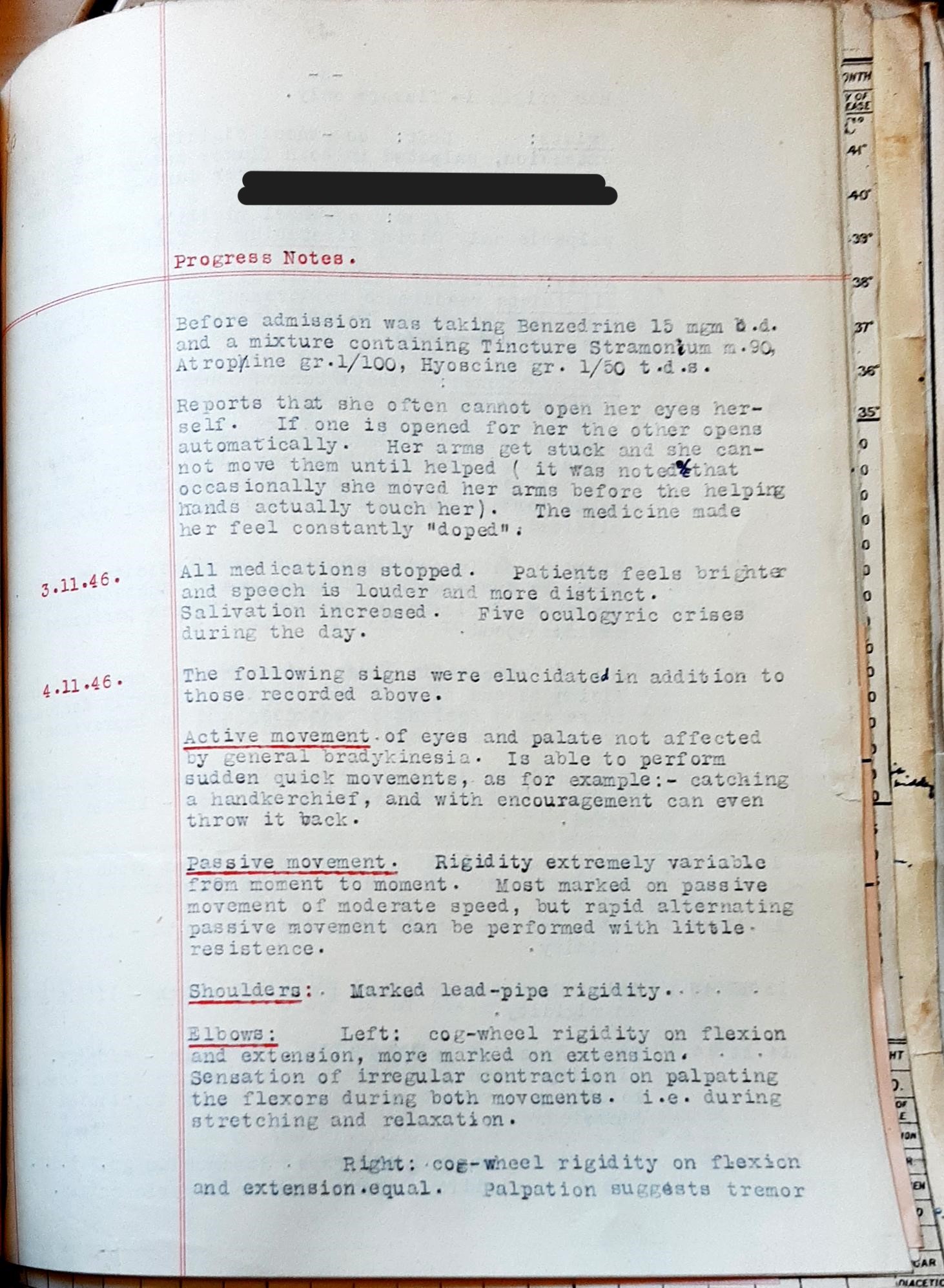


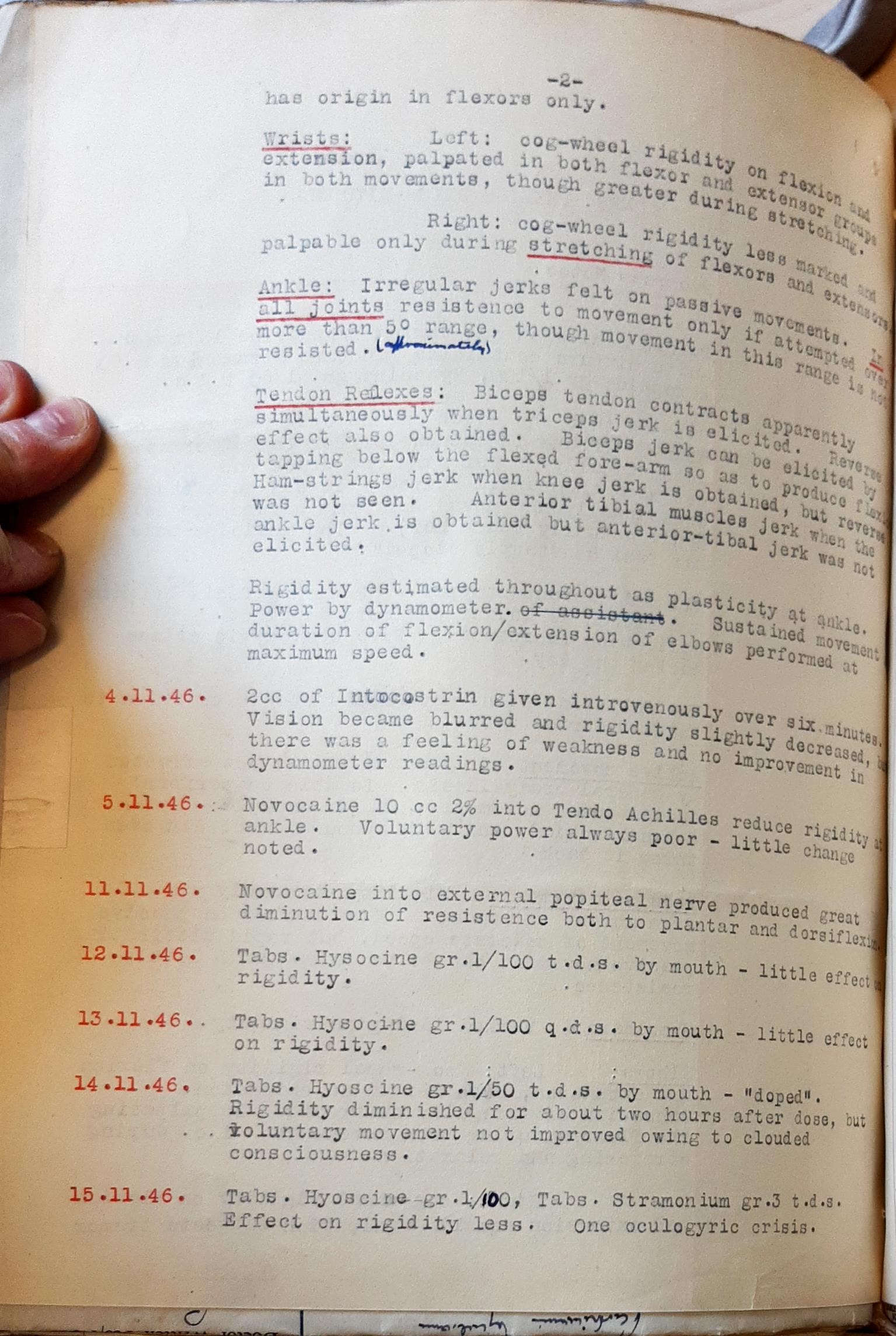


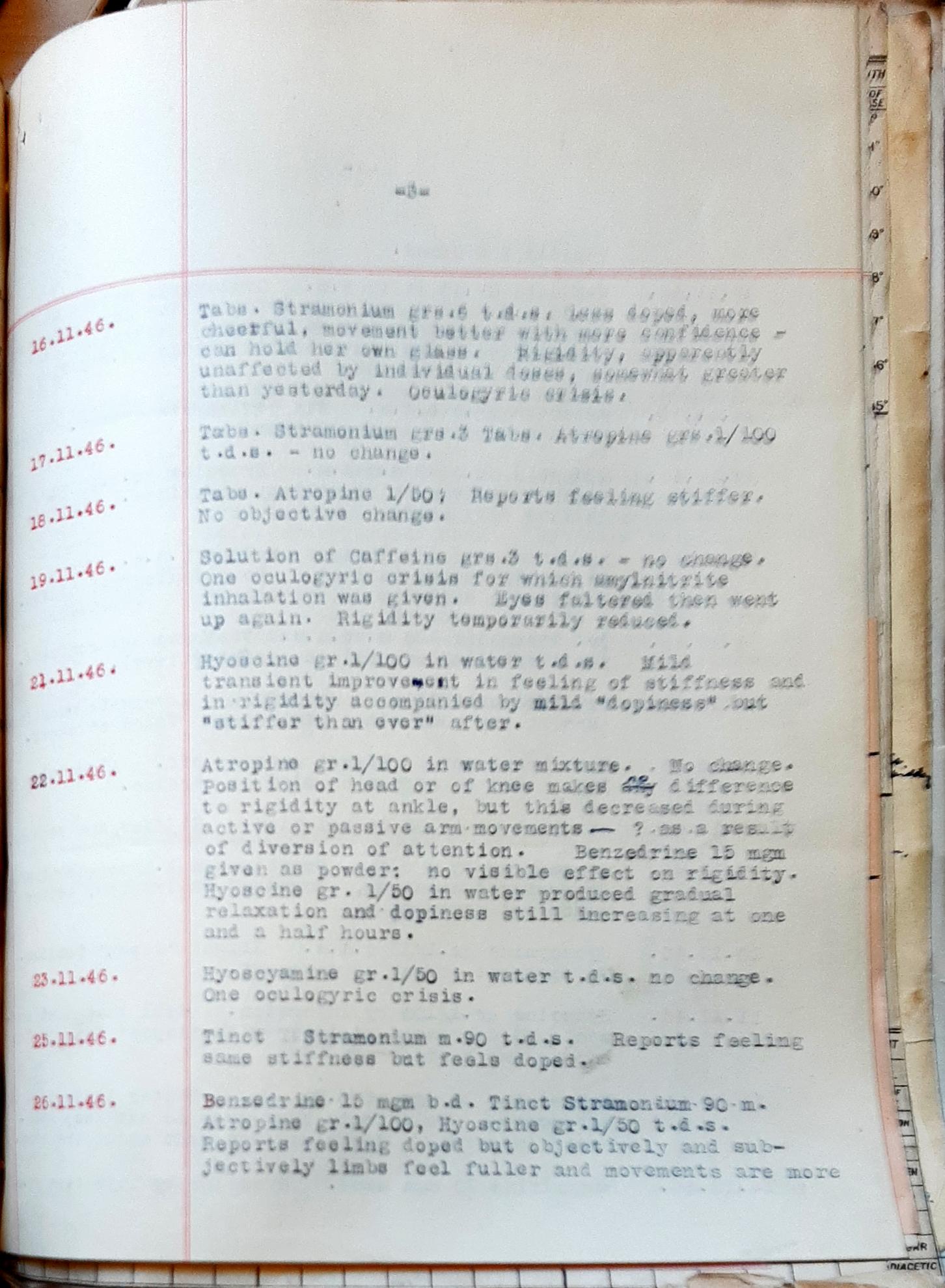


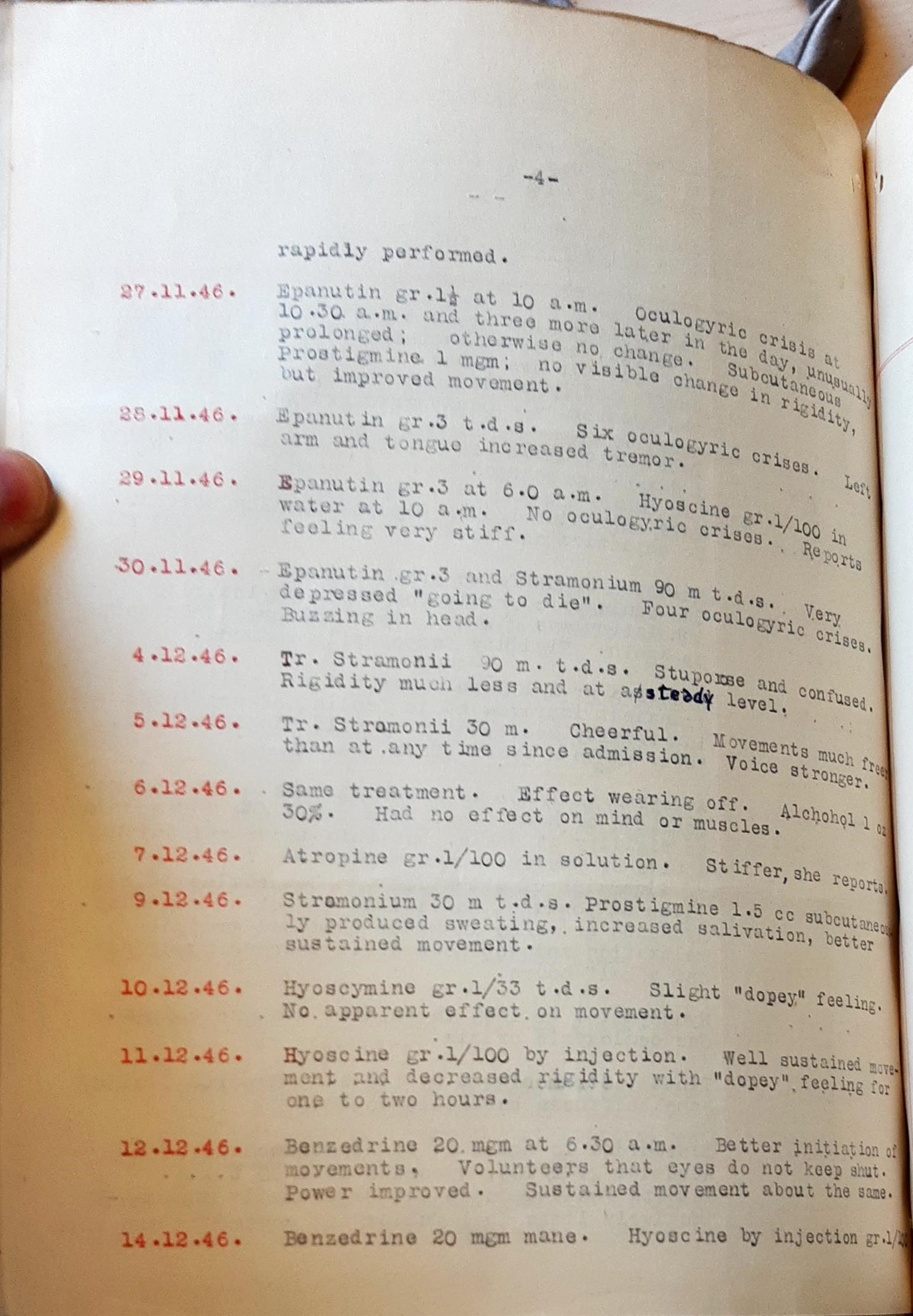


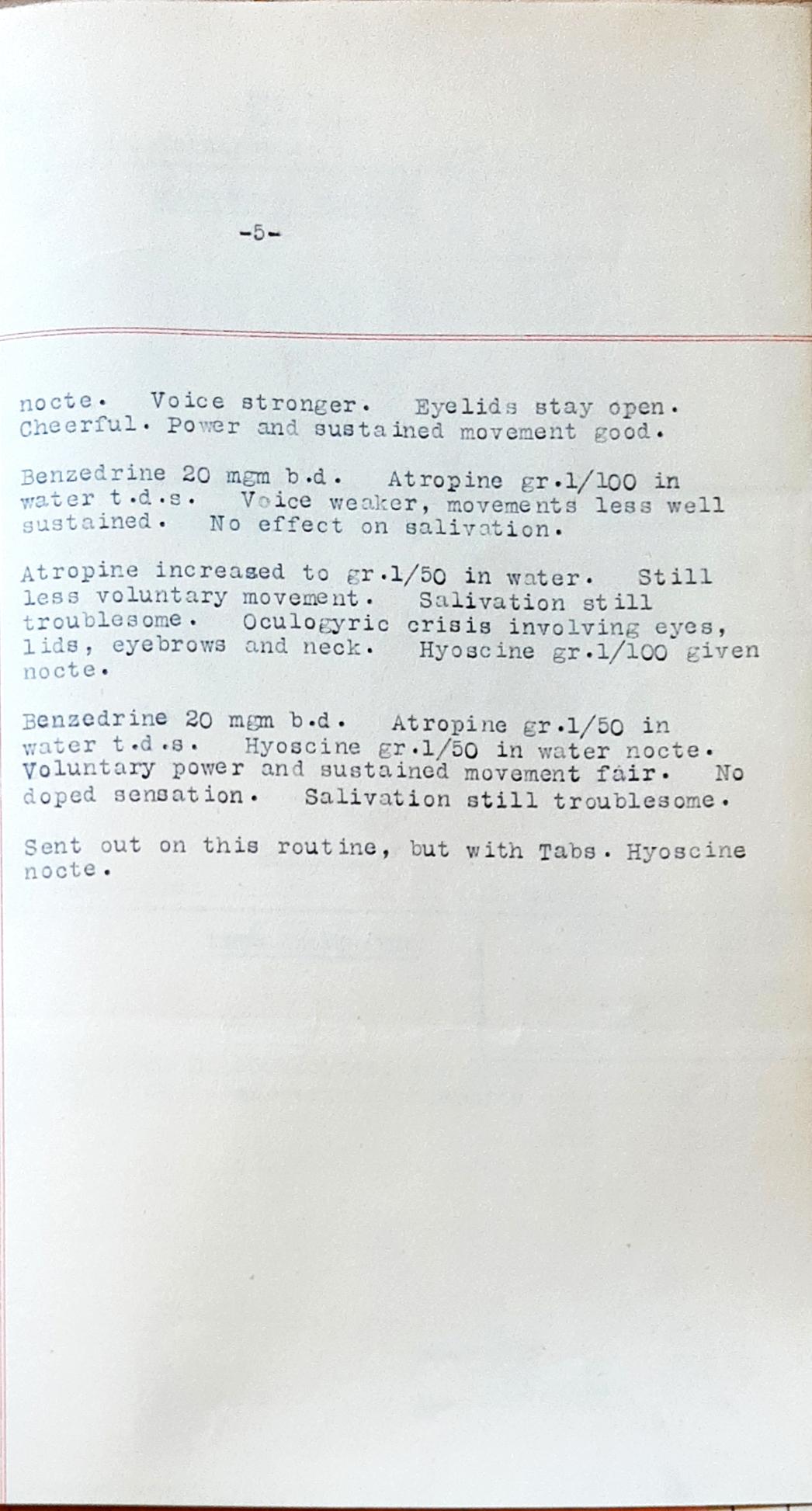


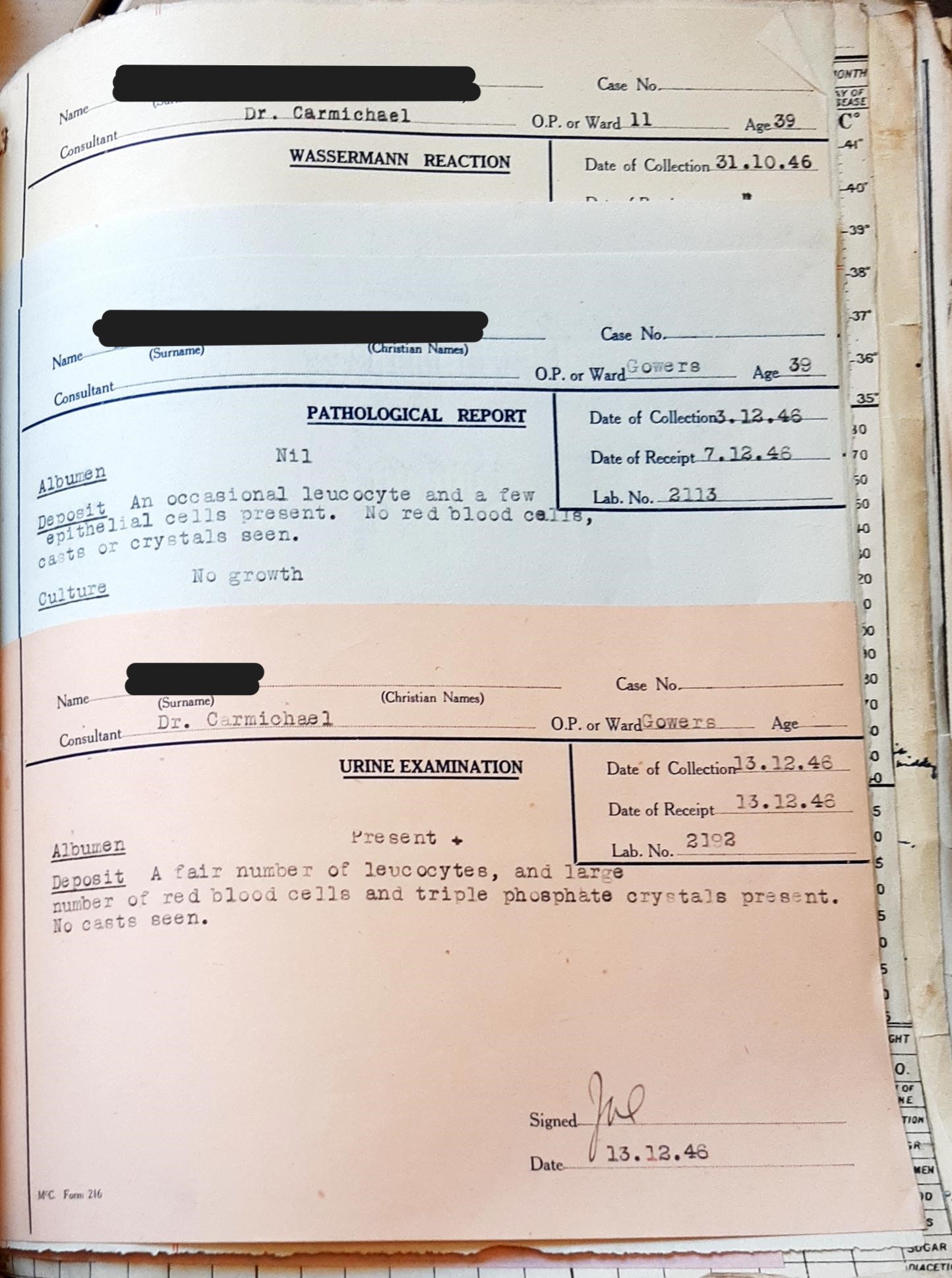


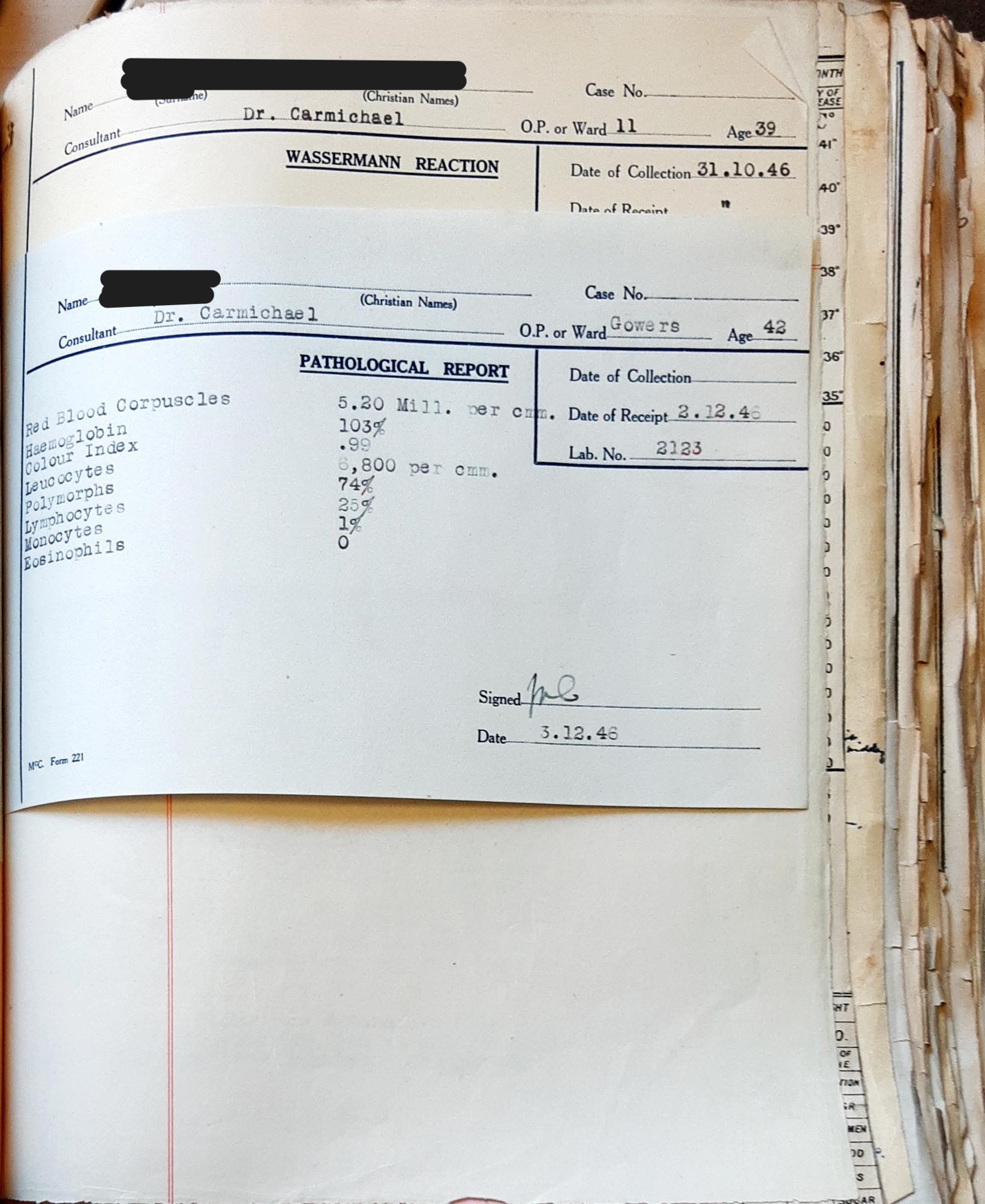


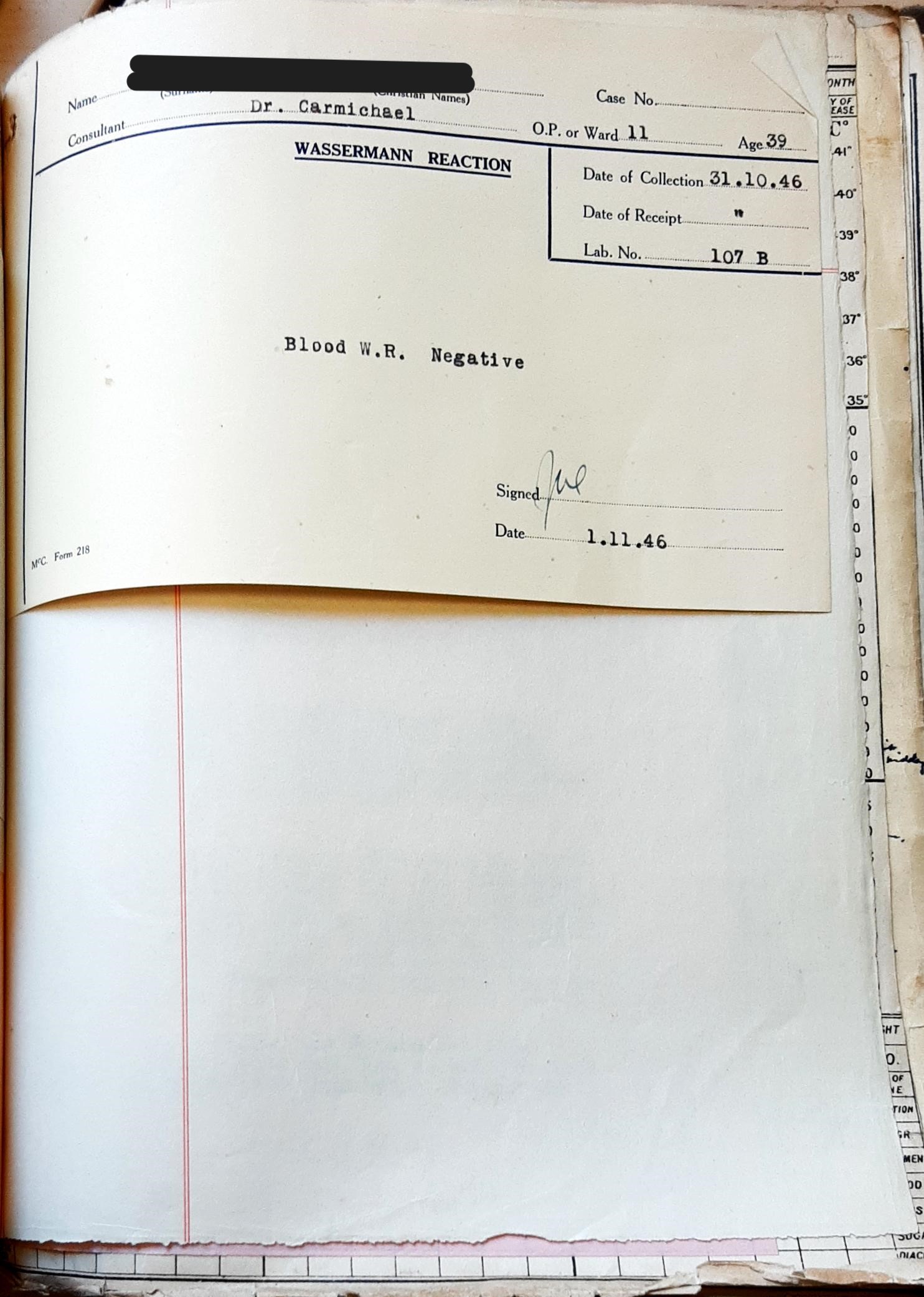


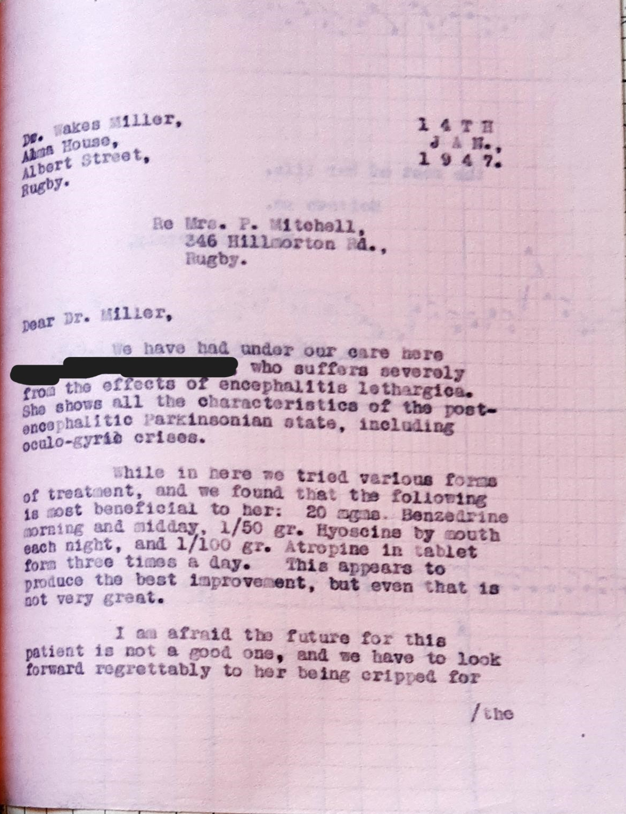


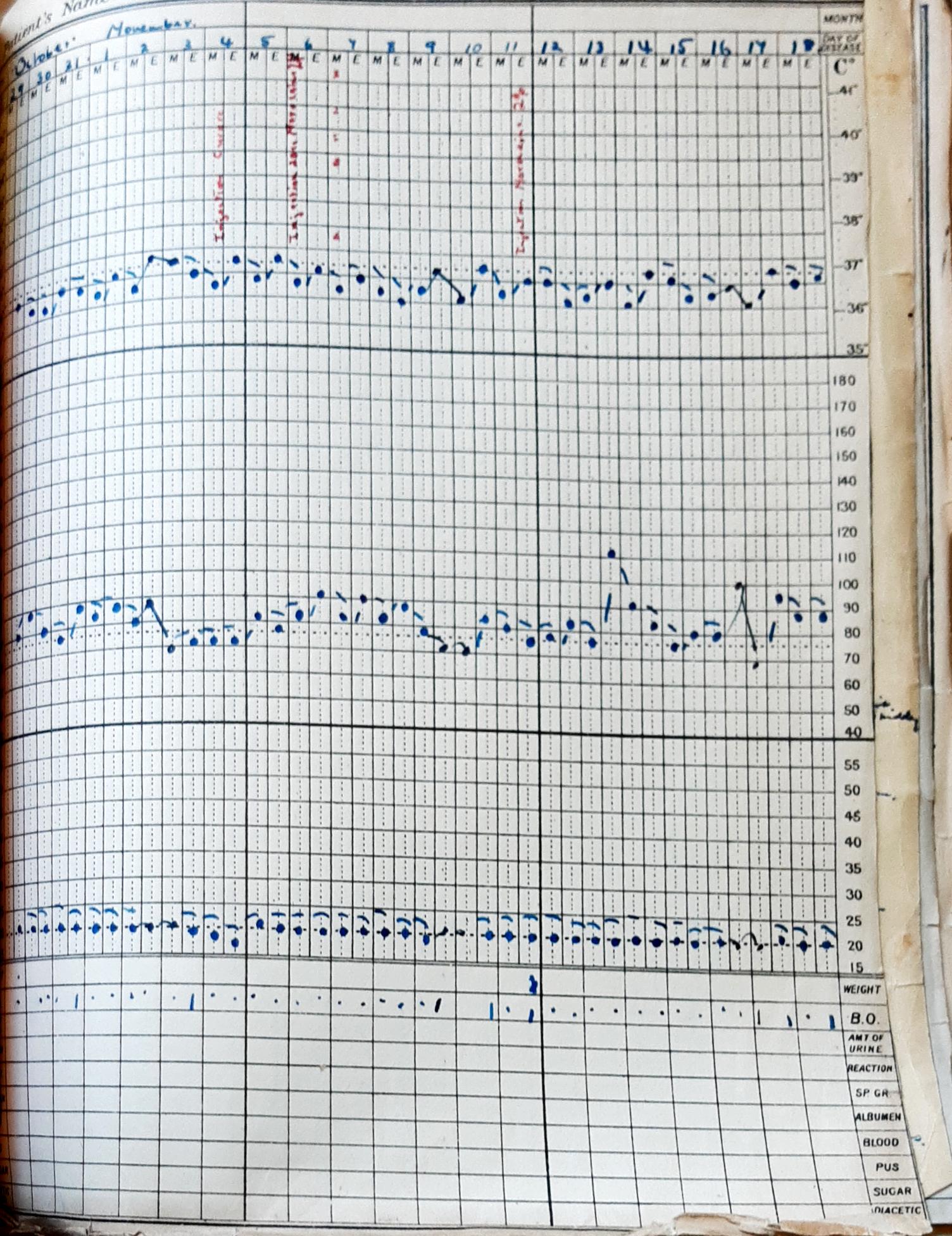


# Supplementary Figure 3: Violin plot of age at admission


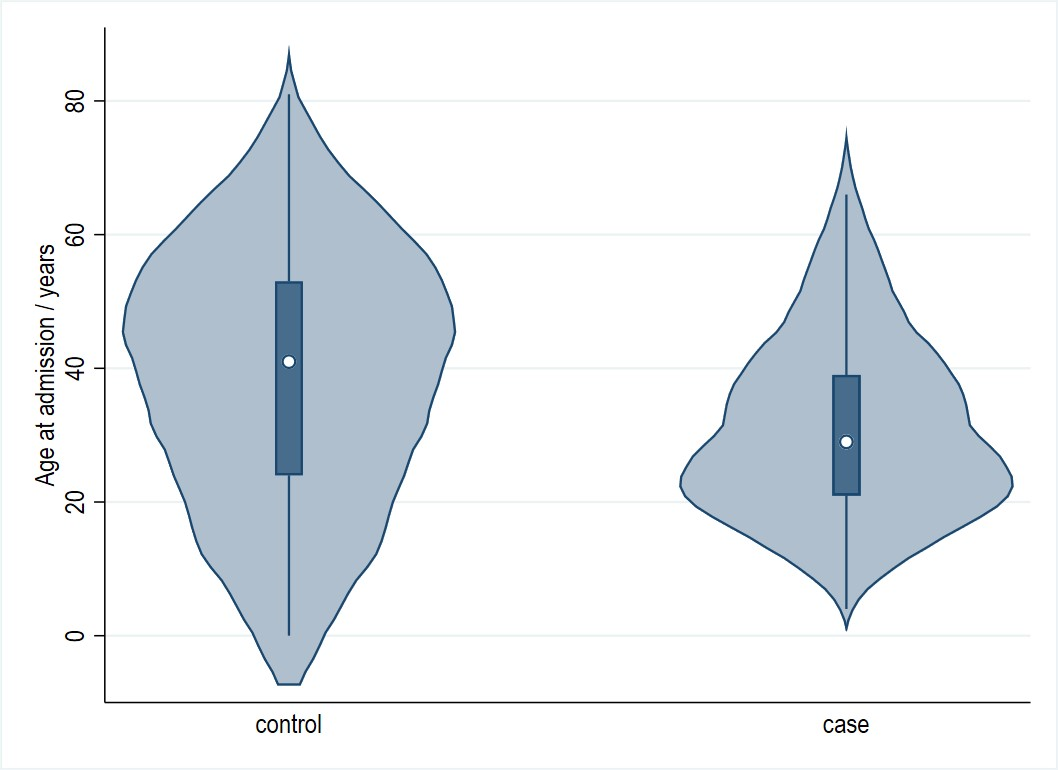


# Supplementary Figure 4: Violin plot of admission duration


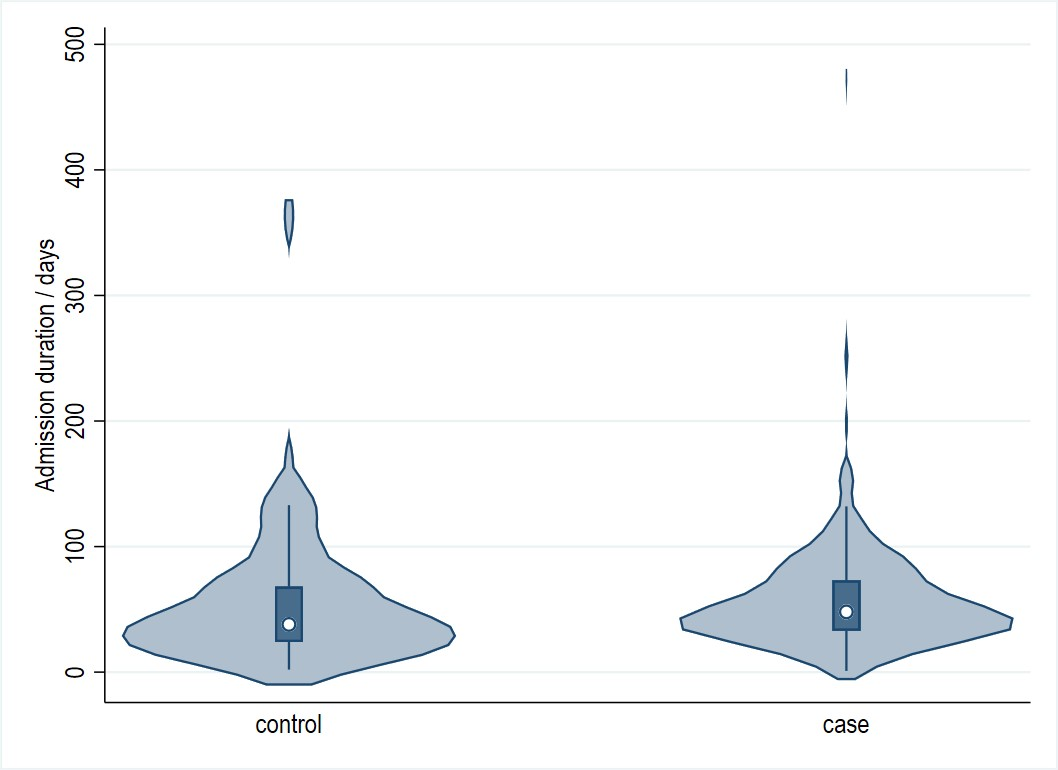


# Supplementary Figure 5: Violin plot of time from onset of neurological or neuropsychiatric symptoms to onset of parkinsonism


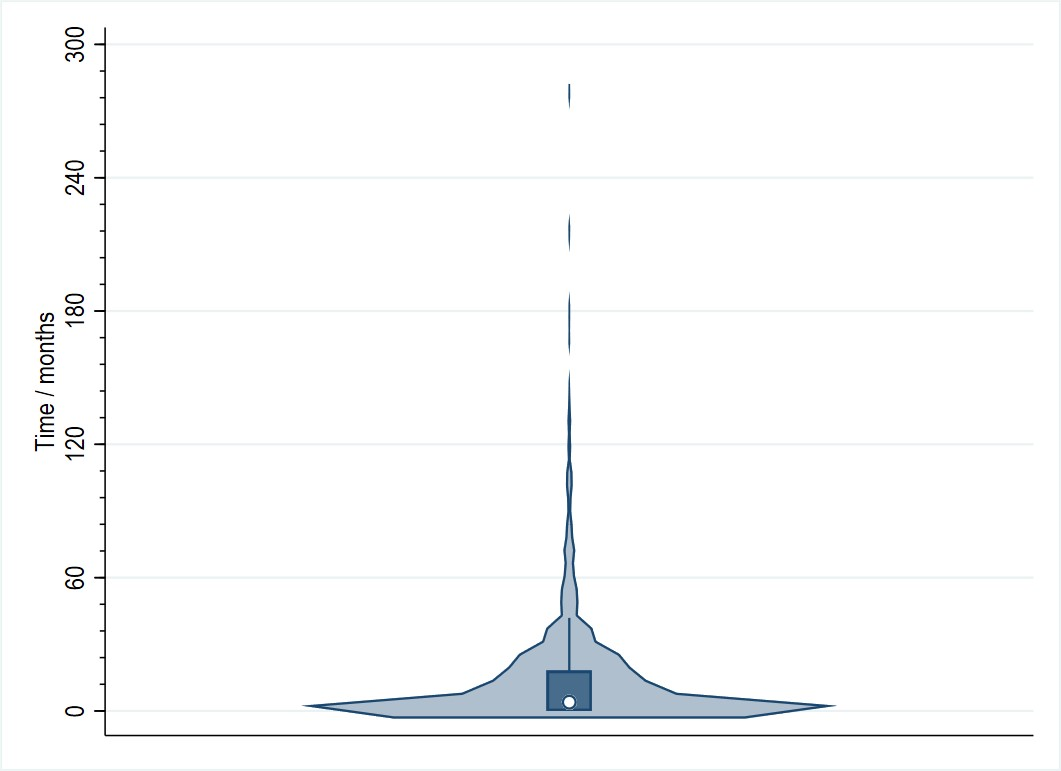


#
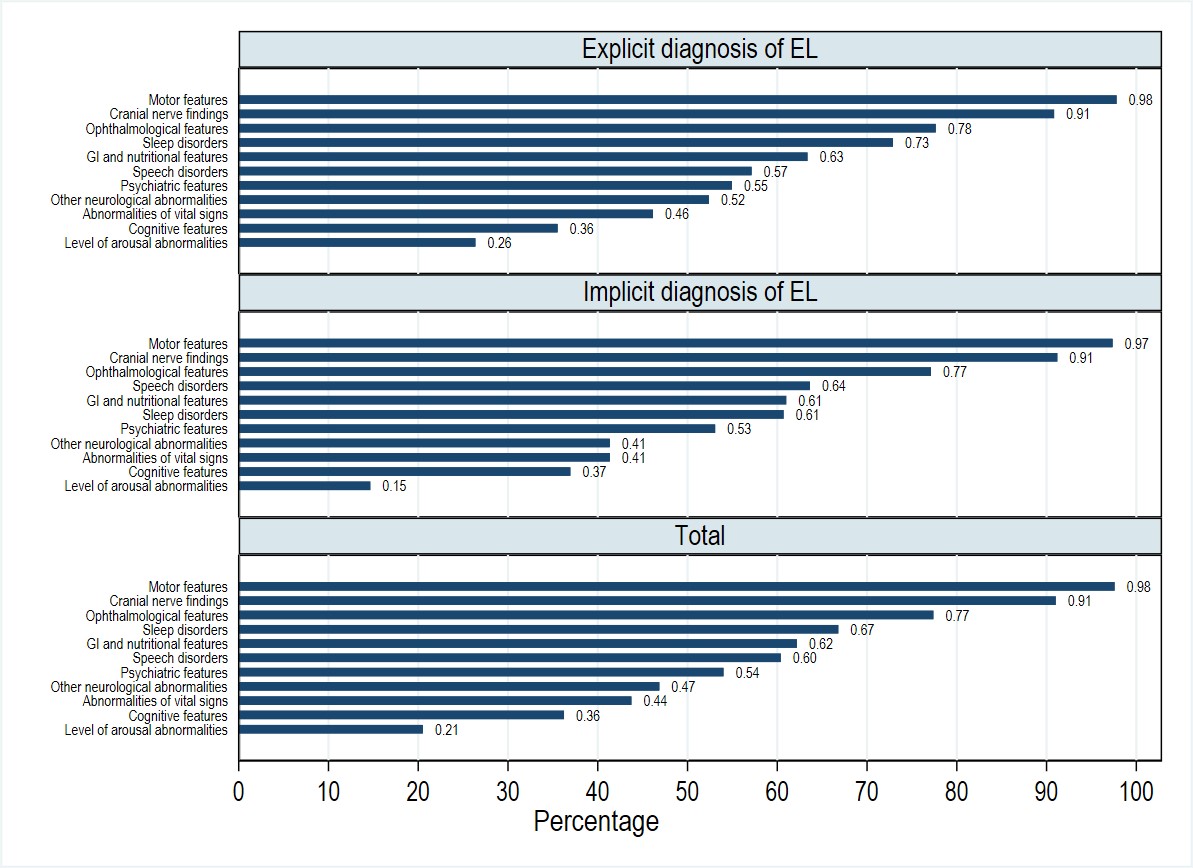
Supplementary Figure 6: Frequencies of categories of clinical features where diagnosis of encephalitis lethargica was implicit and explicit

# Supplementary References

1. International Labour Organization. *International Standard Classification of Occupations (ICSO), ISCO-8*. https://www.ilo.org/public/english/bureau/stat/isco/

2. The National Statistics Socio-economic classification (NS-SEC) - Office for National Statistics. Accessed September 22, 2023. https://www.ons.gov.uk/methodology/classificationsandstandards/otherclassifications/thenationalstatisticssocioeconomicclassificationnssecrebasedonsoc2010

3. Royal College of Nursing. *Standards for Assessing, Measuring and Monitoring Vital Signs in Infants, Children and Young People*.; 2017.

4. Graus F, Titulaer MJ, Balu R, et al. A clinical approach to diagnosis of autoimmune encephalitis. *Lancet Neurol*. 2016;15(4):391-404. doi:10.1016/S1474-4422(15)00401-9

5. Eeg-Olofsson R. On the Value of the Pandy and Nonne-Apelt (nonne) Tests. *Acta Psychiatrica Scandinavica*. 1948;23(S50):42-45. doi:10.1111/j.1600-0447.1948.tb04018.x

6. THOMPSON LJ. INTERPRETATION OF THE “PARETIC CURVE” IN LANGE’S COLLOIDAL GOLD TEST. *Archives of Neurology & Psychiatry*. 1921;5(2):131-145. doi:10.1001/archneurpsyc.1921.02180260020003

7. Bialynicki-Birula R. The 100th anniversary of Wassermann-Neisser-Bruck reaction. *Clin Dermatol*. 2008;26(1):79-88. doi:10.1016/j.clindermatol.2007.09.020
